# Using Automated Machine Learning to Predict Necessary Upcoming Therapy Changes in Patients With Psoriasis Vulgaris and Psoriatic Arthritis and Uncover New Influences on Disease Progression: Retrospective Study

**DOI:** 10.2196/55855

**Published:** 2024-06-27

**Authors:** Daniel Schaffert, Igor Bibi, Mara Blauth, Christian Lull, Jan Alwin von Ahnen, Georg Gross, Theresa Schulze-Hagen, Johannes Knitza, Sebastian Kuhn, Johannes Benecke, Astrid Schmieder, Jan Leipe, Victor Olsavszky

**Affiliations:** 1 Department of Dermatology, Venereology and Allergology University Medical Center and Medical Faculty Mannheim, University of Heidelberg, and Center of Excellence in Dermatology Mannheim Germany; 2 Department of Medicine V Division of Rheumatology University Medical Center and Medical Faculty Mannheim Mannheim Germany; 3 Institute of Digital Medicine Philipps-University Marburg and University Hospital of Giessen and Marburg Marburg Germany; 4 Department of Dermatology, Venereology, and Allergology University Hospital Würzburg Würzburg Germany

**Keywords:** psoriasis vulgaris, psoriatic arthritis, automated machine learning, therapy change, Psoriasis Area and Severity Index, PASI score change, Bath Ankylosing Spondylitis Disease Activity Index, BASDAI classification, mobile phone

## Abstract

**Background:**

Psoriasis vulgaris (PsV) and psoriatic arthritis (PsA) are complex, multifactorial diseases significantly impacting health and quality of life. Predicting treatment response and disease progression is crucial for optimizing therapeutic interventions, yet challenging. Automated machine learning (AutoML) technology shows promise for rapidly creating accurate predictive models based on patient features and treatment data.

**Objective:**

This study aims to develop highly accurate machine learning (ML) models using AutoML to address key clinical questions for PsV and PsA patients, including predicting therapy changes, identifying reasons for therapy changes, and factors influencing skin lesion progression or an abnormal Bath Ankylosing Spondylitis Disease Activity Index (BASDAI) score.

**Methods:**

Clinical study data from 309 PsV and PsA patients were extensively prepared and analyzed using AutoML to build and select the most accurate predictive models for each variable of interest.

**Results:**

*Therapy change at 24 weeks* follow-up was modeled using the extreme gradient boosted trees classifier with early stopping (area under the receiver operating characteristic curve [AUC] of 0.9078 and logarithmic loss [LogLoss] of 0.3955 for the holdout partition). Key influencing factors included the initial systemic therapeutic agent, the Classification Criteria for Psoriatic Arthritis score at baseline, and changes in quality of life. An average blender incorporating three models (gradient boosted trees classifier, ExtraTrees classifier, and Eureqa generalized additive model classifier) with an AUC of 0.8750 and LogLoss of 0.4603 was used to predict therapy changes for 2 hypothetical patients, highlighting the significance of these factors. Treatments such as methotrexate or specific biologicals showed a lower propensity for change. An average blender of a random forest classifier, an extreme gradient boosted trees classifier, and a Eureqa classifier (AUC of 0.9241 and LogLoss of 0.4498) was used to estimate *PASI (Psoriasis Area and Severity Index) change after 24 weeks*. Primary predictors included the initial PASI score, change in pruritus levels, and change in therapy. A lower initial PASI score and consistently low pruritus were associated with better outcomes. *BASDAI classification at onset* was analyzed using an average blender of a Eureqa generalized additive model classifier, an extreme gradient boosted trees classifier with early stopping, and a dropout additive regression trees classifier with an AUC of 0.8274 and LogLoss of 0.5037. Influential factors included initial pain, disease activity, and Hospital Anxiety and Depression Scale scores for depression and anxiety. Increased pain, disease activity, and psychological distress generally led to higher BASDAI scores.

**Conclusions:**

The practical implications of these models for clinical decision-making in PsV and PsA can guide early investigation and treatment, contributing to improved patient outcomes.

## Introduction

### Background

Psoriasis is a chronic inflammatory skin disease that is on the rise and is becoming increasingly visible in everyday clinical practice [1]. Its prevalence in adults is between 2% and 3% worldwide, while children are less frequently affected [2,3]. Psoriasis can present clinically in different forms, with the most common form being psoriasis vulgaris (PsV). PsV consists of papulosquamous plaques typically appearing on the extensor sides of the extremities, scalp, lumbosacral areas, and umbilicus [4]. Other forms are subdivided according to the appearance of efflorescences or according to the affected regions of the body. Remarkably, nail psoriasis also seems to be an indicator of systemic manifestation of PsV [5,6]. Almost 30% of the people with psoriasis can develop a systemic involvement called psoriatic arthritis (PsA) that manifests at the joints and tendon attachments [7]. This seronegative arthritis is difficult to diagnose due to its heterogeneous appearance with peripheral joint involvement, axial joint involvement, tendonitis, enthesitis, and even dactylitis [8,9]. If left untreated, PsA can lead to debilitating and irreversible joint and bone deformities [10]. Alongside PsA, patients with PsV also show an increased risk of cardiovascular and metabolic diseases, such as coronary artery disease, arterial hypertension, atherosclerosis, type 2 diabetes mellitus, or obesity, with all of these contributing to an increased mortality rate [11,12].

Modern PsV therapy includes topical treatments (such as corticosteroids and vitamin D analogs); phototherapy (especially narrow-band UV-B for various forms of psoriasis); and systemic therapies for moderate to severe cases, including methotrexate (MTX) and newer biologics targeting tumor necrosis factor alpha (TNF-α), interleukin (IL)–17, and IL-23. As MTX is also used in the therapy of rheumatic diseases, including PsA, it is also considered a conventional synthetic disease-modifying antirheumatic drug (csDMARD). Biologic disease-modifying antirheumatic drugs, recommended for severe PsV or when other treatments fail [13], have transformed disease management, particularly as first-line options in certain guidelines [14]. Despite these advances, complete cure remains elusive, and treatment remains a challenge [15].

While the efficacy of modern PsV and PsA treatments has improved significantly, the variability of patient response remains a challenge, with outcomes ranging from remission to disease progression [16,17]. Moreover, it is difficult to adequately assess the therapy success in day-to-day hospital routine because there are many factors that affect the response to therapy [18]. These factors include health parameters (such as sex, BMI, and preexisting conditions), lifestyle choices, and individual tolerance to therapy. Previous studies have shown that women and nonobese patients often respond better to biologics, which may require weight-adjusted dosing [19-21]. Preexisting conditions also guide the choice of biologic therapy [22], with certain systemic conditions constituting contraindications. Lifestyle factors such as drug use, smoking, exercise, and diet can affect disease severity and treatment efficacy. Finally, the therapy itself is differently tolerated by each patient. Common side effects such as headaches, diarrhea, malaise, or injection site reactions are sometimes perceived as highly unendurable by some patients, thus leading to an inevitable change in medical treatment [23].

Psoriasis treatments typically take several weeks to become fully effective, delaying the recognition of PsV or PsA remission. In addition, individual patient needs and expectations vary, with younger patients prioritizing successful treatment, while older adults value sleep and easy medical access [24]. Over time, patient preferences adapt to treatment experiences [25]. Thus, unmet treatment goals can exacerbate physical symptoms and cause psychosocial distress [26], leading to depression and social repercussions [27]. These patients also report reduced quality of life (QoL) and relationship problems [28,29]. Therefore, apart from psoriasis treatments and psychological aids, tools to predict individual disease progression are lacking due to these complex factors influencing psoriasis. However, advances in digital patient data collection and revolutionary new technologies such as machine learning (ML) offer promise for improving the effectiveness of therapy by tailoring it to patients’ conditions.

ML allows its users to analyze big data sets by massive processing and uncover new insights that remain otherwise undisclosed when using common statistical evaluation. ML has already been implemented successfully in medicine for several years because it can especially identify nonlinear relationships between the parameters (ie, features) of medical data sets [30]. The biggest problem so far is the lack of expertise on ML in the medical sector. Without such expertise, it is difficult to develop a functioning ML model that works outside of the analyzed data set predictions [31]. To resolve this issue, a novel technology termed automated ML (AutoML) was developed. AutoML allows its users to test and ultimately use ML models independently, in a short time, without the need of expert knowledge [31]; for example, its benefit was evident during the COVID-19 pandemic. Here, AutoML was used to distinguish between COVID-19 pneumonia, non–COVID-19 pneumonia, and healthy chest x-ray images [32,33]. Furthermore, AutoML was successfully used to predict the number of intensive care unit beds required during the pandemic as well as to predict COVID-19 disease course [34,35]. ML is also widely used in the medical field of dermatology, where the majority of use cases are image based [36,37]. In the study of psoriasis, different ML methods such as convolutional neural networks (CNNs) [38,39], U-Net [40,41], and deep CNNs [42,43] have been used. Their accuracies ranged from as high as 0.9877 [40] for some models to as low as 0.6030 [39] for others. These models have been applied to various tasks, including lesion classification [38], body surface area measurement [41], and severity assessment [40]. Given that existing models have shown varying accuracies, new AutoML analyses could discover more efficient algorithms or parameter configurations that improve predictive performance and automatically identify features that are informative about the conditions in question, leading to more insightful models. However, to this date, dermatological medical data sets have been scarcely analyzed with AutoML.

### Objectives

In this study, we set out to perform AutoML analyses of a dermatological data set of patients with PsV and PsA. The goal was to uncover unknown relationships between therapeutic responses and individual patient parameters. The ultimately selected, highly accurate ML models could potentially serve as reliable predictors of psoriasis disease progression in the future with their integration into daily medical routine.

## Methods

### Data Source

Two independent clinical studies on patients with PsV and PsA using a monitoring smartphone app were carried out between 2018 and 2021 at our department of dermatology. The first clinical trial recruited a total of 107 patients with PsV from 2018 to 2020 [44,45]. Almost half of the participants had an additional diagnosis of PsA. Study patients underwent an educational program, used a medical study app to document their disease activity, and had 5 follow-up appointments at 4, 12, 24, 36, and 60 weeks after study inclusion. General health parameters were gathered, and multiple questionnaires were completed at study onset and follow-ups. Patient data consisted of lifestyle and social parameters, medical parameters, disease activity scores, comorbidities, and therapeutic parameters. In addition, further data were continuously gathered via the study app, with patients documenting pain and pruritus symptoms as well as their current Dermatology Life Quality Index (DLQI) score. Taken together, this primary data set consisted of 135 different patient parameters, hereinafter referred to as features.

The second clinical study was carried out in 2020-2021 and comprised 202 patients with both PsV and PsA [46]. All patients were treated during an interdisciplinary dermatological-rheumatological consultation at our department of dermatology and were granted access to the same monitoring study app. Again, questionnaires were completed, and general health parameters, disease activity scores, and therapeutic features were recorded each time at study onset and at follow-ups 12 and 24 weeks later. With the addition of further PsA parameters and scores, this second primary data set encompassed 531 different features per patient. As both clinical trials used the same monitoring smartphone app and were undertaken under similar conditions by the same clinical investigation team, we used their primary data sets to extract all common features to create a secondary data set for AutoML analysis.

### Data Preparation

A secondary data set was created by merging patients with common features from both primary data sets of the aforementioned clinical trials (Multimedia Appendix 1). This new retrospective data set included a total of 309 patients with PsV with or without PsA, with 82 different features per patient. Of these 309 patients, 111 (35.9%) were diagnosed with PsA. Basic cohort characteristics of the secondary data set are shown in Multimedia Appendix 2. Incomplete features and data from follow-up appointments that were only collected in the first clinical study at 4, 36, and 40 weeks were excluded. Specifically, this new data set consisted of data gathered at study onset and at 12- and 24-week follow-ups, with data features being broadly categorized into personal data, mobile data, and medical scores (Textbox 1).

**Detailed categorization and timeline of the recorded data features in the presented psoriasis vulgaris and psoriatic arthritis studies. This textbox provides an aggregated list of the data features used in the analysis, divided into 3 primary groups: personal data demographics, mobile app data, and standardized medical scores. The personal demographics and medical scores include initial assessments and follow-up data at 12- and 24-week intervals. The continuous mobile app data reflect the engagement and input of patient participants, which have been averaged for consistency. Key patient-reported outcomes include pain, pruritus, and the Dermatology Life Quality Index (DLQI), which were collected both via the mobile app by the patients themselves and via in-clinic assessments by the investigators at the initial visit and follow-up appointments.**

**Personal data (collected at study onset and updated at 12- and 24-week follow-ups)**

- Sex
- Age
- Body height
- Body weight
- BMI
- Smoking status
- Alcohol consumption
- Preexisting illnesses
- Sports activities (at least 2 h/wk)
- Occupation
- Medication (systemic and topical therapy)
- Allergies
- Comorbidities
- Psoriatic arthritis (yes or no)

**Mobile app data (continuously collected and averaged)**

- App used (yes or no)
- Day counts of app use
- Total counts of answered questions
- Counts of daily answered questions
- Average pain score (numeric rating scale [NRS])
- Average pruritus score (NRS)
- Average Dermatology Life Quality Index (DLQI) score
- Average of mood tracking
- Average of daily activity assessment
- Average morning stiffness intensity
- Average morning stiffness duration

**Medical scores (evaluated at study onset and updated at 12- and 24-week follow-ups)**

- Pain (NRS)
- Pruritus (NRS)
- DLQI score
- Hospital Anxiety and Depression Scale (HADS) anxiety
- HADS depression
- Psoriasis Area and Severity Index
- Classification Criteria for Psoriatic Arthritis
- Bath Ankylosing Spondylitis Disease Activity Index
- Disease activity (NRS)

New features were created and calculated to further enrich the data set; for example, BMI was calculated by using the patient’s height and weight, physical activity level was assessed using the patient’s occupation and sports activities, and daily app use and daily questions answered were calculated by dividing the total number of app use instances and questions answered by the total number of days the app was used. In addition, selected features were additionally transformed from numeric to binary or multiclass classification to facilitate subsequent AutoML analysis and interpretation of the results (Multimedia Appendix 3). For this purpose, the Bath Ankylosing Spondylitis Disease Activity Index (BASDAI), Hospital Anxiety and Depression Scale (HADS), and DLQI were reclassified into different categories after passing certain score thresholds, and patients’ occupations were categorized into specific job types. However, the original features were also retained to subsequently assess which feature version provided better insights during AutoML processing. Finally, we calculated feature changes over time at follow-up and also classified them into binary or multiclass feature types (Table 1). To facilitate the machine learning analysis, several classes were defined in the multiclass feature *PASI change after 24 weeks*. First, a cutoff was set at PASI score=3. Values ≤3 were considered consistently low disease activity. Values >3 were considered active psoriasis. A PASI score reduction, together with PASI75 (75% improvement in the PASI score from baseline) and PASI90 (90% improvement in the PASI score from baseline), was considered a low reduction if the baseline value was >30% higher than the final value. If the baseline value was higher than the final value but not >30%, this was considered to be no significant change. An increase in the PASI score was considered PASI score progression regardless of the value of the increase; if the baseline value was ≤3 and the final value was >3, this was considered a clinical psoriasis progression and classified separately. Data were not normalized during data preparation. The pseudonymized secondary data set with common and newly calculated features is deposited on the web [47].

**Table 1:** Overview of common features for which change over time was calculated in a new additional feature. For all scores collected at the beginning and end of the study at the follow-up visits, the change over time was recorded in a new feature. The scores were categorized according to their values. The categorization criteria are given in the New classification over time column. Within the feature DLQI [Dermatology Life Quality Index] classification change over 24 weeks, the class Consistently best quality of life was defined but contained no calculable data points. The feature types were multiclass, except for the binary Psoriasis Area and Severity Index (PASI) score change.

Feature name	Feature type	New classification over time
Pain change over 24 weeks	Multiclass	Constantly free of pain (0/10 NRS^a^ score at study onset and after 24 weeks) Constant low pain (≤4/10 NRS score at study onset and at 24 weeks) Constant moderate pain (>4/10 and ≤7/10 NRS score at study onset and after 24 weeks) Constant high pain (>7/10 NRS score at study onset and after 24 weeks) Increase in pain (0/10 NRS score at study onset and >0/10 NRS score at 24 weeks, ≤4/10 NRS score at study onset and >4/10 NRS score at 24 weeks, or ≤7/10 NRS score at study onset and >7/10 NRS score at 24 weeks) Decrease in pain (>0/10 NRS score at study onset and 0/10 NRS score after 24 weeks, >4/10 NRS score at study onset and ≤4/10 NRS score after 24 weeks, or >7/10 NRS score at study onset and ≤7/10 NRS score after 24 weeks)
Pruritus change over 24 weeks	Multiclass	Constantly free of pruritus (0/10 NRS score at study onset and after 24 weeks) Constant low pruritus (≤4/10 NRS score at study onset and ≤4/10 NRS score after 24 weeks) Constant moderate pruritus (>4/10 and ≤7/10 NRS score at study onset and after 24 weeks) Constant high pruritus (>7/10 NRS score at study onset and after 24 weeks) Increase in pruritus (0/10 NRS score at study onset and >0/10 NRS score after 24 weeks, ≤4/10 NRS score at study onset and >4/10 NRS score after 24 weeks, or ≤7/10 NRS score at study onset and >7/10 NRS score after 24 weeks) Decrease in pruritus (>0/10 NRS score at study onset and 0/10 NRS score after 24 weeks, >4/10 NRS score at study onset and ≤4/10 NRS score after 24 weeks, or >7/10 NRS score at study onset and ≤7/10 NRS score after 24 weeks)
DLQI classification change over 24 weeks	Multiclass	Consistently best quality of life (≤1/30 DLQI score at study onset and after 24 weeks) Consistently good quality of life (>1/30 and ≤5/30 DLQI score at study onset and after 24 weeks) Consistently mediocre quality of life (>5/30 and ≤10/30 DLQI score at study onset and after 24 weeks) Consistently poor quality of life (>10/30 DLQI score at study onset and after 24 weeks) Improved quality of life (>1/30 DLQI score at study onset and ≤1/30 DLQI score after 24 weeks, >5/30 DLQI score at study onset and ≤5/30 DLQI score after 24 weeks, or >10/30 DLQI score at study onset and ≤10/30 DLQI score after 24 weeks) Decrease in quality of life (≤1/30 DLQI score at study onset and >1/30 DLQI score after 24 weeks, ≤5/30 DLQI score at study onset and >5/30 DLQI score after 24 weeks, or ≤10/30 DLQI score at study onset and >10/30 DLQI score after 24 weeks)
HADS-A^b^ classification change over 24 weeks	Multiclass	Constantly inconspicuous (≤7/21 HADS score at study onset and after 24 weeks) Constantly borderline (>7/21 and ≤10/21 HADS score at study onset and after 24 weeks) Constantly suspicious (>11/21 HADS score at study onset and after 24 weeks) Increase in anxiety (≤7/21 HADS score at study onset and >7/21 HADS score after 24 weeks or ≤10/21 HADS score at study onset and >10/21 HADS score after 24 weeks) Decrease in anxiety (>7/21 HADS score at study onset and ≤7/21 HADS score after 24 weeks, or >10/21 HADS score at study onset and ≤10/21 HADS score after 24 weeks)
HADS-D^c^ classification change over 24 weeks	Multiclass	Constantly inconspicuous (≤7/21 HADS score at study onset and after 24 weeks) Constantly borderline (>7/21 and ≤10/21 HADS score at study onset and after 24 weeks) Constantly suspicious (>11/21 HADS score at study onset and after 24 weeks) Increase in depression (≤7/21 HADS score at study onset and >7/21 HADS score after 24 weeks, or ≤10/21 HADS score at study onset and >10/21 HADS score after 24 weeks) Decrease in depression (>7/21 HADS score at study onset and ≤7/21 HADS score after 24 weeks, or >10/21 HADS score at study onset and ≤10/21 HADS score after 24 weeks)
PASI change after 24 weeks (differential)	Multiclass	Constantly low PASI score (<3/72 PASI score at study onset and after 24 weeks) PASI75 (PASI score at study onset >0.25×PASI score after 24 weeks) PASI90 (PASI score at study onset >0.1×PASI score after 24 weeks) Minor PASI score reduction (>3/72 PASI score at study onset and after 24 weeks and PASI score at study onset >PASI score after 24 weeks×1.3) PASI score progression (>3/72 PASI score at study onset and after 24 weeks and PASI score at study onset <PASI score after 24 weeks) Clinical appearance of PASI score progression (≤3 PASI score at study onset and >3 PASI score after 24 weeks) No significant change (>3/72 PASI score at study onset and after 24 weeks and PASI score at study onset×1.3 >PASI score after 24 weeks)
PASI change after 24 weeks (binary)	Binary	1, if PASI score improved (constantly low PASI score or PASI75/PASI90) 0, if PASI aggravated (minor PASI score reduction, clinical appearance of PASI score progression, and no significant change)

^a^NRS: numeric rating scale.

^b^HADS-A: Hospital Anxiety and Depression Scale-Anxiety.

^c^HADS-D: Hospital Anxiety and Depression Scale-Depression.

### Exploratory Data Analysis

With the secondary data set, our objective was to train and test ML models to identify potentially new, unknown associations between features. To do this, we used DataRobot’s AutoML technology [48-50]. The first step was to analyze the distribution of the data using DataRobot’s exploratory data analysis (EDA) tool. EDA is an important step because it helps to identify patterns, trends, and anomalies in the data before automatically building a model for selected features. Once the data have been imported, a data profiling report is generated, providing descriptive statistics and data quality metrics. The report reveals the number of rows and columns, missing values, data type, and distribution for the top 50 items of every feature. It also includes data quality metrics, such as mean, SD, median, minimum, and maximum. The process of feature engineering is used to improve the performance of predictive models. This involves creating new features or transforming existing raw features into different types, such as numeric, categorical, Boolean, and so on. Errors in the data, such as outliers, missing values, or duplicate rows, are identified and corrected. Details of the processing of missing values, called imputation, are provided in Multimedia Appendix 4.

After selecting a target feature that is to be predicted or analyzed, a second EDA is performed. For this study, the following targets were selected:

- Target 1.1: *therapy change at 24 weeks follow-up*
- Target 1.2: *therapy change prediction (only onset features)*
- Target 2: *PASI change after 24 weeks*
- Target 3: *BASDAI classification at study onset*

The numerical statistics initially calculated in the first EDA are recalculated in the second EDA. For each feature in the data set, the correlation between the feature and the target is independently ranked according to its importance. Features with high importance are included, whereas features with low importance are excluded. This results in so-called reduced feature lists. At this point, the majority of characteristics at 12 weeks’ follow-up were ranked as having low importance for the selected targets. This was due to the fact that 141 (45.6%) of the 309 patients had missing data set values at this follow-up time because in the second clinical trial the 12-week follow-up was performed either for patients with high musculoskeletal symptoms or for patients with PsA. By contrast, the 24-week follow-up was mandatory for study patients in both clinical trials. Therefore, modeling was performed with a reduced feature list for each target (Multimedia Appendices 5-8). Moreover, individual feature lists can also be created. Targeted analysis of important features can reduce error and increase accuracy. Most importantly, further data quality checks are performed to identify outliers, target leakage, and imputation leakage.

### Model Building, Validation, and Selection

Once the data are prepared and features engineered, DataRobot’s AutoML platform begins the process of model building. It uses an AutoML approach, which involves testing a wide range of models and selecting the best one for the given data set. The model selection process includes supervised learning techniques, such as regression or classification, depending on the target’s feature type. Such supervised ML algorithms include bagging, boosting, deep learning, random forests, frequency-severity methods, kernel-based methods, and generalized linear models. In addition, ensemble models, also known as blenders, can improve accuracy by combining the predictions of anywhere from 2 to 8 models; for example, an average blender averages each model’s prediction as its own [51].

At the outset of the model-building process, the data set is partitioned into a training set, a holdout set, and a validation set. The training set is used to build models, and the validation set is used to evaluate the performance of a model using data it has not seen before. The holdout partition set, by contrast, is not available during model building and can be used as an additional check against selection bias. The exact partitioning percentages for the 4 targets in our study are presented in Table 2.

**Table 2:** Partitioning percentages for the 4 targets.

		Values, n (%)
**Target 1.1 (n=237)**		
	Training	152 (64.1)
	Validation	38 (16)
	Holdout	47 (19.8)
**Target 1.2 (n=237)**		
	Training	152 (64.1)
	Validation	38 (16)
	Holdout	47 (19.8)
**Target 2 (n=236)**		
	Training	151 (64)
	Validation	38 (16.2)
	Holdout	47 (19.9)
**Target 3 (n=129)**		
	Training	82 (63.6)
	Validation	21 (16.3)
	Holdout	26 (20.2)

On each model, the AutoML platform automatically explores different configurations (hyperparameters) to optimize the model (Multimedia Appendix 9). This hyperparameter optimization includes methods such as grid search, random search, learning rate in neural networks, and early stopping [52]. Of note, early stopping is a method for determining the number of trees to use for a boosted trees model. The training data are split into a training set and a test set, and at each iteration the model is evaluated on the test set. The early stopping test set uses a 90:10 train-test split within the training data for a given model. As the early stopping test set is used for early stopping, it cannot be used for training. All ML models are ranked according to their scores on the platform’s leaderboard after automated modeling is complete. Scores are displayed for the validation and holdout partitions, while a third cross-validation (CV) score is also displayed, representing the average of 5 scores calculated on 5 different training and validation partitions (5-fold CV), with a remaining holdout partition. Provided score metrics are dependent on the supervised learning type: regression, binary, or multiclass classification; for example, regression analysis returns scores such as gamma deviation, mean absolute error, mean absolute percentage error, Poisson deviation, or R-squared. For binary classifications, Kolmogorov-Smirnov and maximum Matthews correlation coefficients (MCC) are displayed, while logarithmic loss (LogLoss) and area under the receiver operating characteristic curve (AUC) are displayed for both binary and multiclass classifications. Finally, root mean square error and Gini impurity are commonly used metrics for regression and classification modeling, respectively. Especially for binary classifications, a model is only suitable if there is a predictive threshold that can effectively distinguish between true positives and true negatives. In addition to the MCC, the *F*_1_-score provides an indication of this. This is a metric based on precision and recall. The higher the *F*_1_-score, the higher the positive predictive value and true positive rate in the respective data partitions. Its maximum is 1. For target 1.1, we chose the maximum MCC as the threshold, whereas for target 3, we chose the maximum *F*_1_-score as the threshold. However, for targets 1.2 and 2, we manually determined the optimal threshold, seeking greater accuracy by considering both *F*_1_-score and MCC value.

After selecting the best-performing model based on its AUC and LogLoss metrics within the CV partition, the AutoML platform provides the results of the feature impact analysis, which identifies the most important features in the model by calculating permutation importance and helps to understand how they contribute to the model’s outcome. Specifically, the feature impact analysis shows how much the error of a model would increase, based on a sample of the training data, if the values in a given column were shuffled while other columns were left unchanged. The AutoML platform then normalizes the results so that the value of the most important feature column comes first, and the other features that follow are normalized to it. Once selected, the model’s performance can be further optimized by identifying unimportant or redundant features, selecting the best feature combinations, and adjusting the hyperparameters. Finally, the platform provides detailed reports and visualizations, called blueprints, that show how each model performed on the data set (Multimedia Appendix 10). They include all preprocessing steps, modeling algorithms, and postprocessing steps that were performed during model development. In addition, a more detailed view of the performance metrics achieved during training is provided by the learning curves during holdout partition and further LogLoss values across all CV folds (Multimedia Appendix 11). Finally, to ensure the variability and stability of our models’ performance across different data splits, we reran all our models for validation, CV, and holdout splits 10 times with 10 different random seeds and plotted the medians of all reported metrics with 95% CIs (Multimedia Appendices 12-15). In this particular case, seeds are random number generators that are used to shuffle the data before they are split into training, validation, and holdout sets. Our study complies with the TRIPOD (Transparent Reporting of a Multivariable Prediction Model for Individual Prognosis or Diagnosis) guidelines (Multimedia Appendix 16).

### Software

Statistical analysis was performed with GraphPad Prism 9.5.1 (GraphPad Software) and AutoML analysis with DataRobot’s Automated Machine Learning product (version 5b1d33).

### Ethics Approval

This study (2021-895) and the 2 independent clinical trials (2017-655N-MA and 2020-515N-MA) used to generate the secondary data sets were reviewed and approved by the Medical Ethics Committee II of the Medical Faculty Mannheim, University of Heidelberg, Germany.

## Results

### Overview

In this study, we aimed to leverage ML models to explore predictive patient features for therapeutic response and disease progression in PsV or PsA. We focused on 3 main research questions (RQs):

- RQ1: Why was the psoriasis therapy changed during the study?
- RQ2: What influenced the progression of skin lesions during the 6-month observation period?
- RQ3: What factors were associated with an initial abnormal BASDAI score?

To answer these RQs, we used the secondary data set to build ML models. A key aspect of AutoML is the selection of the target features, that is, the variables of interest that the ML model will attempt to classify or predict. Therefore, our selected target variables were (1) *therapy change at 24 weeks follow-up* and *therapy change prediction*, (2) *PASI score change after 24 weeks*, and (3) BASDAI classification at onset.

The most accurate models were selected based on their AUC and LogLoss holdout scores because classification AutoML classification analysis was used for all targets (Table 3). Two separate models were selected for the *therapy change* targets: one to understand how different study onset and follow-up features influence the target over the course of the clinical trials and the other to predict therapy change using onset features only. For *therapy change at 24 week follow-up* with all follow-up features included, the selected model was the *eXtreme Gradient Boosted (XGBoost) Trees Classifier with Early Stopping-Forest (10x)*. For the *therapy change prediction* target with only baseline features, an average blender of a *gradient boosted trees classifier*, an *ExtraTrees classifier (Gini)*, and a *Eureqa generalized additive model classifier (1000 generations)* was the most accurate model. Another average blender incorporating 3 models (a *random forest classifier [Gini]*, an XGBoost *trees classifier [learning rate=0.01]*, and a *Eureqa classifier [default search 3000 generations]*) was selected for *PASI change after 24 weeks*, while an average blender of a *Eureqa generalized additive model classifier (40 generations)*, an *XGBoost trees classifier with early stopping*, and a *dropout additive regression trees classifier (15 leaves)* was selected for the target variable *BASDAI classification at onset*.

**Table 3:** Model performance metrics for the holdout partition are shown. The best model was selected based on area under the receiver operating characteristic curve (AUC) and logarithmic loss (LogLoss) metrics from cross-validation. The Compared models column lists the total number of models built and trained for each target. Decision points were set using the receiver operating characteristic curve at the highest F1-score or maximum Matthews correlation coefficients (MCC). Even if a new decision point in the holdout partition seemed better, the cross-validation value was retained. F1-scores and MCC values were obtained by applying cross-validation thresholds to the holdout data.

Target	Compared models	Selected model	AUC	LogLoss	RMSE^a^	*F*_1_-score	MCC
Target 1.1: therapy change at 24 weeks follow-up	303	eXtreme Gradient Boosted Trees Classifier with Early Stopping-Forest (10x)	0.9078	0.3955	0.3583	0.8182	0.6911
Target 1.2: therapy change prediction (only onset features)	145	Average blender incorporating 3 models: gradient boosted trees classifier, ExtraTrees classifier (Gini), and Eureqa generalized additive model classifier (1000 generations)	0.8750	0.4603	0.3815	0.7917	0.5743
Target 2: PASI^b^ change after 24 weeks	328	Average blender incorporating 3 models: random forest classifier (Gini), extreme gradient boosted trees classifier (learning rate=0.01), and Eureqa classifier (default search 3000 generations)	0.9241	0.4498	0.3787	0.8966	0.7439
Target 3: BASDAI^c^ classification at onset	140	Average blender incorporating 3 models: Eureqa generalized additive model classifier (40 generations), extreme gradient boosted trees classifier with early stopping, and dropout additive regression trees classifier (15 leaves)	0.8274	0.5037	0.4099	0.8000	0.5367

^a^RMSE: root mean square error.

^b^PASI: Psoriasis Area and Severity Index.

^c^BASDAI: Bath Ankylosing Spondylitis Disease Activity Index.

### Target 1.1: Therapy Change at 24 Weeks Follow-Up

By choosing a change in therapy as a target, we wanted to analyze the relationship between an impending change in the patient’s systemic therapy and the patient’s baseline data, including the patient’s previous systemic and topical therapies as assessed at enrollment as well as disease activity markers and scores over the previous 6 months. Therefore, baseline patient data were narrowed down to essential clinical features, such as age, BMI, occupation, previous diseases, and lifestyle factors (eg, smoking, alcohol consumption, and exercise), which represent easily and quickly collected data from all patients in daily clinical practice. Disease activity scores and markers were questionnaire scores (eg, the HADS, DLQI, and Classification Criteria for Psoriatic Arthritis [CASPAR]) or physician-reported severity of skin involvement (PASI scores) over 24 weeks. The list of features used and reduced by the AutoML platform for this target is given in Multimedia Appendix 5.

**Figure 1:**
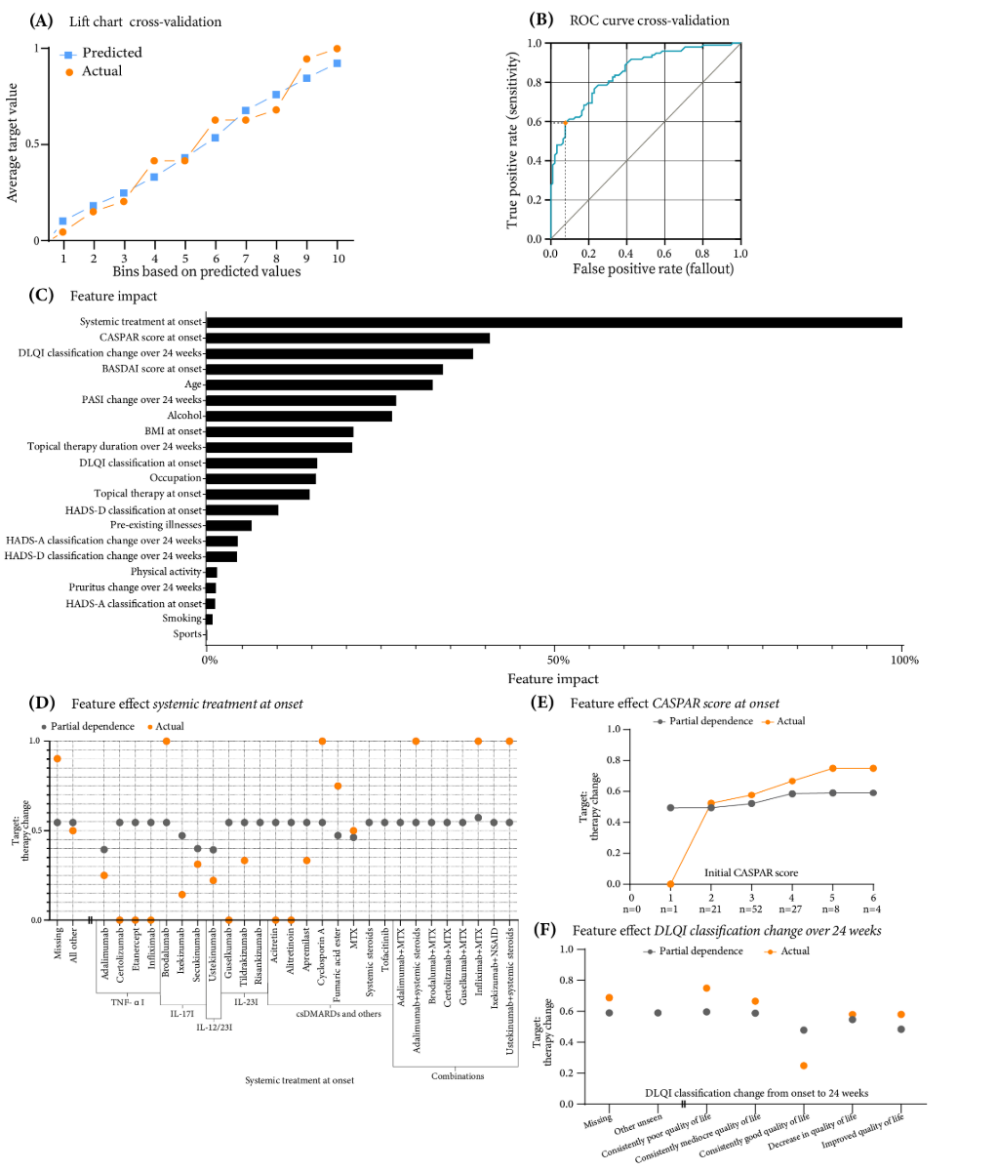
Analysis of therapy change outcomes in psoriasis vulgaris and psoriatic arthritis using an eXtreme Gradient Boosted Trees Classifier with Early Stopping-Forest (10x) model. The analysis was conducted to assess therapy modification needs at a 24-week follow-up. (A) Lift chart: compares actual and predicted values, showing predictive consistency through cross-validation. (B) Receiver operating characteristic (ROC) curve: displays the decision threshold set at 0.6998 during cross-validation. (C) Feature impact rankings: normalizes and ranks clinical and demographic factors influencing therapy change. (D) Feature effect of systemic treatment at onset on therapy change at a 24-week follow-up. (E) Feature effect of CASPAR score at onset on therapy change at a 24-week follow-up. (F) Feature effect of Dermatology Life Quality Index (DLQI) classification change over 24 weeks on therapy change at a 24-week follow-up. BASDAI: Bath Ankylosing Spondylitis Disease Activity Index; csDMARD: conventional synthetic disease-modifying antirheumatic drug; HADS-A: Hospital Anxiety and Depression Scale-Anxiety; HADS-D: Hospital Anxiety and Depression Scale-Depression; IL-12I: interleukin-12 inhibitor; IL-17I: interleukin-17 inhibitor; IL-23I: interleukin-23 inhibitor; MTX: methotrexate; NSAID: nonsteroidal anti-inflammatory drug; PASI: Psoriasis Area and Severity Index; TNF-αI: tumor necrosis factor alpha inhibitor.

During the 6-month observation period, of all patients included in the training partition by the AutoML platform, systemic therapy was changed in 51.5% (122/237) and remained unchanged in 48.5% (115/237). Of note, 72 (23.3%) of the 309 patients were excluded during EDA due to missing values in the target feature. Therapy changes were made either at 12 weeks (midpoint of the study) or at the end of the study (24 weeks). A total of 303 binary classification models were trained, with the XGBoost trees classifier with early stopping emerging as the selected model based on its performance metrics, which included AUC values of 0.7729 (validation), 0.8536 (CV), and 0.9078 (holdout) and LogLoss values of 0.5782 (validation), 0.4854 (CV), and 0.3955 (holdout). The model lift chart for the CV partition showed an almost converging trend between the actual and predicted values (Figure 1A). A threshold of 0.6998 was set to discriminate between positive and negative predictions, giving an *F*_1_-score of 0.7117, sensitivity of 0.5918, and precision of 0.8923 in CV (Figure 1B) and an *F*_1_-score of 0.8182, sensitivity of 0.7200, and precision of 0.9474 in the holdout (Figure S1 in Multimedia Appendix 17). The primary predictor of therapy change was the systemic therapeutic agent prescribed to the patient at onset, with a normalized importance of 100% (Figure 1C). This was followed by the initial CASPAR score (40.89%) and changes in QoL during the trial (38.49%).

Given that initial systemic therapy had the strongest feature importance in this model, a detailed look at its effect revealed the impact of its individual data values on the model’s predictions. When partial dependence (PD) was considered, it was found that of the 27 different systemic therapies, only 7 (26%) differed from the other systemic therapies (Figure 1D). PD was first proposed to address the difficulty of interpreting more complex ML models [53]. It shows how, with all other features held constant except for the feature of interest, the value of this feature affects the model’s prediction. Specifically, the AutoML platform keeps the values of all features constant except for the one being considered. The platform then reassigns the value of the feature of interest to each possible value and calculates the average prediction at each of these settings. Therefore, the PD’s data points represent the marginal effect of a feature on the target variable; for example, while 20 therapies had a PD of 0.5458, a combination of infliximab and MTX had the only slightly higher PD (0.5729) and was therefore more favorable for switching therapies during treatment. Fumaric acid esters (0.4733), ixekizumab (0.4723), MTX (0.4630), secukinumab (0.4000), adalimumab (0.3947), and ustekinumab (0.3933) had clearly lower PDs and were therefore more favorable for no change in therapy. However, an examination of the actual values of the training partition revealed that other systemic agents with unremarkable PD values were changed. Specifically, therapies such as brodalumab, cyclosporine A, and all recorded combinations of systemic drug therapies, including adalimumab and MTX, infliximab and MTX, and ustekinumab and systemic steroids, were not maintained. Of note, 90% (37/41) of the patients without initial systemic therapy started systemic therapy during the study. Finally, despite the low PD values in the training partition, fumaric acid esters were discontinued more frequently (6/8, 75%).

Next, higher initial CASPAR scores resulted in a higher probability of treatment change within the actual values of the training partition as well as according to PD values. For a CASPAR score of 2 (PD=0.4942), the change was 52% (11/21); for 3 (PD=0.5217), the change was 58% (30/52); for 4 (PD=0.5844), the change was 66% (18/27); for 5 (PD=0.5900), the change was 75% (6/8); and for 6 (PD=0.5900), the change was 75% (3/4; Figure 1E). The change in DLQI classification over 24 weeks showed a similar distribution of actual training partition and PD values (Figure 1F). Consistently poor QoL led to a change in therapy in most cases (PD=0.5962; 9/12, 75%). When QoL was consistently good, the likelihood of changing therapy was also the lowest (PD=0.4804; 10/40, 25%). Finally, a consistently moderate score according to the DLQI led to a change almost as often as a poor QoL (PD=0.5896; 2/3, 67%).

### Target 1.2: Therapy Change Prediction (Only Onset Features)

The previously constructed model was used to explore the potential influence of various patient lifestyle factors, clinical characteristics, medical scores, and past medical history on changes in therapeutic interventions during clinical trials, using both baseline and follow-up data from the secondary data set. However, for subsequent predictive applications, it is crucial to exclude all follow-up data except for the target variable itself to avoid the potential influence of future data points on the target, also known as target leakage. Thus, these points should be omitted to produce more accurate predictive models.

Therefore, we were interested in performing a second AutoML analysis for the *therapy change* target, using only baseline characteristics collected at study entry. The aim was to determine whether a highly accurate model could be selected that could hypothetically predict therapy changes in patients with PsA and PsV being treated in our dermatology department. Baseline data used in this analysis included age, BMI, occupation, previous medical conditions, and current medication, as well as lifestyle factors such as exercise, smoking, and alcohol consumption. The range of features used, as reduced by the AutoML platform for this target, is shown in Multimedia Appendix 6.

**Figure 2:**
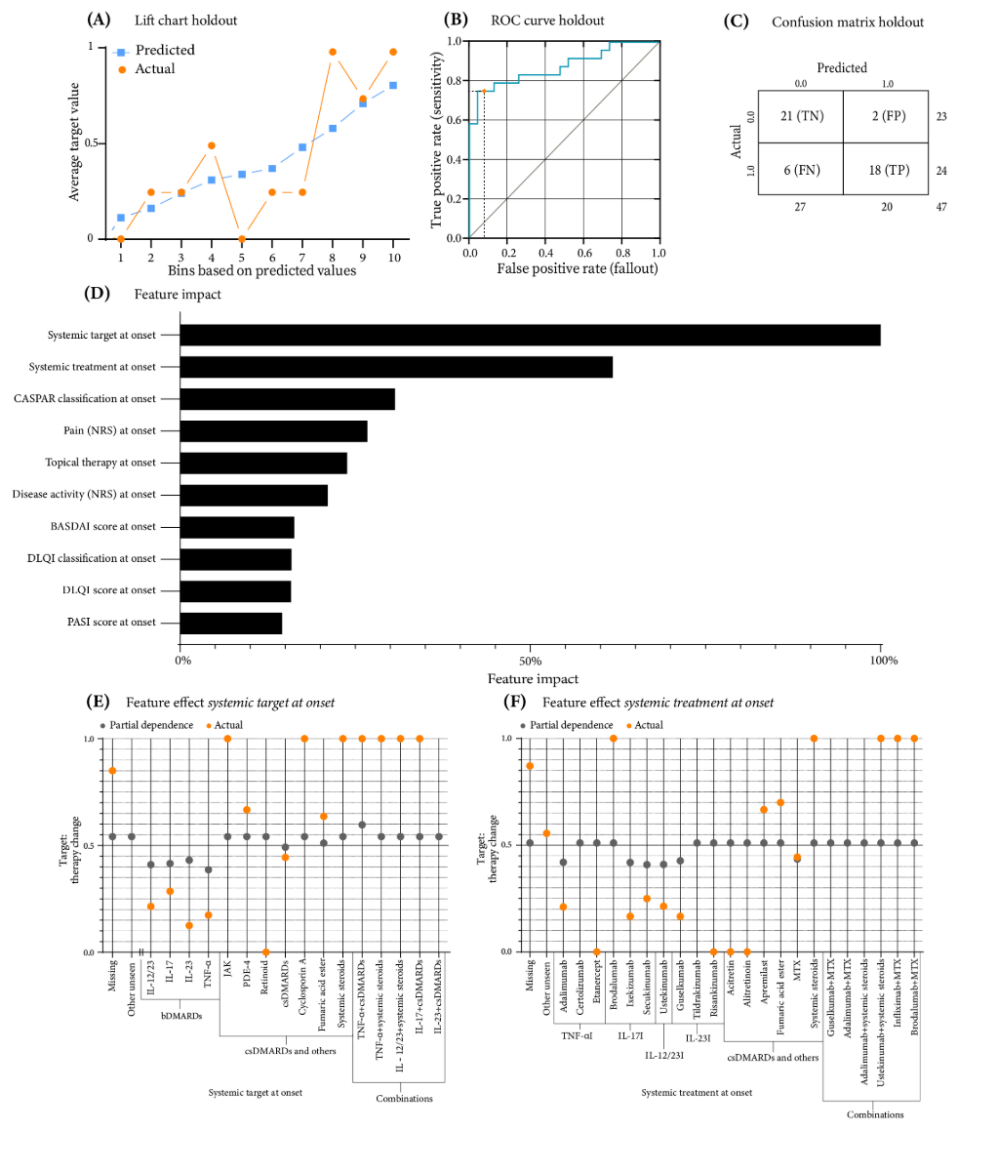
Evaluation of the predictive accuracy of therapy change predictions in psoriasis vulgaris and psoriatic arthritis using an AVG average Blender model. (A) Lift chart: contrasts actual and predicted values for the holdout partition. (B) Receiver operating characteristic (ROC) curve: displays the decision threshold set at 0.564 for the holdout partition. (C) Confusion matrix: delineates true negatives (TNs), false positives (FPs), false negatives (FNs), and true positives (TPs) at the chosen threshold. (D) Feature impact rankings: normalizes and ranks the top 10 onset features influencing therapy change. (E) Feature effect of systemic target at onset on therapy change at a 24-week follow-up. (F) Feature effect of systemic treatment at onset on therapy change at a 24-week follow-up. BASDAI: Bath Ankylosing Spondylitis Disease Activity Index; bDMARD: biologic disease-modifying antirheumatic drug; CASPAR: Classification Criteria for Psoriatic Arthritis; csDMARD: conventional synthetic disease-modifying antirheumatic drug; DLQI: Dermatology Life Quality Index; IL-12: interleukin-12; IL-12I: interleukin-12 inhibitor; IL-17: interleukin-17; IL-17I: interleukin-17 inhibitor; IL-23: interleukin-23; IL-23I: interleukin-23 inhibitor; JAK: Janus kinase; MTX: methotrexate; NRS: numeric rating scale; PASI: Psoriasis Area and Severity Index; PDE-4: phosphodiesterase 4; TNF-α: tumor necrosis factor alpha; TNF-αI: tumor necrosis factor alpha inhibitor.

A total of 133 models were trained using binary classification analysis. The model selected was an average blender incorporating a *gradient boosted trees classifier*, an *ExtraTrees classifier (Gini)*, and a *Eureqa generalized additive model classifier*. The performance of the model was measured by the AUC, which reached values of 0.9390 (validation), 0.8590 (CV), and 0.8750 (holdout). The LogLoss values were 0.4050 (validation), 0.4970 (CV), and 0.4600 (holdout). Although the lift chart for the holdout partition showed more scatter than the previous model for the identical target with the inclusion of follow-up data, its upward trajectory provided evidence for its accuracy (Figure 2A). The threshold for classifying a prediction as positive or negative was set at 0.5463 after CV, giving an *F*_1_-score of 0.8200, a sensitivity of 0.7500, and a precision of 0.9000 during the holdout partition (Figure 2B). A confusion matrix was then developed to provide a detailed summary of the prediction results (Figure 2C). The matrix showed that the model correctly identified 21 true negatives and 18 true positives. However, it also incorrectly predicted therapy changes in 2 cases (false positives) and missed 6 cases where therapy changes were required (false negatives).

Given these metrics, the model seemed to be effective in predicting therapy changes in patients with PsV. Consequently, we further explored the impact of the model features (Figure 2D). Interestingly, patients’ systemic therapy emerged as a significant factor in predicting therapy changes. The feature with the highest impact was *systemic target at onset*, a newly classified multiclass feature (Multimedia Appendix 3), followed by *systemic treatment at onset*, which was the most influential feature in the previous model. Other baseline features that influenced the target in our chosen model were *CASPAR classification at onset*, *pain (NRS [numeric rating scale]) at onset*, and *topical therapy at onset*. Interestingly, the effects of systemic target features indicated that TNF-α, IL-17, IL-23, and IL-12/IL-23 inhibitors were less likely to require a change in therapy compared to other classes of systemic therapy (Figure 2E). Conversely, the combination of TNF-α inhibitors with csDMARDs was slightly more likely to require a change in therapy. An examination of the effect of *systemic treatment at onset* confirmed these observations, indicating that adalimumab, ixekizumab, secukinumab, ustekinumab, and guselkumab were less likely to influence a positive change in systemic therapy (Figure 2F). Interestingly, MTX was also notably less likely to be changed, as confirmed by both PD and actual values.

To illustrate how our chosen model could be used in everyday clinical practice, we considered 2 hypothetical patients for whom the ML model calculated the likelihood of changing therapy after 24 weeks (Multimedia Appendix 18). The first patient was a woman aged 47 years with both PsV and PsA with ongoing systemic therapy with adalimumab (a TNF-α inhibitor), topical therapy, a CASPAR score of 3, a BASDAI score of 4, a PASI score of 10, moderate pain (an NRS score of 4), moderate disease activity (an NRS score of 4), and mild pruritus (an NRS score of 3). Her assessed medical scores showed moderate impairment of QoL according to the DLQI, as well as marked anxiety and borderline depression according to the HADS. Her calculated therapy change prediction score was 0.5097, which was below the established threshold (Multimedia Appendix 18). This implied a negative prediction with a negative predictive value of 0.79. However, if another prediction is made, this time for a second patient, whose clinical characteristics resemble those of the first patient, with the only difference being that she is receiving a combination of systemic steroids and adalimumab (a TNF-α inhibitor) instead of adalimumab as monotherapy, the prediction of 0.6758 is above the threshold and therefore positive, with a positive predictive value of 0.95.

### Target 2: PASI Change After 24 Weeks

**Figure 3:**
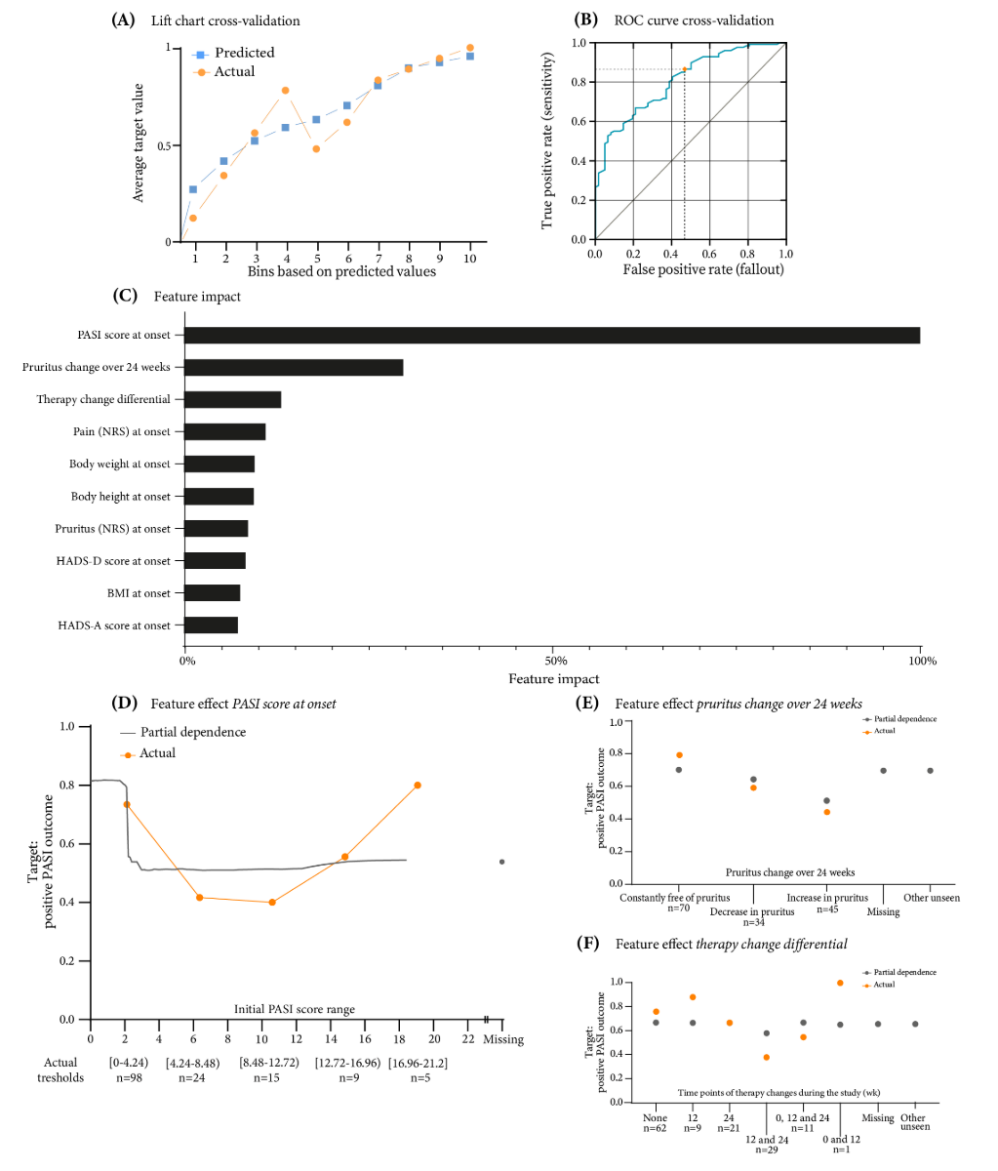
Evaluation of Psoriasis Area and Severity Index (PASI) score change outcomes after 24 weeks using an average blender model. (A) Lift chart: compares actual and predicted values through cross-validation. (B) Receiver operating characteristic (ROC) curve: displays the decision threshold set at 0.5485 during cross-validation. (C) Feature impact rankings: normalizes and ranks the top 10 features influencing PASI score change after 24 weeks. (D) Feature effect of baseline PASI score on PASI change at a 24-week follow-up. (E) Feature effect of pruritus change over 24 weeks on PASI score change. (F) Feature effect of therapy change differential on PASI score change at a 24-week follow-up. HADS-A: Hospital Anxiety and Depression Scale-Anxiety; HADS-D: Hospital Anxiety and Depression Scale-Depression; NRS: numeric rating scale.

By evaluating the feature *PASI change after 24 weeks* (binary), we aimed to assess treatment response and disease progression over a clinically meaningful period, as well as provide valuable insight into the long-term efficacy of therapeutic interventions for PsV. The third model selected in this study analyzed the progression of skin involvement in PsV by incorporating all available features, including several binary features newly created from multiclass features such as obesity, depression, hypertension, and TNF-α–targeting therapy (Multimedia Appendix 3). The list of features used and reduced by the AutoML platform for this target is given in Multimedia Appendix 7. PASI measurements were taken at baseline and at follow-up and transformed into a new feature. This new binary classification of PASI score change at follow-up is shown in Table 1. Of the 236 patients in the training partition, 154 (65%) had a positive PASI outcome, and 82 (35%) had a negative PASI outcome. A total of 328 models were trained during the automation process. Once again, the selected model’s lift chart showed a closeness of the predicted line to the actual line and an upward trajectory of its curves, indicating accuracy (Figure 3A). The metric considered for the selected model was the AUC (validation: 0.8500, CV: 0.8468, and holdout: 0.9241). The threshold for determining positive or negative prediction was set at 0.5485 (Figure 3B). This resulted in an *F*_1_-score of 0.8271, sensitivity of 0.8661, and precision of 0.7914 in CV and an *F*_1_-score of 0.8966, sensitivity of 0.9630, and precision of 0.8387 in holdout (Figure S2 in Multimedia Appendix 17). Holdout patients were divided into a group with an 84% chance of a positive outcome (0.8387 precision or positive predictive value) and a group with a 94% chance of a negative outcome (0.9375 negative predictive value).

The primary predictor of PASI score progression was found to be the initial PASI score (Figure 3C; Multimedia Appendix 19). Its feature impact was normalized to 100%. It was followed by *change in pruritus at follow-up* (30.26%) and *therapy change differential* (whether, how often, and when therapy was changed during the study; 13.47%). Other impactful features included pain at onset, body weight, body height, pruritus at baseline, HADS depression score at baseline, and BMI, but their influence on the target was minor. A closer look at the effect of the individual influential feature values shows that, according to PD, a PASI score of <2.12 was most likely to lead to an improvement in the PASI score after 24 weeks (PD≥0.7930; Figure 3D). An initial PASI score of ≥2.12 was less likely to lead to an improvement, regardless of the exact score. In fact, PD fell to a low plateau from a PASI score of 3, with a minimum of 3.46 (PD=0.5093). Changes in pruritus during the observation period also influenced PASI score progression. A positive PASI outcome was most likely when patients consistently reported no pruritus (PD=0.6940; 55/77, 79%; Figure 3E). When there were changes in pruritus, a decrease (PD=0.6640; 20/34, 59%) was more likely to be associated with a positive PASI outcome than an increase (PD=0.5410; 20/45, 44%). While therapy change was the third most influential feature in the selected model, the PD of the feature did not reveal striking differences between its classes and their influence on the target, with only therapy change at both 12 and 24 weeks having a slightly negative effect on a positive PASI outcome (Figure 3F).

### Target 3: BASDAI Classification at Onset

The final aim of the study was to investigate and analyze the applicability of the BASDAI score, used primarily in ankylosing spondylitis, to assess disease activity in patients with PsV and specifically those with PsA. Patients were stratified into 2 groups based on their initial BASDAI scores: inconspicuous (BASDAI score of ≤3; 68/129, 52.7%) and conspicuous (BASDAI score of ≥4; 61/129, 47.3%; Multimedia Appendix 3). A BASDAI score of ≥4 indicates ineffective therapy in patients with various types of ankylosing spondylitis, including PsA, requiring the initiation or modification of systemic therapy. The list of features used by the AutoML platform to analyze this target can be found in Multimedia Appendix 8.

**Figure 4:**
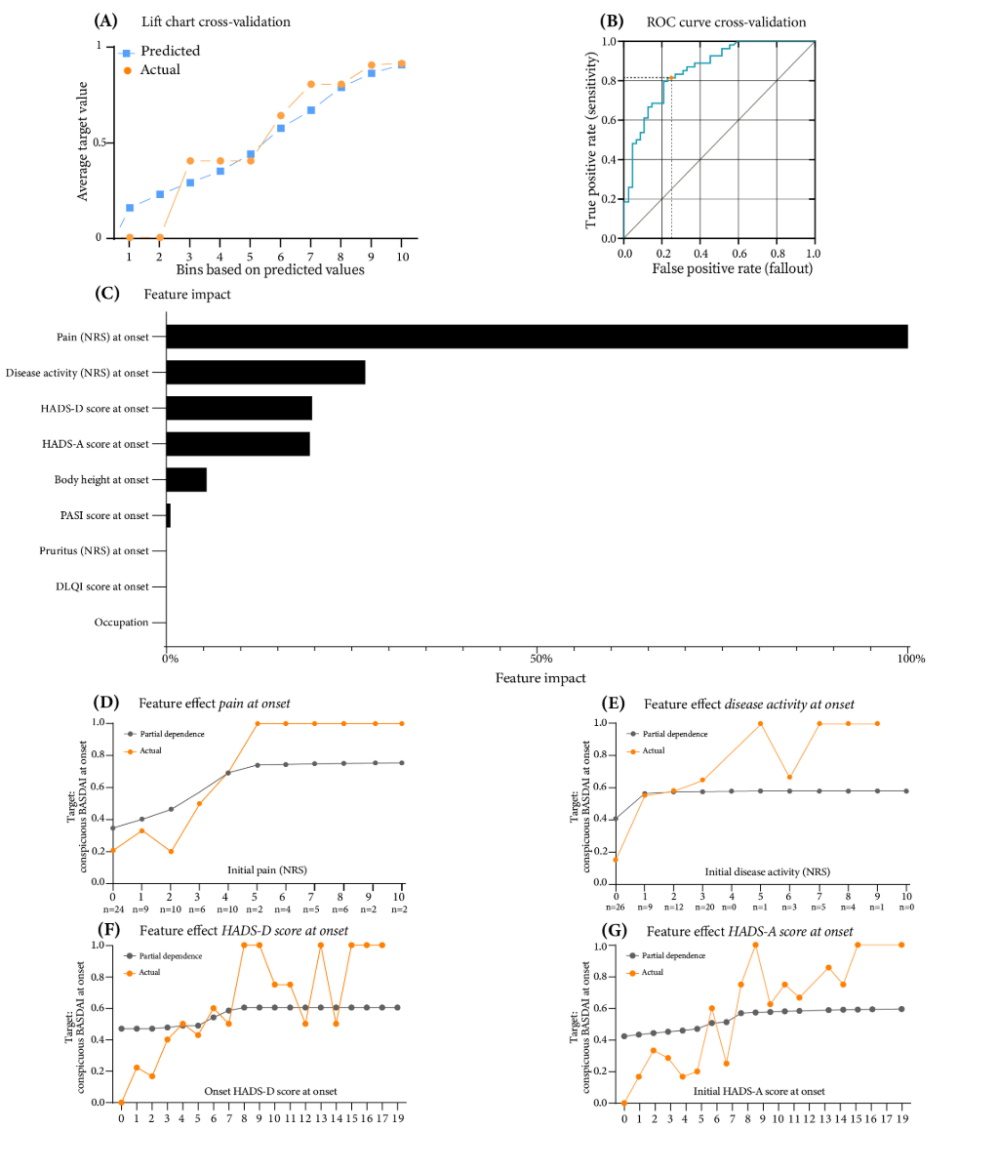
Evaluation of Bath Ankylosing Spondylitis Disease Activity Index (BASDAI) classification at onset using an average blender model. (A) Lift chart: compares actual and predicted BASDAI classifications during cross-validation. (B) Receiver operating characteristic (ROC) curve: displays the decision threshold set at 0.4471 during cross-validation. (C) Feature impact rankings: normalizes and ranks the top features influencing BASDAI classification. (D) Feature effect of pain at onset on BASDAI classification. (E) Feature effect of disease activity at onset on BASDAI classification. (F) Feature effect of Hospital Anxiety and Depression Scale-Depression (HADS-D) score at onset on BASDAI classification. (G) Feature effect of Hospital Anxiety and Depression Scale-Anxiety (HADS-A) score at onset on BASDAI classification. DLQI: Dermatology Life Quality Index; NRS: numeric rating scale; PASI: Psoriasis Area and Severity Index.

A total of 140 binary classification models were trained, with an average blender of a *Eureqa generalized additive model classifier*, an *XGBoost trees classifier with early stopping*, and a *dropout additive regression trees classifier* selected as the optimal model, with AUC values of 0.8273 (validation), 0.8618 (CV), and 0.8274 (holdout). Despite some scatter between the predicted and actual values, the lift charts show a positive trend in the curves (Figure 4A). Setting the decision threshold at 0.4471 gave an *F*_1_-score of 0.8000, sensitivity of 0.8148, and precision of 0.7857 in CV (Figure 4B), while the holdout analysis gave an *F*_1_-score of 0.8000, sensitivity of 0.8571, and precision of 0.7500 (Figure S3 in Multimedia Appendix 17).

For this target, the most influential feature was initial pain as assessed by the normalized NRS, which accounted for 100% of the impact (Figure 4C). Initial disease activity measured using the NRS contributed 27.11% of the impact, while the HADS depression and anxiety scores at baseline had an impact of 19.92% and 19.64%, respectively. Other relevant impactful features included body height, PASI score, and pruritus at onset. The effect of the feature initial pain showed that the PD value rose almost linearly with increasing values from an NRS score of 0/10 (PD=0.3490) to an NRS score of 5/10 (PD=0.7420) and remained at >0.7400 up to an NRS score of 10/10 (Figure 4D). The actual values of the training data showed that an initial NRS pain score of >3 was almost 4 times more likely to lead to a remarkable BASDAI score than an NRS pain score of ≤3 (>3: 28/31, 90% vs ≤3: 13/49, 27%). In addition, an NRS score of ≥5/10 resulted in a conspicuous BASDAI score in all cases (21/21, 100%). If the initial NRS disease activity score was 0/10, a conspicuous BASDAI score was unlikely (PD=0.4090; 4/26, 15%); however, any NRS disease activity score of >0 strongly increased the likelihood of a conspicuous BASDAI score (PD=0.5500-0.6000; Figure 4E).

In addition, psychological distress, more precisely depressive and anxious mood symptoms according to the HADS, had a strong influence on BASDAI outcomes. The effect of the feature *HADS-D score at onset* showed that values between 0 and 5 were favorable for an inconspicuous BASDAI score, and values of >8 were likely for an abnormal BASDAI score (Figure 4F). A similar pattern was seen in the PD of the corresponding effect of the feature *HADS-A score at onset* (Figure 4G). As the HADS-A score increased, an abnormal BASDAI score was more likely. It is interesting to note that the HADS-A score of 7 represented a turning point for the PDs of both psychological scores.

## Discussion

### Principal Findings

In this study, we sought to use AutoML to efficiently explore complex relationships within clinical trial data sets of patients with PsV and PsA. The implementation of AutoML in this context allowed us to better understand the dynamic interplay between treatment changes and disease course. As targets, we chose changes in systemic therapy, PASI score change after 24 weeks, and BASDAI classification at onset because they represent critical aspects or parameters when it comes to measuring and understanding disease progression and, in parallel, treatment efficacy in both plaque psoriasis and PsA. Key predictors included initial treatment choice, baseline clinical criteria, changes in QoL, initial PASI scores, changes in pruritus, and psychological factors.

Analyzing a change in systemic therapy can provide critical insight into symptom management or overall impact on QoL because it has been shown that between 40% and 50% of patients with moderate to severe psoriasis often report dissatisfaction with treatment [54]. In this regard, approximately half of the patients (79/147, 53.7%) in our evaluated training partition had changes in their systemic therapy. We selected a highly accurate model, the *XGBoost classifier with early stopping*, which performed exceptionally well based on both its metric scores and its lift charts, demonstrating an almost converging trend between actual and predicted values. XGBoost is an open-source implementation of the gradient boosted trees algorithm, well-known for its prediction power with its concept of early stopping being integrated to improve the model’s efficiency [55,56]. Although XGBoost is a widely used ML algorithm that has been applied in a variety of medical settings, such as predicting missing values in patients’ laboratory test results [57] or predicting heart conditions [58], we present here for the first time the specific use of XGBoost with early stopping on a medical data set. With this model, we were able to identify the initial systemic therapeutic agent, the initial CASPAR score, and changes in QoL as important predictors of therapy change in patients with PsV and PsA. Other influential features included BASDAI score at onset, age, PASI score change up to 24 weeks, alcohol consumption, BMI at onset, or topical therapy duration over 24 weeks. We focused on the effects of the 3 most impactful features. Our ML model indicated that the combination of infliximab and MTX was slightly more likely to be switched during treatment, while the monotherapies of fumaric acid esters, ixekizumab, MTX, secukinumab, adalimumab, and ustekinumab (in this particular order) were more likely not to be altered. In contrast to the unlikelihood of infliximab plus MTX being maintained as a long-term therapeutic option, studies of this combination have shown that it is effective in the treatment of several inflammatory diseases, including rheumatoid arthritis [59,60], ankylosing spondylitis [61], and Crohn disease [62]. However, several questions remain, despite the overall effectiveness of the combination. First, this efficacy has not been documented for PsA and PsV with regard to the corresponding monotherapies. Second, the optimal duration of this combination therapy and its effectiveness in nonnaive patients is not completely clear [62]. Furthermore, there are cases where combination therapy is not always superior to monotherapy. A randomized controlled trial in children with moderate to severe Crohn disease compared infliximab monotherapy with combination therapy for maintenance of clinical remission, and the results showed no difference between infliximab monotherapy and its combination with an immunomodulatory agent such as azathioprine or MTX [63]. This partly reflects our observation that infliximab and MTX alone had a better chance of continuation than their combination. Of note, individual responses may vary considerably because approved PsV and PsA therapies have different mechanisms of action and are therefore designed to treat different aspects of these diseases. To further illustrate this contention, while our chosen model showed a lower likelihood of switching MTX than ixekizumab, multicenter randomized trials have shown superiority in PsV therapy efficacy for ixekizumab compared to fumaric acid esters or MTX [64,65]. Similarly, patients with PsA who were switched from secukinumab to ixekizumab showed improvements 12 weeks after this change in therapy [66]. Conversely, we observed a higher likelihood of maintaining secukinumab than ixekizumab. Consistent with our observations, a randomized controlled trial demonstrated superiority of risankizumab over fumaric acid esters, whereas fumaric acid esters were more likely to be maintained compared to risankizumab, as shown by our ML model [67]. At this point, it is important to emphasize that therapeutic efficacy or superiority should not be equated with the likelihood that a therapy will be maintained or changed. Apart from therapeutic success, there are other factors that play a role in maintaining a particular therapy, such as patients’ own preferences, the cost of treatment, or adherence to lines of therapy. Therefore, clinicians and patients should discuss all these other contextual factors when choosing the best treatment approach.

In this respect, the use of the initial CASPAR score and changes in QoL could also potentially open up avenues for more tailored treatment approaches in the management of PsV and PsA. The CASPAR score was primarily developed for clinical trials rather than for diagnosis in everyday practice because PsA is often difficult to diagnose due to overlapping symptoms with other types of arthritis. The CASPAR scores are known to be highly sensitive and specific in classifying patients with long-standing PsA but are also considered valid as inclusion criteria for clinical trials in early PsA [68]. However, recent developments have shown that the integration of ultrasound into the CASPAR criteria can improve diagnostic performance [69]. Furthermore, some clinicians advocate the use of the CASPAR score due to its simplicity, ease, and speed of application in daily clinical practice [70]. This diagnostic scoring tool may also help to ensure that patients receive appropriate and timely treatment for their condition because we observed that higher initial CASPAR scores were associated with a higher likelihood of treatment change at 24 weeks. With regard to QoL, however, there is indeed evidence to show how it can influence changes in therapy [71]. QoL is a complex and important concept in health-related outcomes that can also be used to guide therapeutic strategies in different areas of medicine; for example, in renal failure, QoL can significantly influence the preferred mode of dialysis treatment, which directly impacts therapy change [72]. In oncology, the patient’s QoL is often an important factor in determining the course of treatment, particularly in the case of terminal illness [73]. For patients with dermatological conditions, PsV is the most appropriate example of how a skin condition affects QoL to guide treatment decisions [74]. According to the first European consensus on PsV, moderate to severe psoriasis was defined, among other criteria, as having a DLQI score of >10 [75], a fact that still advocates the initiation of systemic therapy in the European guidelines for the treatment of psoriasis [13]. Furthermore, achieving a DLQI score of >5 on treatment was considered a treatment failure and required a change in treatment regimen [75]. In patients with rheumatologic conditions, QoL has not yet been established as a definitive factor influencing treatment decisions. However, there are reports that support tailored treatment decisions that take QoL into account; for example, a study of patients’ health-related QoL showed significant differences across different rheumatic diseases, which may influence therapeutic decisions by both patients and their physicians [76]. The development of a Psoriatic Arthritis Quality of Life tool has also been reported, showing a good correlation with mostly MTX-treated outcomes, but further placebo-controlled or biologic treatment trials are lacking [77,78]. Given that we observed with our selected model that not only consistently poor but also consistently moderate QoL in patients with PsA and PsV was likely to lead to a change in therapy, we believe that in addition to incorporating QoL scores into ML prediction models, health care providers should generally consider QoL as a critical factor when making treatment decisions for these 2 chronic diseases.

To provide a directly understandable example of ML therapy change prediction implementable with our data set, we trained a new set of models based only on our study onset information. While the previously selected model provided new insights into the relationship between treatment change and disease activity trends, to make predictions with our data set from study entry for the next 24 weeks, we had to exclude all information that was unknown at study entry, also known as data leakage. Therefore, our focus this time was to explore the relationship between an upcoming change in the patient’s systemic therapy and their baseline data, including previous systemic and topical therapies and disease activity markers. This approach is also clinically relevant because there is evidence that a patient’s baseline status can significantly influence their treatment response [79]. Despite the reduced initial input information, the ability to train many accurate models exceeded our expectations. From the 145 trained models, we selected an average blend of 3 models, namely a *gradient boosted trees classifier*, an *ExtraTrees classifier (Gini)*, and a *Eureqa generalized additive model classifier*. ML average blender models are also referred to as ensemble models, which combine the predictive capabilities of several models to achieve better accuracy and robustness [80]. The *gradient boosted trees classifier* is an ML technique used for both regression and classification tasks. It constructs an ensemble of weak prediction models, typically decision trees, in a stepwise fashion to enhance predictive accuracy [81]. At each stage, the gradient boosted trees classifier adds a new decision tree that predicts the residuals of the current strong mode. The *ExtraTrees classifier* implements a meta-estimator that fits a number of randomized decision trees (extra trees) to different subsamples of the data set and uses averaging to improve prediction accuracy and control overfitting [56]. Finally, generalized additive models are a class of models that generalize linear models by allowing for nonlinear functions of each of the variables [82], while Eureqa is a symbolic regression that uses evolutionary algorithms to derive mathematical equations from large data sets in their simplest form [83].

Using this model, we observed that biologic disease-modifying antirheumatic drugs were less likely to be switched compared to the majority of other psoriasis therapies, whereas TNF-α inhibitors combined with csDMARDs had a higher switching rate. Long-term persistence of psoriasis systemic therapies has been reported mainly for biologics in patients with PsV and PsA [84,85], but a study assessing treatment patterns, persistence, and compliance in newly diagnosed patients with PsV and PsA from 2012 to 2018 in Stockholm, Sweden, found 5-year persistence rates of 32%, 45%, and 19% for MTX, biologics, and other systemic treatments, respectively [86]. When considering the combination of TNF-α inhibitors with csDMARDs, the literature shows the opposite of our observation, stating that this therapy combination does not lead to a significant increase in discontinuation rates compared to TNF-α inhibitor monotherapy [87]. Looking at the individual agents, we observed that adalimumab, ixekizumab, secukinumab, ustekinumab, and guselkumab, as well as, interestingly, MTX as a csDMARD, were less likely to be switched. These monotherapies should be particularly considered in patients with PsA because these patients tend to have high rates of therapeutic switching [88]. To illustrate the clinical relevance of our chosen model, we have shown that a patient receiving adalimumab monotherapy may be more likely to remain on therapy after 24 weeks than the same patient receiving combination therapy with adalimumab and systemic steroids. This is supported, for example, by a study of a clinical registry of patients with PsA starting biologic and nonbiologic therapy, which compared TNF-α inhibitor monotherapy with MTX monotherapy and their combination and showed that TNF-α inhibitor monotherapy was less likely to be discontinued [89]. Given that a multitude of systemic treatment options with different mechanisms of action are available for PsV and PsA, each with a unique set of benefits, safety risks, dosing schedules, and monitoring requirements [90], ML modeling could provide new insights into the therapeutic options in each clinic and hypothetically also be used to ultimately make decisions about the most optimal therapeutic agent or combination thereof, thus facilitating the personalization of therapy.

While therapeutic modification is of great clinical importance to both clinician and patient, there are also scores used to measure therapeutic effectiveness in patients with PsA and PsV. Therefore, we have sought to identify further features in our secondary clinical data sets that can be modeled around the target scores PASI and BASDAI. Out of 328 potential models, we selected another average blender model with 3 different classifiers for *PASI change after 24 weeks* for detailed evaluation. This aggregate model included the previously presented *XGBoost trees classifier* and the *Eureqa classifier* as well as the *random forest classifier* (using the Gini impurity), an ensemble learning method that works by constructing a large number of decision trees during training and outputs the class of each tree [91]. The Gini impurity measures how often a randomly chosen element would be misclassified and is used as a criterion for splitting decision trees [92]. Further analysis revealed that the primary predictor of PASI score progression was the initial PASI score, followed by changes in pruritus observed at follow-up and therapeutic alterations. Several other factors, ranging from initial symptoms such as pain to characteristics such as body weight, height, baseline pruritus, baseline HADS depression score, and BMI, were found to have minimal impact on the primary target. The most influential feature of this selected model, the PASI score at baseline, has considerable importance in placebo-controlled clinical trials measuring the efficacy of various antipsoriatic treatments [93,94], but the explicit influence of the baseline PASI score on the change in PASI score over time has not been extensively discussed in the current literature. Looking more closely at the impact of individual score values on PASI score improvement, we observed that a PASI score of <2.12 was generally associated with a positive outcome, whereas an initial PASI score of ≥2.12 was associated with a lower likelihood of improvement. This may be interpreted as meaning that patients with psoriasis with minimal skin lesions are more likely to maintain this reduced affected body surface area over the following 6 months if they are under regular medical observation. A key role in determining PASI score progression was also played by variations in pruritus levels throughout the observation period. In particular, a consistent absence of pruritus was found to correlate with a favorable PASI outcome. This observation is important because it is known that the prevalence of pruritus in patients with psoriasis is high (approximately 80%) [95], and there are in fact studies that have shown a significant correlation between the PASI score and the intensity of itching [96]. Finally, we observed that changes in therapy at both 12 and 24 weeks had a slightly negative effect on a positive PASI outcome. This is of particular importance because a frequent change of therapy means that, on the one hand, not enough time has been allowed to determine whether a systemic agent is effective or, on the other hand, that the patient is generally resistant to therapy and may need a more intensive approach to improve their skin lesions. Patient compliance should also be considered because inappropriately frequent medication and dose changes may lead to complications rather than improvement [97].

In the final approach, we investigated whether an initial BASDAI score provides further information about other features of the data set that should be considered when assessing joint involvement in PsA. The BASDAI classification was developed to assess the severity and impact of symptoms such as back pain, joint swelling, areas of local tenderness, and morning stiffness in patients with ankylosing spondylitis [98], but it has since become a commonly used measure of PsA activity independent of axial involvement [99]. Again, we selected an average blender model consisting of a Eureqa generalized additive model classifier, an XGBoost trees classifier with early stopping, and a dropout additive regression trees classifier, which is an extension of the conventional multiple additive regression trees created by incorporating dropout techniques to address the overspecialization problem inherent in the latter [100]. In the context of ML studies on PsA, a study by Lee et al [101] applied CNNs to PsA risk prediction using a large national data set. The prediction model selected in their study had an AUC of 0.7000, with CNNs known for their superior performance on spatial data. By contrast, after training a variety of models on a smaller data set and a different PsA-related target, we were able to select an even more accurate average blender model that identified pain, disease activity, and HADS depression and anxiety scores at baseline as relevant features influencing the target. Indeed, BASDAI score has been shown to correlate with disease activity in ankylosing spondylitis [102], which was also supported by magnetic resonance imaging findings [103]. While there is no literature-supported correlation between the BASDAI score and individual joint pain assessment, a high degree of association with overall pain has been reported [104], which is similar to the pain status recorded in our clinical trials. Finally, we observed that both HADS-A and HADS-D scores of >7 increased the likelihood of a higher BASDAI score. This is important in view of the fact that a score of 8 on both scales is considered to be borderline. In this respect, there is indeed evidence in the literature showing a correlation between both depression and anxiety and the BASDAI score [104,105]. Considering that the HADS score is a widely used self-reporting tool known for its reliability and practicality, clinicians could use it in combination with the pain status of patients with PsA to better assess their joint disease activity. This, in turn, would facilitate more effective and timely adjustments to their treatment.

### Conclusions

Our analytical models have revealed patterns and correlations that might otherwise go unnoticed using traditional analytical methods. This approach brings us closer to personalized medicine, where treatments are optimized based on data-driven insights. While AutoML analysis of medical data sets can play a critical role in advancing health care, it is also vital to recognize and understand its limitations. First, it is important to emphasize that we intended to use AutoML analysis primarily to select highly accurate models that reveal relationships between data set features. With the intent to make predictions, we attempted such an analysis on a reduced data set that contained only the feature indicating therapy change at a future time and excluded all other follow-up features to avoid data leakage. Therefore, predictive modeling of PASI score change and BASDAI score ought to be performed as well with reduced data sets. In addition to reassessing their predictive capacity, the implementation of our models in prospective studies is nonetheless mandatory before their use in daily clinical practice because real-world data may reveal barriers that were not previously considered [106]. Furthermore, such an implementation of an ML model will undoubtedly lead to a so-called data set shift [107], which is a modification of the data that occurs when an implemented model causes changes in practice over time. This requires ongoing remodeling with the shifted data and possibly the replacement of the ML model used with a different one. Nevertheless, the application of AutoML to medical data sets has the potential to revolutionize care, accelerate research, and streamline tasks. This will ensure that health care providers have more time to spend on direct patient care.

## Appendix

Multimedia Appendix 1 Comprehensive data integration flowchart for automated machine learning analysis of psoriasis vulgaris and arthritis primary data sets.

Multimedia Appendix 2 Demographic and clinical profile of the cohort of patients with psoriasis vulgaris and psoriatic arthritis.

Multimedia Appendix 3 Classification approaches for data features of psoriasis vulgaris and psoriatic arthritis clinical data to facilitate automated machine learning analysis.

Multimedia Appendix 4 Missing data imputation techniques for psoriasis vulgaris and psoriatic arthritis data analysis with automated machine learning.

Multimedia Appendix 5 Reduced feature list used for Target 1.1: "Therapy change at 24 weeks follow-up.".

Multimedia Appendix 6 Reduced feature list used for Target 1.2: “Therapy change prediction (only onset features).”.

Multimedia Appendix 7 Reduced feature list used for Target 2: “PASI score change after 24 weeks.”.

Multimedia Appendix 8 Reduced feature list used for Target 3: “BASDAI classification at onset.”.

Multimedia Appendix 9 Hyperparameter optimization strategies for selected machine learning models.

Multimedia Appendix 10 Blueprints of the selected models used during automated machine learning analysis of targets 1 to 3.

Multimedia Appendix 11 Assessment of model performance and learning dynamics for each selected model.

Multimedia Appendix 12 Refined analysis of the “eXtreme Gradient Boosted Trees Classifier” selected for Target 1.1 through the lens of robust uncertainty quantification.

Multimedia Appendix 13 Refined analysis of the “Gradient Boosted Trees Classifier,” “ExtraTrees Classifier (Gini),” and “Eureqa Generalized Additive Model Classifier (1000 Generations)” models selected for the blender model for Target 1.2 through the lens of robust uncertainty quantification.

Multimedia Appendix 14 Refined analysis of the “Random Forest Classifier (Gini),” “eXtreme Gradient Boosted Trees Classifier (learning rate=0.01),” and “Eureqa Classifier (Default Search 3000 Generations)” models selected for the blender model for Target 2 through the lens of robust uncertainty quantification.

Multimedia Appendix 15 Refined analysis of the “Eureqa Generalized Additive Model Classifier (40 Generations),” “eXtreme Gradient Boosted Trees Classifier with Early Stopping,” and “Dropout Additive Regression Trees Classifier (15 leaves)” models selected for the blender model for Target 3 through the lens of robust uncertainty quantification.

Multimedia Appendix 16 This figure summarizes the compliance of this study with the guidelines of the Transparent Reporting of a Multivariable Prediction Model for Individual Prognosis or Diagnosis (TRIPOD) Initiative. Each section of the TRIPOD checklist is addressed, detailing aspects of prediction model development such as study objectives, data sources, eligibility criteria, outcomes, predictors, statistical analysis methods, and model performance metrics.

Multimedia Appendix 17 Holdout partition receiver operating characteristic (ROC) curves. (S1) ROC curve during holdout for Target 1.1: “Therapy change at 24 weeks follow-up” with a threshold of 0.7091. (S2) ROC curve during holdout for Target 2: “PASI score change after 24 weeks” with a threshold value of 0.549. (S3) ROC curve during holdout for Target 3: “BASDAI classification at onset” with a threshold set at 0.453.

Multimedia Appendix 18 Therapy change prediction scenario for 2 hypothetical patients with psoriasis.

Multimedia Appendix 19 List of feature effects of the selected average blender model for Target 2 “PASI score change after 24 weeks.” All features are listed from most to least influential based on normalized impact, providing insight into the factors that contribute most to the prediction of changes in the Psoriasis Area and Severity Index score.

## Abbreviations

AUC: area under the receiver operating characteristic curve
AutoML: automated machine learning
BASDAI: Bath Ankylosing Spondylitis Disease Activity Index
CASPAR: Classification Criteria for Psoriatic Arthritis
CNN: convolutional neural network
csDMARD: conventional synthetic disease-modifying antirheumatic drug
CV: cross-validation
DLQI: Dermatology Life Quality Index
EDA: exploratory data analysis
HADS: Hospital Anxiety and Depression Scale
IL: interleukin
LogLoss: logarithmic loss
MCC: maximum Matthews correlation coefficients
ML: machine learning
MTX: methotrexate
NRS: numeric rating scale
PASI: Psoriasis Area and Severity Index
PD: partial dependence
PsA: psoriatic arthritis
PsV: psoriasis vulgaris
QoL: quality of life
RQ: research question
TNF-α: tumor necrosis factor alpha
TRIPOD: Transparent Reporting of a Multivariable Prediction Model for Individual Prognosis or Diagnosis
XGBoost: extreme gradient boosted

## Data Availability

The pseudonymized secondary data set generated during and analyzed during this study is deposited on the web [47].
