# Supplementary material for: Using Automated Machine Learning to Predict Necessary Upcoming Therapy Changes in Patients With Psoriasis Vulgaris and Psoriatic Arthritis and Uncover New Influences on Disease Progression: Retrospective Study
Source: JMIR Form Res. 2024 Jun 27;8:e55855. doi: 10.2196/55855 (PMC11240079; doi:10.2196/55855)
Supplement: Multimedia Appendix 1 [file formative_v8i1e55855_app1.pdf]

Multimedia Appendix 1

Comprehensive data integration flowchart for AutoML analysis of psoriasis vulgaris and arthritis primary datasets

Primary datasets: initial enrollment and follow-up features

Patient data

Age, weight, gender, height, skin type (Fitzpatrick), age of onset, time since diagnosis, duration of illness, previous illnesses, allergies, hip/abdominal circumference, heart rate, systolic and diastolic blood pressure

Study app data

Message texts, images of skin lesions, details on the use of questions and their answers, DLQI, several NRS answers on pain, itching, affected mood, morning stiffness duration and intensity, joint swelling

Imaging

Photo documentation, pseudonymised images of skin and joints, chest X-rays, joint sonograms, MRI, CT, scintigraphy

Blood examination

Liver/kidney values, CRP, BSG, rheumatoid factor, anti-CCP-AK, ANA, ENA-AK, ds-DNA-AK, ANCA, HLA-B27, QuantiFERON test, hepatitis B/-C, HIV

Medication

Substance classes, dosage and intake intervals, therapy changes, dose changes, side effects, previous therapies

Social anamnesis

Family status, nationality, employment status, highest school-leaving qualification, type of occupation, social interactions, social contacts, smartphone ownership and use, hobbies, pets, holidays

Lifestyle

Nicotine, drug and alcohol consumption, sport, nutrition

Scores

Arthritis 66/68 (swollen, tender joint count)
ASDAS (Ankylosing Spondylitis Disease Activity Score)
BASDAI (Bath Ankylosing Spondylitis Disease Activity Index)
BASFI (Bath Ankylosing Spondylitis Functional Index)
BASMI (Bath Ankylosing Spondylitis Metrology Index)
CASPAR (Classification Criteria for Psoriatic Arthritis)
CDAI (Clinical Disease activity Score)
DAPSA (Disease Activity in Psoriatic Arthritis)
DAS28 (Disease Activity Score 28)
DLQI (Dermatology Life Quality Index)
HADS-A/-D (Hospital Anxiety and Depression Scale)
LDI (Leeds Dactylitis Index)
MARS (Medication Adherence Report Scale)
NRS (Numerical Rating Scale)
NRS Compliance, Pain, Itch, Adherence
PASI (Psoriasis Area Severity Index)
PGA (Physician Global Assessment)
SDAI (Simplified Disease activity Score)
SPARCC (Spondylarthritis Research Con. of Canada)
VAS (Visual Analogue Scale)

Data preparation

- *Grouping of patients with common features at enrollment and follow ups*
- *feature exclusion if:*
  - *not common in all datasets*
  - *>50% missing values*
- *creation of new features:*
  - *BMI, physical activity level, app usage*
- *reclassification of numeric features to binary or multiclass*
- *calculation of feature changes over time*

Secondary dataset (82 features)

Patient data

Age, weight, height, gender, BMI

Study app data

usage, questions, median values (pruritus, pain, DLQI, affected mood, morning stiffness)

Medication

Substance classes and targets, therapy changes, topical therapy

Lifestyle

Nicotine, alcohol consumption, sport

Scores (numeric values, classification, changes over time)

BASDAI (Bath Ankylosing Spondylitis Disease Activity Index)
CASPAR (Classification Criteria for Psoriatic Arthritis)
DLQI (Dermatology Life Quality Index)
HADS-A/-D (Hospital Anxiety and Depression Scale)
NRS (Numerical Rating Scale)
NRS Compliance, Pain, Itch, Adherence
PASI (Psoriasis Area Severity Index)

AutoML

Data preparation and integration process from 2 primary datasets of psoriasis vulgaris and arthritis patients. This flowchart illustrates the data consolidation steps from 2 clinical trials conducted in our Department of Dermatology between 2018 and 2021. The initial datasets included a comprehensive range of features, including patient demographics, app-derived symptom documentation, medical imaging and blood tests. Preparation involved excluding features that were not common to both datasets, developing new features, and reclassifying existing features. The resulting secondary dataset contained 82 refined features that were aligned to facilitate AutoML analysis.
