# Supplementary material for: Using Automated Machine Learning to Predict Necessary Upcoming Therapy Changes in Patients With Psoriasis Vulgaris and Psoriatic Arthritis and Uncover New Influences on Disease Progression: Retrospective Study
Source: JMIR Form Res. 2024 Jun 27;8:e55855. doi: 10.2196/55855 (PMC11240079; doi:10.2196/55855)
Supplement: Multimedia Appendix 2 [file formative_v8i1e55855_app2.pdf]

Multimedia Appendix 2

Demographic and clinical profile of the psoriasis vulgaris and psoriatic arthritis cohort

Study cohort characteristics – secondary dataset	
**Sex**	**n (%)**
Female	122 (39.48)
Male	187 (60.52)
Missing values	0 (0)
**Age** (years)	
Mean (SD)	49.69 (13.47)
Median (IQR)	52 (20)
Min; Max	21; 84
Missing values	0 (0)
**Body weight** (kg)	
Mean (SD)	88.85 (20.85)
Median (IQR)	86 (25)
Min; Max	48; 180
Missing values	2 (0.65)
**Body height** (cm)	
Mean (SD)	173.77 (9.34)
Median (IQR)	174 (15)
Min; Max	152; 194
Missing values	2 (0.65)
**Smoking**	**n (%)**
Yes	114 (37.62%)
Missing values	6 (1.94)

**Alcohol** (consumption count in the last 7 days)	
Mean (SD)	1.28 (1.81)
Median (IQR)	1 (2)
Min; Max	0; 8
Missing values	7 (2.27%)
**Sports** (> 2h/week)	**n (%)**
Yes	134 (43.37%)
Missing values	7 (2.27%)
**DLQI** (0 – 30 pts.)	
Mean (SD)	7.25 (7.79)
Median (IQR)	4 (1)
Min; Max	0; 30
Missing values	2 (0.65)
**HADS-A** (0 – 21 pts.)	
Mean (SD)	6.56 (4.40)
Median (IQR)	6 (3)
Min; Max	0; 20
Missing values	2 (0.65)
**HADS-D** (0 – 21 pts.)	
Mean (SD)	4.94 (4.31)
Median (IQR)	4 (2)
Min; Max	0; 19
Missing values	2 (0.65)

**PASI** (0 – 72 pts.)	
Mean (SD)	5.20 (6.93)
Median (IQR)	2.7 (0.6)
Min; Max	0; 53.2
Missing values	0 (0)
**BASDAI** (0 – 10 pts.)	
Mean (SD)	3.48 (2.21)
Median (IQR)	3.1 (1.7)
Min; Max	0; 9.1
Missing values	180 (58.25%)

Demographic and clinical profile of the psoriasis vulgaris and psoriatic arthritis cohort. This figure presents a detailed statistical summary of the demographic and clinical characteristics of patients with PsV and PsA from the secondary dataset. It includes a comprehensive breakdown of gender distribution, detailed age metrics, body measurements, lifestyle habits such as smoking and alcohol consumption, and critical clinical scores including the Dermatology Life Quality Index (DLQI), Hospital Anxiety and Depression Scale-Anxiety (HADS-A), Hospital Anxiety and Depression Scale-Depression (HADS-D), Psoriasis Area Severity Index (PASI), and Bath Ankylosing Spondylitis Disease Activity Index (BASDAI). Data points include mean, standard deviation (SD), median with interquartile range (IQR), and minimum and maximum values. It also indicates the proportion of missing data where relevant, providing a quantitative snapshot of the population characteristics of the study.
