# Supplementary material for: Using Automated Machine Learning to Predict Necessary Upcoming Therapy Changes in Patients With Psoriasis Vulgaris and Psoriatic Arthritis and Uncover New Influences on Disease Progression: Retrospective Study
Source: JMIR Form Res. 2024 Jun 27;8:e55855. doi: 10.2196/55855 (PMC11240079; doi:10.2196/55855)
Supplement: Multimedia Appendix 3 [file formative_v8i1e55855_app3.pdf]

Multimedia Appendix 3

Classification approaches for data features of psoriasis vulgaris and psoriatic arthritis clinical data to facilitate AutoML analysis

Feature name	Feature type	New classification
New DLQI classification	Multiclass	no influence, if DLQI score 0-1<br>little influence, if DLQI score 2-5<br>medium influence, if DLQI score 6-10<br>strong influence, if DLQI score >11
New HADS anxiety classification	Multiclass	inconspicuous, of HADS-A score 0-7<br>borderline, if HADS-A score 8-10<br>conspicuous, if HADS-A score >10
New HADS depression classification	Multiclass	inconspicuous, of HADS-D score 0-7<br>borderline, if HADS-D score 8-10<br>conspicuous, if HADS-D score >10
New BASDAI classification	Binary	1, if BASDAI score ≥4<br>0, if BASDAI score ≤3
New CASPAR classification	Binary	1, if CASPAR score ≥3<br>0, if CASPAR score ≤2
Job type assessment	Multiclass	physical work<br>mostly sedentary work<br>unemployed<br>student<br>retired
Physical activity at onset	Multiclass	S= sports, P= physical activity<br>nSnP; ySnP; nSyP; ySyP
Systemic target	Multiclass	IL-17<br>IL-23<br>IL-12+IL-23<br>TNF-α<br>csDMARDs<br>fumaric acid ester<br>retinoid<br>dimethyl fumarate<br>PDE 4<br>systemic steroids

Therapy with TNF-α inhibitors at onset	Binary	1, if treated with a TNF-α inhibitor<br>0, if not treated with TNF-αinhibitor
Therapy with IL-17 Inhibitors at onset	Binary	1, if treated with an IL-17 inhibitor<br>0, if not treated with an IL-17 inhibitor
Therapy with IL-23 inhibitors at onset	Binary	1, if treated with an IL-23 inhibitor<br>0, if not treated with an IL-23 inhibitor
Therapy with IL-12/23 inhibitor at onset	Binary	1, if treated with Ustekinumab<br>0, if not treated with Ustekinumab
Therapy with csDMARDs at onset	Binary	1, if treated with csDMARDs<br>0, if not treated with csDMARDs
Therapy with others then b-/csDMARDs	Binary	1, if treated with others<br>0, if not treated with others
Diagnosed depression at onset	Binary	1, if the patient had a history of depression at study entry<br>0, if depression was not in the patient's history at study entry
Diagnosed metabolic syndrome at onset	Binary	1, if the patient had a history of metabolic syndrome at study entry<br>0, if the patient did not have metabolic syndrome at baseline
Diagnosed coronary heart disease at onset	Binary	1, if the patient had a history of coronary artery disease at study entry<br>0, if coronary artery disease was not in the patient's history at study entry
Diagnosed arterial hypertension at onset	Binary	1, if the patient had a history of arterial hypertension at study entry<br>0, if arterial hypertension was not in the patient's history at study entry
No diagnosed pre-existing illness at onset	Binary	1, if the patient had other pre-existing illnesses at study entry<br>0, if other pre-existing illnesses were not in the patient's history at study entry

Obesity at onset	Binary	1, if BMI was ≥ $30 kg/m^2$<br>0, if BMI was < $30 kg/m^2$
Therapy change binary	Binary	1, if there was a change in therapy during the study period<br>0, if there was no change in therapy during the study period
Therapy change differential	Multiclass	at onset<br>after 3 months<br>after 6 months<br>at onset and after 3 months<br>at onset and after 6 months<br>at onset, after 3 and 6 months<br>after 3 and 6 months
Topical therapy duration over 24 weeks	Multiclass	Not used<br>3 months, if used at baseline up to 12 weeks of study or after 12 weeks of study up to 24 weeks of study<br>6 months, if used at baseline to V2

Many of the scores in the primary data sets were only available as numeric values. They were therefore classified. This was done with the values at baseline, after 12 weeks of follow-up and after 24 weeks of follow-up. Patients were also classified according to their daily physical activity at work. In the first primary dataset, the daily physical activity at work was asked. If it was more than 2h/day, it was classified as physical work. If it was less than that, it was described as mostly sedentary work. In the other dataset, only the job title was given. Here the classification was done individually, depending on the type of job. Predominantly sedentary occupations include commercial clerk, engineer, translator, taxi driver, insurance salesman, technical draughtsman, web designer, office clerk, accountant, lorry driver, youth worker, administrator, drug developer, analyst, merchant, software tester, sales manager, retail clerk, medical assistant, computer scientist, musician, electrical engineer, divisional manager, international trade, IT manager, sales representative, data engineer, industrial clerk, administrative assistant, clerical assistant, student assistant, IT coordinator, tax assistant, event organizer, sales manager, business development manager, facilities consultant, MTLA, practice assistant, general manager, branch manager, fleet dispatcher, banker, architect and medical assistant. Manual occupations with an expected physical activity of more than 2h/day include petrol station attendant, mechanical technician, postman, construction worker, CNC lathe operator, service worker, mechanic, social worker, training supervisor, production assistant, two-wheeler mechanic, logistics manager, packer, gas and water fitter, service technician, life support, homemaker, telecommunications installer, showman, geriatric nurse, painter, machinist, florist, chemical worker, winemaker, electrician, carpenter, emergency paramedic, educator, wood cleaner, cook, machine operator, plasterer foreman, chemical foreman, production worker, electrician installer, delivery printer worker, assembler, geriatric nurse, medical assistant, warehouse logistician, handyman, petrol station maintenance, internist, air conditioning technician, locksmith and excavation worker. Other groups included people who were retired people, job seekers, and students. Students included school students and trainees. Sports activity was then compared with physical workload during the working day. Patients were given a yes or no classification for sports and physical work. If the patients did more than 2 hours of sports per week, they were given the label "yS." Patients who did less or no sports were labeled "nS." If the patients were engaged in physical work "yP" and if it was a predominantly sedentary occupation, the patient was a jobseeker, retired or a student "nP." Together these resulted in the classes "ySyP," "ySnP," "nSyP" and "nSnP." Finally, the systemic therapies were grouped according to the drug target.
