# Supplementary material for: Using Automated Machine Learning to Predict Necessary Upcoming Therapy Changes in Patients With Psoriasis Vulgaris and Psoriatic Arthritis and Uncover New Influences on Disease Progression: Retrospective Study
Source: JMIR Form Res. 2024 Jun 27;8:e55855. doi: 10.2196/55855 (PMC11240079; doi:10.2196/55855)
Supplement: Multimedia Appendix 4 [file formative_v8i1e55855_app4.pdf]

Multimedia Appendix 4

Missing data imputation techniques for psoriasis vulgaris and psoriatic arthritis data analysis with AutoML

This table details the imputation strategies used to address missing values in selected machine learning models for each target. The imputation descriptions outline the logic behind each imputed value, providing transparency into the machine learning process and ensuring the integrity of subsequent analyses. These methods facilitated the comprehensive handling of missing data across a range of features collected during the clinical trials, ensuring robust predictive modeling of patient outcomes.

Data Handling Report

Target 1.1: eXtreme Gradient Boosted Trees Classifier with Early Stopping

Feature Name	Var Type	Missing Count	Missing Percentage	Imputation Name	Imputation Description
BASDAI score at onset	Numeric	81	53	Missing Values Imputed	Missing indicator treated as feature, Imputed value: 2.8
Systemic treatment at onset	Categorical	41	27	Ordinal encoding of categorical variables	Imputed value: -2
CASPAR score at onset	Numeric	39	26	Missing Values Imputed	Missing indicator treated as feature, Imputed value: 3
Diagnosed other disease at onset	Categorical	33	22	Ordinal encoding of categorical variables	Imputed value: -2
DLQI classification change over 24 weeks	Categorical	16	11	Ordinal encoding of categorical variables	Imputed value: -2
HADS-A classification change over 24 weeks	Categorical	16	11	Ordinal encoding of categorical variables	Imputed value: -2
HADS-D classification change over 24 weeks	Categorical	16	11	Ordinal encoding of categorical variables	Imputed value: -2
Pruritus change over 24 weeks	Categorical	15	10	Ordinal encoding of categorical variables	Imputed value: -2
PASI change after 24 weeks differential	Categorical	15	10	Ordinal encoding of categorical variables	Imputed value: -2
Alcohol	Numeric	4	3	Missing Values Imputed	Missing indicator treated as feature, Imputed value: 1
Smoking	Numeric	3	2	Missing Values Imputed	Missing indicator treated as feature, Imputed value: 0
Occupation	Categorical	2	1	Ordinal encoding of categorical variables	Imputed value: -2
Sports	Numeric	2	1	Missing Values Imputed	Missing indicator treated as feature, Imputed value: 0

BMI	Numeric	1	1	Missing Values Imputed	Missing indicator treated as feature, Imputed value: 29.0174
Physical activity at onset	Categorical	0	0	Ordinal encoding of categorical variables	Imputed value: -2
DLQI classification at onset	Categorical	0	0	Ordinal encoding of categorical variables	Imputed value: -2
HADS-A classification at onset	Categorical	0	0	Ordinal encoding of categorical variables	Imputed value: -2
HADS-D classification at onset	Categorical	0	0	Ordinal encoding of categorical variables	Imputed value: -2
Topical therapy duration over 24 weeks	Categorical	0	0	Ordinal encoding of categorical variables	Imputed value: -2
Age	Numeric	0	0	Missing Values Imputed	Imputed value: 52
Topical therapy at onset	Numeric	0	0	Missing Values Imputed	Imputed value: 0

Target 1.2: AVG Blender (Gradient Boosted Trees Classifier, ExtraTrees Classifier (Gini), Eureqa Generalized Additive Model Classifier (1000 Generations))

Gradient Boosted Trees Classifier

Feature Name	Var Type	Missing Count	Missing Percentage	Imputation Name	Imputation Description
Diagnosed other disease at onset	Categorical	106	70	Ordinal encoding of categorical variables	Imputed value: -2
BASDAI score at onset	Numeric	81	53	Missing Values Imputed	Missing indicator treated as feature, Imputed value: 3
BASDAI classification at onset	Numeric	81	53	Missing Values Imputed	Imputed value: 0
Disease activity (NRS) at onset	Numeric	50	33	Missing Values Imputed	Missing indicator treated as feature, Imputed value: 2
Pain (NRS) at onset	Numeric	49	32	Missing Values Imputed	Missing indicator treated as feature, Imputed value: 3
CASPAR classification at onset	Numeric	46	30	Missing Values Imputed	Missing indicator treated as feature, Imputed value: 1
CASPAR score at onset	Numeric	46	30	Missing Values Imputed	Imputed value: 3
Systemic target at onset	Categorical	40	26	Ordinal encoding of categorical variables	Imputed value: -2
Systemic treatment at onset	Categorical	39	26	Ordinal encoding of categorical variables	Imputed value: -2
Alcohol	Numeric	3	2	Missing Values Imputed	Missing indicator treated as feature, Imputed value: 1
Pruritus (NRS) at onset	Numeric	3	2	Missing Values Imputed	Missing indicator treated as feature, Imputed value: 2
Occupation	Categorical	2	1	Ordinal encoding of categorical variables	Imputed value: -2

Smoking	Numeric	1	1	Missing Values Imputed	Missing indicator treated as feature, Imputed value: 0
Gender	Categorical	0	0	Ordinal encoding of categorical variables	Imputed value: -2
Physical activity at onset	Categorical	0	0	Ordinal encoding of categorical variables	Imputed value: -2
DLQI classification at onset	Categorical	0	0	Ordinal encoding of categorical variables	Imputed value: -2
HADS-A classification at onset	Categorical	0	0	Ordinal encoding of categorical variables	Imputed value: -2
HADS-D classification at onset	Categorical	0	0	Ordinal encoding of categorical variables	Imputed value: -2
Age	Numeric	0	0	Missing Values Imputed	Imputed value: 52
Body height at onset	Numeric	0	0	Missing Values Imputed	Imputed value: 173
Body weight at onset	Numeric	0	0	Missing Values Imputed	Imputed value: 89
BMI	Numeric	0	0	Missing Values Imputed	Imputed value: 28.7037
Obesity at onset	Numeric	0	0	Missing Values Imputed	Imputed value: 0
Diagnosed depression at onset	Numeric	0	0	Missing Values Imputed	Imputed value: 0
Diagnosed arterial hypertension at onset	Numeric	0	0	Missing Values Imputed	Imputed value: 0
No diagnosed pre-existing illness at onset	Numeric	0	0	Missing Values Imputed	Imputed value: 0
Diagnosed coronary heart disease at onset	Numeric	0	0	Missing Values Imputed	Imputed value: 0
Diagnosed metabolic disease at onset	Numeric	0	0	Missing Values Imputed	Imputed value: 0
Sports	Numeric	0	0	Missing Values Imputed	Imputed value: 0
DLQI classification at onset	Numeric	0	0	Missing Values Imputed	Imputed value: 4
HADS-A score at onset	Numeric	0	0	Missing Values Imputed	Imputed value: 6
HADS-D score at onset	Numeric	0	0	Missing Values Imputed	Imputed value: 4
Therapy with TNF-α inhibitors at onset	Numeric	0	0	Missing Values Imputed	Imputed value: 0
Therapy with IL-17 Inhibitors at onset	Numeric	0	0	Missing Values Imputed	Imputed value: 0
Therapy with IL-12/23 Inhibitors at onset	Numeric	0	0	Missing Values Imputed	Imputed value: 0
Therapy with IL-23 Inhibitors at onset	Numeric	0	0	Missing Values Imputed	Imputed value: 0
Therapy with csDMARDs at onset	Numeric	0	0	Missing Values Imputed	Imputed value: 0
Therapy with others then b-/csDMARDs	Numeric	0	0	Missing Values Imputed	Imputed value: 0
Topical therapy at onset	Numeric	0	0	Missing Values Imputed	Imputed value: 0
PASI score at onset	Numeric	0	0	Missing Values Imputed	Imputed value: 2.4

ExtraTrees Classifier (Gini)

Feature Name	Var Type	Missing Count	Missing Percentage	Imputation Name	Imputation Description
BASDAI score at onset	Numeric	81	53	Missing Values Imputed	Missing indicator treated as feature, Imputed value: 3
BASDAI classification at onset	Numeric	81	53	Missing Values Imputed	Imputed value: 0
Disease activity (NRS) at onset	Numeric	50	33	Missing Values Imputed	Missing indicator treated as feature, Imputed value: 2
Pain (NRS) at onset	Numeric	49	32	Missing Values Imputed	Missing indicator treated as feature, Imputed value: 3
CASPAR classification at onset	Numeric	46	30	Missing Values Imputed	Missing indicator treated as feature, Imputed value: 1
CASPAR score at onset	Numeric	46	30	Missing Values Imputed	Imputed value: 3
Alcohol	Numeric	3	2	Missing Values Imputed	Missing indicator treated as feature, Imputed value: 1
Pruritus (NRS) at onset	Numeric	3	2	Missing Values Imputed	Missing indicator treated as feature, Imputed value: 2
Smoking	Numeric	1	1	Missing Values Imputed	Missing indicator treated as feature, Imputed value: 0
Gender	Categorical	0	0	One-Hot Encoding	Missing values ignored
Age	Numeric	0	0	Missing Values Imputed	Imputed value: 52
Body height at onset	Numeric	0	0	Missing Values Imputed	Imputed value: 173
Body weight at onset	Numeric	0	0	Missing Values Imputed	Imputed value: 89
BMI	Numeric	0	0	Missing Values Imputed	Imputed value: 28.7037
Obesity at onset	Numeric	0	0	Missing Values Imputed	Imputed value: 0
Diagnosed depression at onset	Numeric	0	0	Missing Values Imputed	Imputed value: 0
Diagnosed arterial hypertension at onset	Numeric	0	0	Missing Values Imputed	Imputed value: 0
No diagnosed pre-existing illness at onset	Numeric	0	0	Missing Values Imputed	Imputed value: 0
Diagnosed coronary heart disease at onset	Numeric	0	0	Missing Values Imputed	Imputed value: 0
Diagnosed metabolic disease at onset	Numeric	0	0	Missing Values Imputed	Imputed value: 0
Sports	Numeric	0	0	Missing Values Imputed	Imputed value: 0
DLQI classification at onset	Numeric	0	0	Missing Values Imputed	Imputed value: 4
HADS-A score at onset	Numeric	0	0	Missing Values Imputed	Imputed value: 6
HADS-D score at onset	Numeric	0	0	Missing Values Imputed	Imputed value: 4
Therapy with TNF-α inhibitors at onset	Numeric	0	0	Missing Values Imputed	Imputed value: 0
Therapy with IL-17 Inhibitors at onset	Numeric	0	0	Missing Values Imputed	Imputed value: 0

Therapy with IL-12/23 Inhibitors at onset	Numeric	0	0	Missing Values Imputed	Imputed value: 0
Therapy with IL-23 Inhibitors at onset	Numeric	0	0	Missing Values Imputed	Imputed value: 0
Therapy with csDMARDs at onset	Numeric	0	0	Missing Values Imputed	Imputed value: 0
Therapy with others then b-/csDMARDs	Numeric	0	0	Missing Values Imputed	Imputed value: 0
Topical therapy at onset	Numeric	0	0	Missing Values Imputed	Imputed value: 0
PASI score at onset	Numeric	0	0	Missing Values Imputed	Imputed value: 2.4

Eureqa Generalized Additive Model Classifier (1000 Generations)

Feature Name	Var Type	Missing Count	Missing Percentage	Imputation Name	Imputation Description
Diagnosed other disease at onset	Categorical	106	70	One-Hot Encoding	Missing indicator treated as feature
BASDAI score at onset	Numeric	81	53	Missing Values Imputed	Missing indicator treated as feature, Imputed value: 3
BASDAI classification at onset	Numeric	81	53	Missing Values Imputed	Imputed value: 0
Disease activity (NRS) at onset	Numeric	50	33	Missing Values Imputed	Missing indicator treated as feature, Imputed value: 2
Pain (NRS) at onset	Numeric	49	32	Missing Values Imputed	Missing indicator treated as feature, Imputed value: 3
CASPAR classification at onset	Numeric	46	30	Missing Values Imputed	Missing indicator treated as feature, Imputed value: 1
CASPAR score at onset	Numeric	46	30	Missing Values Imputed	Imputed value: 3
Systemic target at onset	Categorical	40	26	One-Hot Encoding	Missing indicator treated as feature
Systemic treatment at onset	Categorical	39	26	One-Hot Encoding	Missing indicator treated as feature
Alcohol	Numeric	3	2	Missing Values Imputed	Missing indicator treated as feature, Imputed value: 1
Pruritus (NRS) at onset	Numeric	3	2	Missing Values Imputed	Missing indicator treated as feature, Imputed value: 2
Occupation	Categorical	2	1	One-Hot Encoding	Missing values treated as infrequent
Smoking	Numeric	1	1	Missing Values Imputed	Missing indicator treated as feature, Imputed value: 0
Gender	Categorical	0	0	One-Hot Encoding	Missing values ignored
Physical activity at onset	Categorical	0	0	One-Hot Encoding	Missing values ignored
DLQI classification at onset	Categorical	0	0	One-Hot Encoding	Missing values ignored

HADS-A classification at onset	Categorical	0	0	One-Hot Encoding	Missing values ignored
HADS-D classification at onset	Categorical	0	0	One-Hot Encoding	Missing values ignored
Age	Numeric	0	0	Missing Values Imputed	Imputed value: 52
Body height at onset	Numeric	0	0	Missing Values Imputed	Imputed value: 173
Body weight at onset	Numeric	0	0	Missing Values Imputed	Imputed value: 89
BMI	Numeric	0	0	Missing Values Imputed	Imputed value: 28.7037
Obesity at onset	Numeric	0	0	Missing Values Imputed	Imputed value: 0
Diagnosed depression at onset	Numeric	0	0	Missing Values Imputed	Imputed value: 0
Diagnosed arterial hypertension at onset	Numeric	0	0	Missing Values Imputed	Imputed value: 0
No diagnosed pre-existing illness at onset	Numeric	0	0	Missing Values Imputed	Imputed value: 0
Diagnosed coronary heart disease at onset	Numeric	0	0	Missing Values Imputed	Imputed value: 0
Diagnosed metabolic disease at onset	Numeric	0	0	Missing Values Imputed	Imputed value: 0
Sports	Numeric	0	0	Missing Values Imputed	Imputed value: 0
DLQI classification at onset	Numeric	0	0	Missing Values Imputed	Imputed value: 4
HADS-A score at onset	Numeric	0	0	Missing Values Imputed	Imputed value: 6
HADS-D score at onset	Numeric	0	0	Missing Values Imputed	Imputed value: 4
Therapy with TNF-α inhibitors at onset	Numeric	0	0	Missing Values Imputed	Imputed value: 0
Therapy with IL-17 Inhibitors at onset	Numeric	0	0	Missing Values Imputed	Imputed value: 0
Therapy with IL-12/23 Inhibitors at onset	Numeric	0	0	Missing Values Imputed	Imputed value: 0
Therapy with IL-23 Inhibitors at onset	Numeric	0	0	Missing Values Imputed	Imputed value: 0
Therapy with csDMARDs at onset	Numeric	0	0	Missing Values Imputed	Imputed value: 0
Therapy with others then b-/csDMARDs	Numeric	0	0	Missing Values Imputed	Imputed value: 0
Topical therapy at onset	Numeric	0	0	Missing Values Imputed	Imputed value: 0
PASI score at onset	Numeric	0	0	Missing Values Imputed	Imputed value: 2.4

Target 2: AVG Blender (RandomForest Classifier (Gini), eXtreme Gradient Boosted Trees Classifier (learning rate=0.01), Eureqa Classifier (Default Search 3000 Generations))

RandomForest Classifier (Gini)

Feature Name	Var Type	Missing Count	Missing Percentage	Imputation Name	Imputation Description
Diagnosed other disease at onset	Categorical	93	62	Ordinal encoding of categorical variables	Imputed value: -2
BASDAI score at onset	Numeric	77	51	Missing Values Imputed	Missing indicator treated as feature, Imputed value: 2.8
BASDAI classification at onset	Numeric	77	51	Missing Values Imputed	Imputed value: 0
Systemic treatment at onset	Categorical	50	33	Ordinal encoding of categorical variables	Imputed value: -2
Systemic target at onset	Categorical	50	33	Ordinal encoding of categorical variables	Imputed value: -2
Pain (NRS) at onset	Numeric	45	30	Missing Values Imputed	Missing indicator treated as feature, Imputed value: 3
Disease activity (NRS) at onset	Numeric	45	30	Missing Values Imputed	Imputed value: 2
CASPAR classification at onset	Numeric	40	26	Missing Values Imputed	Missing indicator treated as feature, Imputed value: 1
CASPAR score at onset	Numeric	40	26	Missing Values Imputed	Imputed value: 3
Pain change over 24 weeks	Categorical	39	26	Ordinal encoding of categorical variables	Imputed value: -2
Therapy change differential	Categorical	18	12	Ordinal encoding of categorical variables	Imputed value: -2
Therapy change binary	Numeric	18	12	Missing Values Imputed	Missing indicator treated as feature, Imputed value: 1
Occupation	Categorical	2	1	Ordinal encoding of categorical variables	Imputed value: -2
Pruritus change over 24 weeks	Categorical	2	1	Ordinal encoding of categorical variables	Imputed value: -2
Obesity at onset	Numeric	2	1	Missing Values Imputed	Missing indicator treated as feature, Imputed value: 0
DLQI classification change over 24 weeks	Categorical	1	1	Ordinal encoding of categorical variables	Imputed value: -2
HADS-A classification change over 24 weeks	Categorical	1	1	Ordinal encoding of categorical variables	Imputed value: -2
HADS-D classification change over 24 weeks	Categorical	1	1	Ordinal encoding of categorical variables	Imputed value: -2
Body height at onset	Numeric	1	1	Missing Values Imputed	Missing indicator treated as feature, Imputed value: 173
Body weight at onset	Numeric	1	1	Missing Values Imputed	Imputed value: 86
BMI	Numeric	1	1	Missing Values Imputed	Imputed value: 27.7

Alcohol	Numeric	1	1	Missing Values Imputed	Missing indicator treated as feature, Imputed value: 1
Sports	Numeric	1	1	Missing Values Imputed	Missing indicator treated as feature, Imputed value: 0
Patient_ID	Categorical	0	0	Ordinal encoding of categorical variables	Imputed value: -2
Gender	Categorical	0	0	Ordinal encoding of categorical variables	Imputed value: -2
Physical activity at onset	Categorical	0	0	Ordinal encoding of categorical variables	Imputed value: -2
DLQI classification at onset	Categorical	0	0	Ordinal encoding of categorical variables	Imputed value: -2
HADS-A classification at onset	Categorical	0	0	Ordinal encoding of categorical variables	Imputed value: -2
HADS-D classification at onset	Categorical	0	0	Ordinal encoding of categorical variables	Imputed value: -2
Topical therapy duration over 24 weeks	Categorical	0	0	Ordinal encoding of categorical variables	Imputed value: -2
app used	Categorical	0	0	Ordinal encoding of categorical variables	Imputed value: -2
Age	Numeric	0	0	Missing Values Imputed	Imputed value: 52
Smoking	Numeric	0	0	Missing Values Imputed	Imputed value: 0
Diagnosed depression at onset	Numeric	0	0	Missing Values Imputed	Imputed value: 0
Diagnosed arterial hypertension at onset	Numeric	0	0	Missing Values Imputed	Imputed value: 0
No diagnosed pre-existing illness at onset	Numeric	0	0	Missing Values Imputed	Imputed value: 0
Diagnosed coronary heart disease at onset	Numeric	0	0	Missing Values Imputed	Imputed value: 0
Diagnosed metabolic disease at onset	Numeric	0	0	Missing Values Imputed	Imputed value: 0
DLQI classification at onset	Numeric	0	0	Missing Values Imputed	Imputed value: 4
HADS-A score at onset	Numeric	0	0	Missing Values Imputed	Imputed value: 6
HADS-D score at onset	Numeric	0	0	Missing Values Imputed	Imputed value: 4
Therapy with TNF-α inhibitors at onset	Numeric	0	0	Missing Values Imputed	Imputed value: 0
Therapy with IL-17 Inhibitors at onset	Numeric	0	0	Missing Values Imputed	Imputed value: 0
Therapy with IL-12/23 Inhibitors at onset	Numeric	0	0	Missing Values Imputed	Imputed value: 0
Therapy with IL-23 Inhibitors at onset	Numeric	0	0	Missing Values Imputed	Imputed value: 0
Therapy with csDMARDs at onset	Numeric	0	0	Missing Values Imputed	Imputed value: 0
Therapy with others then b-/csDMARDs	Numeric	0	0	Missing Values Imputed	Imputed value: 0
Topical therapy at onset	Numeric	0	0	Missing Values Imputed	Imputed value: 0

Pruritus (NRS) at onset	Numeric	0	0	Missing Values Imputed	Imputed value: 1
PASI score at onset	Numeric	0	0	Missing Values Imputed	Imputed value: 2.4

eXtreme Gradient Boosted Trees Classifier (learning rate=0.01)

Feature Name	Var Type	Missing Count	Missing Percentage	Imputation Name	Imputation Description
Diagnosed other disease at onset	Categorical	93	62	One-Hot Encoding	Missing indicator treated as feature
BASDAI score at onset	Numeric	77	51	Missing Values Imputed	Missing indicator treated as feature, Imputed value: 2.8
BASDAI classification at onset	Numeric	77	51	Missing Values Imputed	Imputed value: 0
Systemic treatment at onset	Categorical	50	33	One-Hot Encoding	Missing indicator treated as feature
Systemic target at onset	Categorical	50	33	One-Hot Encoding	Missing indicator treated as feature
Pain (NRS) at onset	Numeric	45	30	Missing Values Imputed	Missing indicator treated as feature, Imputed value: 3
Disease activity (NRS) at onset	Numeric	45	30	Missing Values Imputed	Imputed value: 2
CASPAR classification at onset	Numeric	40	26	Missing Values Imputed	Missing indicator treated as feature, Imputed value: 1
CASPAR score at onset	Numeric	40	26	Missing Values Imputed	Imputed value: 3
Pain change over 24 weeks	Categorical	39	26	One-Hot Encoding	Missing indicator treated as feature
Therapy change differential	Categorical	18	12	One-Hot Encoding	Missing indicator treated as feature
Therapy change binary	Numeric	18	12	Missing Values Imputed	Missing indicator treated as feature, Imputed value: 1
Occupation	Categorical	2	1	One-Hot Encoding	Missing indicator treated as feature
Pruritus change over 24 weeks	Categorical	2	1	One-Hot Encoding	Missing indicator treated as feature
Obesity at onset	Numeric	2	1	Missing Values Imputed	Missing indicator treated as feature, Imputed value: 0
DLQI classification change over 24 weeks	Categorical	1	1	One-Hot Encoding	Missing indicator treated as feature
HADS-A classification change over 24 weeks	Categorical	1	1	One-Hot Encoding	Missing indicator treated as feature
HADS-D classification change over 24 weeks	Categorical	1	1	One-Hot Encoding	Missing indicator treated as feature
Body height at onset	Numeric	1	1	Missing Values Imputed	Missing indicator treated as feature, Imputed value: 173
Body weight at onset	Numeric	1	1	Missing Values Imputed	Imputed value: 86
BMI	Numeric	1	1	Missing Values Imputed	Imputed value: 27.7

Alcohol	Numeric	1	1	Missing Values Imputed	Missing indicator treated as feature, Imputed value: 1
Sports	Numeric	1	1	Missing Values Imputed	Missing indicator treated as feature, Imputed value: 0
Gender	Categorical	0	0	One-Hot Encoding	Missing values ignored
Physical activity at onset	Categorical	0	0	One-Hot Encoding	Missing values ignored
DLQI classification at onset	Categorical	0	0	One-Hot Encoding	Missing values ignored
HADS-A classification at onset	Categorical	0	0	One-Hot Encoding	Missing values ignored
HADS-D classification at onset	Categorical	0	0	One-Hot Encoding	Missing values ignored
Topical therapy duration over 24 weeks	Categorical	0	0	One-Hot Encoding	Missing values ignored
app used	Categorical	0	0	One-Hot Encoding	Missing values ignored
Patient_ID	Categorical	0	0	One-Hot Encoding	Missing values ignored
Age	Numeric	0	0	Missing Values Imputed	Imputed value: 52
Smoking	Numeric	0	0	Missing Values Imputed	Imputed value: 0
Diagnosed depression at onset	Numeric	0	0	Missing Values Imputed	Imputed value: 0
Diagnosed arterial hypertension at onset	Numeric	0	0	Missing Values Imputed	Imputed value: 0
No diagnosed pre-existing illness at onset	Numeric	0	0	Missing Values Imputed	Imputed value: 0
Diagnosed coronary heart disease at onset	Numeric	0	0	Missing Values Imputed	Imputed value: 0
Diagnosed metabolic disease at onset	Numeric	0	0	Missing Values Imputed	Imputed value: 0
DLQI classification at onset	Numeric	0	0	Missing Values Imputed	Imputed value: 4
HADS-A score at onset	Numeric	0	0	Missing Values Imputed	Imputed value: 6
HADS-D score at onset	Numeric	0	0	Missing Values Imputed	Imputed value: 4
Therapy with TNF-α inhibitors at onset	Numeric	0	0	Missing Values Imputed	Imputed value: 0
Therapy with IL-17 Inhibitors at onset	Numeric	0	0	Missing Values Imputed	Imputed value: 0
Therapy with IL-12/23 Inhibitors at onset	Numeric	0	0	Missing Values Imputed	Imputed value: 0
Therapy with IL-23 Inhibitors at onset	Numeric	0	0	Missing Values Imputed	Imputed value: 0
Therapy with csDMARDs at onset	Numeric	0	0	Missing Values Imputed	Imputed value: 0
Therapy with others then b-/csDMARDs	Numeric	0	0	Missing Values Imputed	Imputed value: 0
Topical therapy at onset	Numeric	0	0	Missing Values Imputed	Imputed value: 0
Pruritus (NRS) at onset	Numeric	0	0	Missing Values Imputed	Imputed value: 1
PASI score at onset	Numeric	0	0	Missing Values Imputed	Imputed value: 2.4

Eureqa Classifier (Default Search 3000 Generations)

Feature Name	Var Type	Missing Count	Missing Percentage	Imputation Name	Imputation Description
Diagnosed other disease at onset	Categorical	93	62	One-Hot Encoding	Missing indicator treated as feature
BASDAI score at onset	Numeric	77	51	Missing Values Imputed	Missing indicator treated as feature, Imputed value: 2.8
BASDAI classification at onset	Numeric	77	51	Missing Values Imputed	Imputed value: 0
Systemic treatment at onset	Categorical	50	33	One-Hot Encoding	Missing indicator treated as feature
Systemic target at onset	Categorical	50	33	One-Hot Encoding	Missing indicator treated as feature
Pain (NRS) at onset	Numeric	45	30	Missing Values Imputed	Missing indicator treated as feature, Imputed value: 3
Disease activity (NRS) at onset	Numeric	45	30	Missing Values Imputed	Imputed value: 2
CASPAR classification at onset	Numeric	40	26	Missing Values Imputed	Missing indicator treated as feature, Imputed value: 1
CASPAR score at onset	Numeric	40	26	Missing Values Imputed	Imputed value: 3
Pain change over 24 weeks	Categorical	39	26	One-Hot Encoding	Missing indicator treated as feature
Therapy change differential	Categorical	18	12	One-Hot Encoding	Missing indicator treated as feature
Therapy change binary	Numeric	18	12	Missing Values Imputed	Missing indicator treated as feature, Imputed value: 1
Occupation	Categorical	2	1	One-Hot Encoding	Missing values treated as infrequent
Pruritus change over 24 weeks	Categorical	2	1	One-Hot Encoding	Missing values treated as infrequent
Obesity at onset	Numeric	2	1	Missing Values Imputed	Missing indicator treated as feature, Imputed value: 0
DLQI classification change over 24 weeks	Categorical	1	1	One-Hot Encoding	Missing values treated as infrequent
HADS-A classification change over 24 weeks	Categorical	1	1	One-Hot Encoding	Missing values treated as infrequent
HADS-D classification change over 24 weeks	Categorical	1	1	One-Hot Encoding	Missing values treated as infrequent
Body height at onset	Numeric	1	1	Missing Values Imputed	Missing indicator treated as feature, Imputed value: 173
Body weight at onset	Numeric	1	1	Missing Values Imputed	Imputed value: 86
BMI	Numeric	1	1	Missing Values Imputed	Imputed value: 27.7
Alcohol	Numeric	1	1	Missing Values Imputed	Missing indicator treated as feature, Imputed value: 1

Sports	Numeric	1	1	Missing Values Imputed	Missing indicator treated as feature, Imputed value: 0
Patient_ID	Categorical	0	0	One-Hot Encoding	Missing values treated as infrequent
Gender	Categorical	0	0	One-Hot Encoding	Missing values ignored
Physical activity at onset	Categorical	0	0	One-Hot Encoding	Missing values ignored
DLQI classification at onset	Categorical	0	0	One-Hot Encoding	Missing values treated as infrequent
HADS-A classification at onset	Categorical	0	0	One-Hot Encoding	Missing values ignored
HADS-D classification at onset	Categorical	0	0	One-Hot Encoding	Missing values ignored
Topical therapy duration over 24 weeks	Categorical	0	0	One-Hot Encoding	Missing values ignored
app used	Categorical	0	0	One-Hot Encoding	Missing values ignored
Age	Numeric	0	0	Missing Values Imputed	Imputed value: 52
Smoking	Numeric	0	0	Missing Values Imputed	Imputed value: 0
Diagnosed depression at onset	Numeric	0	0	Missing Values Imputed	Imputed value: 0
Diagnosed arterial hypertension at onset	Numeric	0	0	Missing Values Imputed	Imputed value: 0
No diagnosed pre-existing illness at onset	Numeric	0	0	Missing Values Imputed	Imputed value: 0
Diagnosed coronary heart disease at onset	Numeric	0	0	Missing Values Imputed	Imputed value: 0
Diagnosed metabolic disease at onset	Numeric	0	0	Missing Values Imputed	Imputed value: 0
DLQI classification at onset	Numeric	0	0	Missing Values Imputed	Imputed value: 4
HADS-A score at onset	Numeric	0	0	Missing Values Imputed	Imputed value: 6
HADS-D score at onset	Numeric	0	0	Missing Values Imputed	Imputed value: 4
Therapy with TNF-α inhibitors at onset	Numeric	0	0	Missing Values Imputed	Imputed value: 0
Therapy with IL-17 Inhibitors at onset	Numeric	0	0	Missing Values Imputed	Imputed value: 0
Therapy with IL-12/23 Inhibitors at onset	Numeric	0	0	Missing Values Imputed	Imputed value: 0
Therapy with IL-23 Inhibitors at onset	Numeric	0	0	Missing Values Imputed	Imputed value: 0
Therapy with csDMARDs at onset	Numeric	0	0	Missing Values Imputed	Imputed value: 0
Therapy with others then b-/csDMARDs	Numeric	0	0	Missing Values Imputed	Imputed value: 0
Topical therapy at onset	Numeric	0	0	Missing Values Imputed	Imputed value: 0
Pruritus (NRS) at onset	Numeric	0	0	Missing Values Imputed	Imputed value: 1
PASI score at onset	Numeric	0	0	Missing Values Imputed	Imputed value: 2.4

Target 3: AVG Blender (Eureqa Generalized Additive Model Classifier (40 Generations), eXtreme Gradient Boosted Trees Classifier with Early Stopping, Dropout Additive Regression Trees Classifier (15 leaves))

Eureqa Generalized Additive Model Classifier (40 Generations)

Feature Name	Var Type	Missing Count	Missing Percentage	Imputation Name	Imputation Description
Pain (NRS) at onset	Numeric	2	2	Missing Values Imputed	Missing indicator treated as feature, Imputed value: 2
Body height at onset	Numeric	1	1	Missing Values Imputed	Missing indicator treated as feature, Imputed value: 173
Pruritus (NRS) at onset	Numeric	1	1	Missing Values Imputed	Missing indicator treated as feature, Imputed value: 2
Disease activity (NRS) at onset	Numeric	1	1	Missing Values Imputed	Missing indicator treated as feature, Imputed value: 2
Occupation	Categorical	0	0	One-Hot Encoding	Missing values treated as infrequent
DLQI classification at onset	Numeric	0	0	Missing Values Imputed	Imputed value: 6
HADS-A score at onset	Numeric	0	0	Missing Values Imputed	Imputed value: 8
HADS-D score at onset	Numeric	0	0	Missing Values Imputed	Imputed value: 6
PASI score at onset	Numeric	0	0	Missing Values Imputed	Imputed value: 3

eXtreme Gradient Boosted Trees Classifier with Early Stopping

Feature Name	Var Type	Missing Count	Missing Percentage	Imputation Name	Imputation Description
Pain (NRS) at onset	Numeric	2	2	Missing Values Imputed	Missing indicator treated as feature, Imputed value: 2
Body height at onset	Numeric	1	1	Missing Values Imputed	Missing indicator treated as feature, Imputed value: 173
Pruritus (NRS) at onset	Numeric	1	1	Missing Values Imputed	Missing indicator treated as feature, Imputed value: 2
Disease activity (NRS) at onset	Numeric	1	1	Missing Values Imputed	Missing indicator treated as feature, Imputed value: 2
Occupation	Categorical	0	0	Ordinal encoding of categorical variables	Imputed value: -2
DLQI classification at onset	Numeric	0	0	Missing Values Imputed	Imputed value: 6
HADS-A score at onset	Numeric	0	0	Missing Values Imputed	Imputed value: 8
HADS-D score at onset	Numeric	0	0	Missing Values Imputed	Imputed value: 6
PASI score at onset	Numeric	0	0	Missing Values Imputed	Imputed value: 3

Dropout Additive Regression Trees Classifier (15 leaves)

Feature Name	Var Type	Missing Count	Missing Percentage	Imputation Name	Imputation Description
Pain (NRS) at onset	Numeric	2	2	Missing Values Imputed	Missing indicator treated as feature, Imputed value: 2
Body height at onset	Numeric	1	1	Missing Values Imputed	Missing indicator treated as feature, Imputed value: 173
Pruritus (NRS) at onset	Numeric	1	1	Missing Values Imputed	Missing indicator treated as feature, Imputed value: 2
Disease activity (NRS) at onset	Numeric	1	1	Missing Values Imputed	Missing indicator treated as feature, Imputed value: 2
Occupation	Categorical	0	0	Ordinal encoding of categorical variables	Imputed value: -2
DLQI classification at onset	Numeric	0	0	Missing Values Imputed	Imputed value: 6
HADS-A score at onset	Numeric	0	0	Missing Values Imputed	Imputed value: 8
HADS-D score at onset	Numeric	0	0	Missing Values Imputed	Imputed value: 6
PASI score at onset	Numeric	0	0	Missing Values Imputed	Imputed value: 3
