# Supplementary material for: Using Automated Machine Learning to Predict Necessary Upcoming Therapy Changes in Patients With Psoriasis Vulgaris and Psoriatic Arthritis and Uncover New Influences on Disease Progression: Retrospective Study
Source: JMIR Form Res. 2024 Jun 27;8:e55855. doi: 10.2196/55855 (PMC11240079; doi:10.2196/55855)
Supplement: Multimedia Appendix 5 [file formative_v8i1e55855_app5.pdf]

Multimedia Appendix 5

Reduced feature list used for Target 1.1: "Therapy change at 24 weeks follow-up"

Age
BMI
Occupation
Smoking
Alcohol
Pre-existing illnesses
Sports
Physical activity at onset
DLQI classification at onset
HADS-A classification at onset
HADS-D classification at onset
CASPAR score at onset
BASDAI score at onset
Systemical treatment at onset
Topical therapy at onset
Topical therapy duration over 24 weeks
Pruritus change over 24 weeks
PASI change over 24 weeks
DLQI classification change over 24 weeks
HADS-A classification change over 24 weeks
HADS-D classification change over 24 weeks

This table details the secondary dataset features used in the AutoML platform to predict therapy changes in psoriasis patients at 24-week follow-up. The feature set includes patient demographics, lifestyle factors (e.g. smoking, alcohol consumption), preexisting conditions, physical activity levels and clinical scores (e.g. DLQI, HADS-A/D, CASPAR, BASDAI) at baseline and changes over 24 weeks. Treatment data include systemic and topical therapies, including duration and changes in pruritus and PASI scores.
