# Supplementary material for: Using Automated Machine Learning to Predict Necessary Upcoming Therapy Changes in Patients With Psoriasis Vulgaris and Psoriatic Arthritis and Uncover New Influences on Disease Progression: Retrospective Study
Source: JMIR Form Res. 2024 Jun 27;8:e55855. doi: 10.2196/55855 (PMC11240079; doi:10.2196/55855)
Supplement: Multimedia Appendix 6 [file formative_v8i1e55855_app6.pdf]

Multimedia Appendix 6

Reduced feature list used for Target 1.2: "Therapy change prediction (only onset features)"

Gender
Age
Bodyheight
Bodyweight
BMI
Occupation
Smoking
Alcohol
Diagnosed obesity
Diagnosed depression
Diagnosed hypertension
No pre-existing illnesses
Diagnosed coronary heart disease
Diagnosed metabolic disease
Other pre existing illnesses
Sports
Physical activity at onset
DLQI score at onset
HADS-A score at onset
HADS-D score at onset
DLQI classification at onset
HADS-A classification at onset
HADS-D classification at onset
CASPAR classification at onset
CASPAR score at onset
BASDAI score at onset
BASDAI classification at onset
Systemical treatment at onset
Systemical target at onset
Therapy with TNF-α inhibitors at onset
Therapy with IL-17 Inhibitors at onset
Therapy with IL-23 inhibitors at onset
Therapy with IL-12/23 inhibitor at onset
Therapy with csDMARDs at onset
Therapy with others then b-/csDMARDs
Topical therapy at onset
Pain (NRS) at onset
Pruritus (NRS) at onset
Disease activity (NRS) at onset
PASI score at onset

The feature list includes initial patient characteristics and clinical assessments to predict therapy adjustments without considering longitudinal changes. Included features include basic demographics (gender, age, height, weight, BMI), lifestyle choices (smoking, alcohol consumption) and detailed medical history, including diagnosed conditions such as obesity, depression, hypertension, coronary heart disease, metabolic disease and other preexisting conditions. Physical activity and sports participation at the time of enrollment are also taken into account. Clinical assessments at baseline include the Dermatology Life Quality Index (DLQI) score and classification, the Hospital Anxiety and Depression Scale (HADS) score and classification for anxiety (A) and depression (D), the Classification Criteria for Psoriatic Arthritis (CASPAR) score and classification, and the Bath Ankylosing Spondylitis Disease Activity Index (BASDAI) score and classification. Treatment specifics at baseline include systemic therapies, including TNF-α, IL-17, IL-23, IL-12/23 inhibitors, conventional synthetic disease-modifying antirheumatic drugs (csDMARDs), other drugs and topical therapies. Patient-reported outcomes at baseline such as pain, pruritus and disease activity as measured by the Numerical Rating Scale (NRS) and Psoriasis Area and Severity Index (PASI) score are included to provide a comprehensive snapshot for predictive modeling in the early stages of treatment.
