# Supplementary material for: Using Automated Machine Learning to Predict Necessary Upcoming Therapy Changes in Patients With Psoriasis Vulgaris and Psoriatic Arthritis and Uncover New Influences on Disease Progression: Retrospective Study
Source: JMIR Form Res. 2024 Jun 27;8:e55855. doi: 10.2196/55855 (PMC11240079; doi:10.2196/55855)
Supplement: Multimedia Appendix 7 [file formative_v8i1e55855_app7.pdf]

Multimedia Appendix 7

Reduced feature list used for Target 2: "PASI change after 24 weeks"

Patient ID
Gender
Age
Body height at onset
Body weight at onset
BMI at onset
Occupation
Smoking
Alcohol
Obesity at onset
Diagnosed depression at onset
Diagnosed arterial hypertension at onset
No diagnosed pre-existing illness at onset
Diagnosed coronary heart disease at onset
Diagnosed metabolic disease at onset
Diagnosed other disease at onset
Sports
Physical activity at onset
DLQI score at onset
HADS-A score at onset
HADS-D score at onset
DLQI classification at onset
HADS-A classification at onset
HADS-D classification at onset
CASPAR classification at onset
CASPAR score at onset
BASDAI score at onset
BASDAI classification at onset
Systemic treatment at onset
Systemic target at onset
Therapy with TNF-α inhibitors at onset
Therapy with IL-17 Inhibitors at onset
Therapy with IL-12 /23 inhibitor at onset
Therapy with IL-23 inhibitors at onset
Therapy with csDMARDs at onset
Therapy with others then b-/csDMARDs
Therapy change differential
Therapy change binary
Topical therapy at onset
Topical therapy duration over 24 weeks
Pain (NRS) at onset
Pain change over 24 weeks
Pruritus (NRS) at onset
Pruritus change over 24 weeks

Disease activity (NRS) at onset
PASI score at onset
DLQI classification change over 24 weeks
HADS-A classification change over 24 weeks
HADS-D classification change over 24 weeks
App used

This appendix includes baseline patient demographics, lifestyle factors, comorbidities and clinical scores (e.g. DLQI, HADS, CASPAR, BASDAI). Treatment data include systemic and topical therapies at baseline, as well as changes in pain, pruritus and clinical classifications over 24 weeks. This reduced feature list aims to enable accurate prediction of PASI score changes with AutoML.
