# Supplementary material for: Using Automated Machine Learning to Predict Necessary Upcoming Therapy Changes in Patients With Psoriasis Vulgaris and Psoriatic Arthritis and Uncover New Influences on Disease Progression: Retrospective Study
Source: JMIR Form Res. 2024 Jun 27;8:e55855. doi: 10.2196/55855 (PMC11240079; doi:10.2196/55855)
Supplement: Multimedia Appendix 8 [file formative_v8i1e55855_app8.pdf]

Multimedia Appendix 8

Reduced feature list used for Target 3: "BASDAI classification at onset"

Body height at onset
Occupation
DLQI score at onset
HADS-A score at onset
HADS-D score at onset
Pain (NRS) at onset
Pruritus (NRS) at onset
Disease activity (NRS) at onset
PASI score at onset

This feature list focuses on initial clinical assessments and patient characteristics to model the Bath Ankylosing Spondylitis Disease Activity Index (BASDAI) classification. Features include patient height at baseline and occupational status. Clinical measures collected at baseline include the Dermatology Life Quality Index (DLQI) score, Hospital Anxiety and Depression Scale (HADS) scores for anxiety (A) and depression (D), and patient-reported outcomes for pain, pruritus and overall disease activity, all measured using the Numerical Rating Scale (NRS). The Psoriasis Area and Severity Index (PASI) score at baseline is also included.
