# Supplementary material for: Using Automated Machine Learning to Predict Necessary Upcoming Therapy Changes in Patients With Psoriasis Vulgaris and Psoriatic Arthritis and Uncover New Influences on Disease Progression: Retrospective Study
Source: JMIR Form Res. 2024 Jun 27;8:e55855. doi: 10.2196/55855 (PMC11240079; doi:10.2196/55855)
Supplement: Multimedia Appendix 9 [file formative_v8i1e55855_app9.pdf]

Multimedia Appendix 9

Hyperparameter optimisation strategies for selected ML models

This appendix presents the configurations tested for various parameters such as tree construction methods, learning rates, regularization techniques, and feature encoding strategies. The optimization process aims to fine-tune model performance by adjusting parameters. This systematic approach to hyperparameter tuning improves the predictive accuracy and generalisability of the models.

Hyperparameters selections

Target 1.1: eXtreme Gradient Boosted Trees Classifier with Early Stopping

Ordinal scale converter of categorical features

Type	Name	Description	Best Searched
select	add_cols_metadata	If specified, add -cols to metadata. values: [False, True]	False
select	add_maps_metadata	If specified, add -maps to metadata. values: [False, True]	False
multi	card_max	Maximum number of categorical feature levels allowed. If None, a feature with any number of levels is allowed. values: {'int': [1, 9999999], 'select': None}	None
select	method	Method used in the encoding. None: uses random_scale. random: random ordering of levels, lex: lexicographical ordering by category level names, freq: frequency ordering from least frequent to most frequent, resp: response ordering. values: ['None', 'random', 'lex', 'freq', 'resp']	freq
int	min_support	Minimum number of levels required for a category to be represented on the ordinal scale. If a category level count is below the minimum, it will be grouped with other small cardinality levels or encoded as a missing value, depending on the value of other_category. values: [1, 99999]	5
int	offset	Shift the ordinal scale of ordinal encoder values: [0, 99999]	0
bool	other_category	If True, small cardinality values are mapped to a dedicated value (-1), otherwise they are encoded as missing values (-2). values: [False, True]	True
bool	random_scale	Applies if method is None. If random_scale is True, random ordering is used for the ordinal scale. If it is False, lexicographical ordering is used. values: [False, True]	True
int	seed	The RNG seed. values: [0, 99999]	1234

Type	Name	Description	Best Searched
bool	scale_small	True if small values (range of the numeric variable is <= 1) are to be scaled. values: [False, True]	True
int	threshold	Minimum number of required finite elements in a column to impute the data onto NaNs and INFs. values: [1, 99999]	10

Prediction Model Parameters (eXtreme Gradient Boosted Trees Classifier with early stopping)

Type	Name	Description	Best Searched
select	base_margin_initialize	If True, the intercept is initialized to the log odds of the target. values: [False, True]	False
int	class_count	Number of target classes (multiclass only). values: [0, MAX_TARGET_CLASS_COUNT]	None
floatgrid	colsample_bylevel	Subsample of the features before each split in a tree. values: [0.1,1]	1.0
floatgrid	colsample_bytree	Subsample ratio of columns when constructing each tree. By default, the value of colsample_bytree for XGBoost classes is 1.0. However, based on the training data, DataRobot may choose a different initial value for this parameter. values: [0,1]	0.5
int	interval	Interval for early stopping. Once the model has hit “smooth_interval” iterations, the early stopping logic checks for errors increasing at a rate of “interval” iterations in a row. For example, if smooth_interval=200 and interval=10, XGBoost will run at least 200 iterations, and will early stop if the moving average of loss for the last 200 iterations increases for 10 iterations in a row. Higher values will make XGBoost run longer. values: [2, 500]	10
floatgrid	learning_rate	Shrinks the contribution of each tree by learning_rate. There is a trade-off between learning_rate (lr) and n_estimators(n). values: [5e-4,1]	0.05
select	loss	Loss function to be used during optimization. ‘deviance’ refers to deviance (= logistic regression) for classification with probabilistic outputs. values: ['deviance', 'softprob']	deviance
int	max_bin	Used when tree_method is set to ‘hist’. Maximum number of discrete bins to bucket continuous features. Increasing this number improves the optimality of splits at the cost of higher computation time. values: [16, 2048]	256
floatgrid	max_delta_step	Maximum delta step allowed for each tree’s weight estimation. If the value is set to 0, there is no constraint. Setting to a positive value makes the update step more conservative. Usually this parameter is not needed, but it might help in logistic regression when class is extremely imbalanced. Setting it to a value of 1-10 might help control the delta step update. values: [0,100]	0.0
intgrid	max_depth	Maximum depth of the individual regression estimators. The maximum depth limits the number of nodes in the	10

		tree. Tune this parameter for optimal performance; the best value depends on the interaction of the input variables. The deeper the tree, the more variable interactions the model can capture. For frozen models that have larger sample sizes than the parent model, the max_depth value is increased to retain similar accuracy. values: [1, 16]	
floatgrid	min_child_weight	Minimum sum of instance weight (hessian) needed in a child. If the tree partition step results in a leaf node with the sum of instance weight less than min_child_weight, the building process will give up further partitioning. In linear regression mode, this simply corresponds to the minimum number of instances needed to be in each node. The larger the value, the more conservative the algorithm will be. values: [0.01,float(1e5)]	1.0
floatgrid	min_split_loss	Minimum loss reduction required to make a further partition on a leaf node of the tree. The larger the value, the more conservative the algorithm will be. values: [0,1e5]	0.01
float	missing_value	Float value that should be treated as a missing value. When mono_up or mono_down is set, missing value will be set to -9999.0. values: [float(-1e5),float(1e5)]	-9999.0
string	mono_down	ID of the featurelist that defines the set of features with a monotonically decreasing relationship to the target.	no
string	mono_up	ID of the featurelist that defines the set of features with a monotonically increasing relationship to the target.	no
int	n_estimators	Number of boosting stages to perform. Gradient boosting is fairly robust to overfitting, so a larger number usually results in better performance. values: [1,20000]	50
intgrid	num_parallel_tree	Number of parallel trees created in each boosting stage. When this value is greater than 1, the model becomes a gradient-boosted random forest with (num_parallel_tree * n_estimators) trees. values: [1,16]	1
intgrid	random_state	Seed used in the random number generator 'values': [0, int(1e9)]	1234
multi	reg_alpha	L1 regularization term on weights; increasing this value will make the model more conservative. values: {'floatgrid': [0, 1e6], 'select': ['auto']}	0.0
multi	reg_lambda	L2 regularization term on weights. Increasing this value will make the model more conservative. values: {'floatgrid': [0, 1e6], 'select': ['auto']}	1.0
float	scale_pos_weight	Scaling factor for examples in the positive class. values: [0,float(1e9)]	1.0
int	smooth_interval	Moving average interval for early stopping. Loss over the last n intervals is averaged for determining early stopping. For example, if smooth_interval=200, XGBoost will not stop early until it hits at least 200 iterations. After 200 iterations, it will use a moving average of loss over the last 200 iterations to determine early stopping. This helps remove noise in the	200

		loss function. Higher values will make XGBoost run longer. values: [2, 1000]	
floatgrid	subsample	Subsample ratio of the training instance. Setting it to 0.5 means that XGBoost randomly collected half of the data instances to grow trees, which will prevent overfitting.	1.0
select	tree_method	Tree construction algorithm to use. ‘auto’: Heuristic to choose the faster algortihm. For small to medium datasets (<4M rows), exact greedy will be used. For large datasets (>=4M rows), approximate algorithm will be used. ‘exact’:Exact greedy algorithm. ‘approx’:Approximate greedy algorithm using sketching and histogram. ‘hist’:Fast histogram-optimized approximate greedy algorithm. It uses some performance improvements (e.g., bin caching). values: ['auto', 'exact', 'approx', 'hist']	auto

Target 1.2: AVG Blender (Gradient Boosted Trees Classifier, ExtraTrees Classifier (Gini), Eureqa Generalized Additive Model Classifier (1000 Generations))

Gradient Boosting Trees Classifier

Ordinal scale converter of categorical features

Type	Name	Description	Best Searched
select	add_cols_metadata	If specified, add -cols to metadata. values: [False, True]	False
select	add_maps_metadata	If specified, add -maps to metadata. values: [False, True]	False
multi	card_max	Maximum number of categorical feature levels allowed. If None, a feature with any number of levels is allowed. values: {'int': [1, 9999999], 'select': None}	None
select	method	Method used in the encoding. None: uses random_scale. random: random ordering of levels, lex: lexicographical ordering by category level names, freq: frequency ordering from least frequent to most frequent, resp: response ordering. values: ['None', 'random', 'lex', 'freq', 'resp']	freq
int	min_support	Minimum number of levels required for a category to be represented on the ordinal scale. If a category level count is below the minimum, it will be grouped with other small cardinality levels or encoded as a missing value, depending on the value of other_category. values: [1, 99999]	5
int	offset	Shift the ordinal scale of ordinal encoder values: [0, 99999]	0
bool	other_category	If True, small cardinality values are mapped to a dedicated value (-1), otherwise they are encoded as missing values (-2). values: [False, True]	True
bool	random_scale	Applies if method is None. If random_scale is True, random ordering is used for the ordinal scale. If it is False,	True

		lexicographical ordering is used. values: [False, True]	
int	seed	The RNG seed. values: [0, 99999]	1234

Median Value-Based Numeric Imputation (V2 with quick median algorithm)

Type	Name	Description	Best Searched
bool	scale_small	True if small values (range of the numeric variable is <= 1) are to be scaled. values: [False, True]	False
int	threshold	Minimum number of required finite elements in a column to impute the data onto NaNs and INFs. values: [1, 99999]	10

Prediction Model Parameters (Plain) Gradient Boosting Classifier

Type	Name	Description	Best Searched
floatgrid	learning_rate	Shrinks the contribution of each tree by learning_rate. There is a trade-off between learning_rate (lr) and n_estimators(n). values: [5e-4,1]	0.005
string	loss	The loss function to be optimized. ‘deviance’ refers to deviance (= logistic regression) for classification with probabilistic outputs. values: ['deviance']	deviance
intgrid	max_depth	maximum depth of the individual regression estimators. The maximum depth limits the number of nodes in the tree. Tune this parameter for best performance; the best value depends on the interaction of the input variables. Deeper the tree the more variable interactions the model can capture. Ignored if max_leaf_nodes is not None. values: {'intgrid':[1, 16]	3
string	max_features	The number of features to consider when looking for the best split: - If int, then consider max_features features at each split. - If float, then max_features is a percentage and int(max_features * n_features) features are considered at each split. - If “auto”, then max_features=sqrt(n_features). - If “sqrt”, then max_features=sqrt(n_features). - If “log2”, then max_features=log2(n_features). - If None, then max_features=n_features. Choosing max_features < n_features leads to a reduction of variance and an increase in bias. Note: the search for a split does not stop until at least one valid partition of the node samples is found, even if it requires to effectively inspect more than max_features features. values: {'select':['auto','sqrt','log2','None'], 'intgrid':[1,int(1e8)], 'floatgrid':[1e-06,1.]}	0.2
intgrid	max_leaf_nodes	Grow trees with max_leaf_nodes in best-first fashion. Best nodes are defined as relative reduction in impurity. If None then unlimited number of leaf nodes. If not None then max_depth will be ignored. values: {'intgrid':[2, 99]	None
multi	min_samples_leaf	The minimum number of samples required to be at a leaf node. values: {'intgrid': [1, int(1e5)], 'select': ['heuristic']}	1

multi	min_samples_split	The minimum number of samples required to split an internal node. values: {'intgrid': [1, int(1e5)], 'select': ['heuristic']}	3
int	n_estimators	The number of boosting stages to perform. Gradient boosting is fairly robust to over-fitting so a large number usually results in better performance. values:[1,20000]	500
int	random_state	If int, random_state is the seed used by the random number generator; If RandomState instance, random_state is the random number generator; If None, the random number generator is the RandomState instance used by np.random. values: [0, int(1e9)]	1234
floatgrid	subsample	The fraction of samples to be used for fitting the individual base learners. If smaller than 1.0 this results in Stochastic Gradient Boosting. subsample interacts with the parameter n_estimators. Choosing subsample < 1.0 leads to a reduction of variance and an increase in bias. values: [0.01,1]	1.0

ExtraTrees Classifier (Gini)

One-Hot Encoding Task

Type	Name	Description	Best Searched
int	card_max	An integer that specifies the maximum number of unique values. values: [1, 99999]	2
int	card_min	An integer that specifies the minimum number of unique values. values: [1, 99999]	1
bool	drop_cols	drop_cols, If True, drop last level of each feature values: [False, True]	False
select	flag	flag, If all, add highcat-cols to metadata values: ['None', 'all']	None
int	max_features	If the total number of categories created across all features exceeds this value, the top max_features most frequent categories will persist. All others will be either thrown out or grouped. A value of None disables the limit. values: [1, 999999]	0.3
int	min_support	The minimum number of records for a category to be represented in one hot encoding. If a category has fewer counts it will be grouped with other small cardinality values. values: [1, 99999]	10

CredL2_1b1 task

Type	Name	Description	Best Searched
select	ElasticNet distribution	distribution to pass to ElasticNet model values: ['squared', 'poisson', 'gamma', 'tweedie']	squared
bool	ElasticNet fit_tweedie_p	fit_tweedie_p to pass to ElasticNet model values: [False, True]	False
floatgrid	ElasticNet mixing parameter	Mixing parameter grid to pass to ElasticNet model values: [0, 1]	0.5

multi	ElasticNet regularization	Regularization grid to pass to ElasticNet model values: {'floatgrid': [1e-10, 1e10], 'select': ['auto']}	auto
float	ElasticNet tweedie_p	tweedie power to pass to ElasticNet model values': [1.0, 2.0]	1.5
multi	Logistic regr. regularization	Regularization grid to pass to Logistic regression model values: {'floatgrid': [1e-10, 1e10], 'select': ['auto']}	auto
multi	Ridge regularization	Regularization grid to pass to Ridge regression model values: {'floatgrid': [1e-10, 1e10], 'select': ['auto']}	auto
floatgrid	SGD regularization	Regularization grid to pass to SGD models values: {'floatgrid': [1e-10, 1e10], 'select': ['auto']}	auto
int	card_max	Maximum number of values a category is allowed to have values: [1, 99999999]	10000
int	card_min	Minimum number of unique values to determine credibility values: [0, 99999]	3
bool	fit_intercept	whether to calculate the intercept for this model. If set to False, no intercept will be used in calculations	True
float	intercept_scaling	the scaling value for the intercept values: [1e-10, 1e10]	1.0
int	num_folds	Number of folds for the cross-validation values: [1, 99999]	5
select	regression_type	sets regression type values: ['Not specified', 'Regression', 'Binary']	None
int	small_count	Below this number the variables will be grouped in a small_count category values: [1, 99999]	5
bool	two_ways	sets if 2-ways interactions should be computed values: [False, True]	False
int	two_ways_max	Maximum number of features for which 2-ways credibility are computed. The selected features are the most informative features. values: [2, 99999]	5
bool	use_enet	sets if ElasticNet should be used values: [False, True]	True
bool	use_sgd	sets if SGD should be used values: [False, True]	False

Median Value-Based Numeric Imputation (V2 with quick median algorithm)

Type	Name	Description	Best Searched
bool	scale_small	True if small values (range of the numeric variable is <= 1) are to be scaled. values: [False, True]	False
int	threshold	Minimum number of required finite elements in a column to impute the data onto NaNs and INFs. values: [1, 99999]	10

Feature selection for Ratios/Differences

Type	Name	Description	Best Searched

int	FeatureSelection: max_features	The maximum number of features to include. ``values: {'int': [1, int(1e10)], 'select': ['no_limit']}`	50
select	FeatureSelection: method	Method used to select features values: ['per_variable', 'cumulative', 'no_selection']	per_variable
int	FeatureSelection: min_features	The minimum number of features to include. values: [1, int(1e5)]	1
float	FeatureSelection: threshold	The threshold used to select features. For cumulative method, threshold is typically close to 1 in order to retain more signal (ex:0.98). For per_variable method, threshold would be close to 0 in order to discard only features with low signal. values: [0.0001, 1]	0.001

Greedy Search for differences

Type	Name	Description	Best Searched
float	maximum scale ratio	Defines the highest scale ratio between 2 raw features mean to test the difference as a new feature values: [1e-06,10]	3.0
int	min samples split	Defines the minimum number of samples required to split an internal node in the tree used to test difference features. values: [1, 10000]	10
float	minimum correlation	Defines the minimum correlation between 2 raw features to test the difference as a new feature values: [0, 0.99]	0.3
float	minimum improvement	Defines the minimum improvment vs raw features only values: [0, 0.99]	0.01
int	seed	Defines the random seed for the cross validation partition values: [0, int(1e20)]	1234
float	test size	test_size of holdout set to measure accuracy performance values: [0.01, 0.99]	0.2

Greedy Search for ratios

Type	Name	Description	Best Searched
select	distribution	The distribution to use values: ['Gaussian', 'Poisson', 'Bernoulli', 'Gamma', 'Tweedie']	Bernoulli
float	minimum correlation	Defines the minimum correlation between 2 raw features to test the ratio as a new feature values: [0, 0.99]	0.3
float	minimum improvement	Defines the minimum improvment vs raws features only values: [0, 0.99]	0.01
int	seed	Defines the random seed for the cross validation partition values: [0, int(1e20)]	1234
select	test size	test_size of holdout set to measure accuracy performance values: [0.01, 0.99]	0.2

Feature Selection for Dimensionality Reduction

Type	Name	Description	Best Searched

int	FeatureSelection: max_features	The maximum number of features to include. ``values: {'int': [1, int(1e10)], 'select': ['no_limit']}`	no_limit
select	FeatureSelection: method	Method used to select features values: ['per_variable', 'cumulative', 'no_selection']	per_variable
int	FeatureSelection: min_features	The minimum number of features to include. values: [1, int(1e5)]	50
float	FeatureSelection: threshold	The threshold used to select features. For cumulative method, threshold is typically close to 1 in order to retain more signal (ex:0.98). For per_variable method, threshold would be close to 0 in order to discard only features with low signal. values: [0.0001, 1]	0.001

Prediction Model Parameters Random Forest Classifier

Type	Name	Description	Best Searched
bool	balanced_bootstrap	Whether the sampling probability per class should be inverse to the class frequency values: [False,True]	False
bool	bootstrap	Whether bootstrap samples are used when building trees. values: [False,True]	False
select	class_weight	Weights associated with classes in the form {class_label: weight}. If not given, all classes are supposed to have weight one. For multi-output problems, a list of dicts can be provided in the same order as the columns of y. The "auto" mode uses the values of y to automatically adjust weights inversely proportional to class frequencies in the input data. The "subsample" mode is the same as "auto" except that weights are computed based on the bootstrap sample for every tree grown. For multi-output, the weights of each column of y will be multiplied. Note that these weights will be multiplied with sample_weight (passed through the fit method) if sample_weight is specified. values: [None,'balanced_subsample']	None
select	criterion	The function to measure the quality of a split. Supported criteria are "gini" for the Gini impurity and "entropy" for the information gain. Note: this parameter is tree-specific. values: ['gini','entropy']	gini
select	estimator	The estimator to use values: ['RandomForestClassifier','ExtraTreesClassifier']	ExtraTreesClassifier
select	max_depth	The maximum depth of the tree. If None, then nodes are expanded until all leaves are pure or until all leaves contain less than min_samples_split samples. Ignored if max_leaf_nodes is not None. Note: this parameter is tree-specific. values: {'intgrid':[1,100],'select':['None']}	None
multi	max_features	The number of features to consider when looking for the best split: - If int, then consider max_features features at each split. - If float, then max_features is a percentage and int(max_features * n_features) features are considered at each split. - If "auto", then max_features=sqrt(n_features). - If "sqrt", then max_features=sqrt(n_features). - If "log2", then max_features=log2(n_features). - If None, then max_features=n_features. Note: the search for a split does not stop until at least one valid partition of the node samples is found, even if it requires to effectively inspect more than max_features features. Note: this parameter is tree-specific. values:	0.3

		{'select':['auto','sqrt','log2','n/3'], 'intgrid':[1,int(1e8)], 'floatgrid':[0.,1.]}	
select	max_leaf_nodes	Grow trees with max_leaf_nodes in best-first fashion. Best nodes are defined as relative reduction in impurity. If None then unlimited number of leaf nodes. values: {'intgrid':[2,100000000],'select':['None']}	50
intgrid	min_samples_leaf	The minimum number of samples in newly created leaves. A split is discarded if after the split, one of the leaves would contain less than min_samples_leaf samples. Note: this parameter is tree-specific. values: [1,1000]	5
intgrid	min_samples_split	The minimum number of samples required to split an internal node. Note: this parameter is tree-specific. values: [1,1000]	10
int	n_estimators	The number of trees in the forest. values:[1,2000]	500
int	random_state	Is the seed used by the random number generator. values: [0,int(1e9)]	1234
bool	replace	If the sampling should be done with or without replacement. values: [False,True]	True
float	subsample	The fraction of samples to be used for building trees. values: [0.0,1.0]	1.0

Eureqa Generalized Additive Model Classifier (1000 Generations)

Median Value-Based Numeric Imputation (V2 with quick median algorithm)

Type	Name	Description	Best Searched
bool	scale_small	True if small values (range of the numeric variable is <= 1) are to be scaled. values: [False, True]	False
int	threshold	Minimum number of required finite elements in a column to impute the data onto NaNs and INFs. values: [1, 99999]	10

One-Hot Encoding Task

Type	Name	Description	Best Searched
int	card_max	An integer that specifies the maximum number of unique values. values: [1, 99999]	10000
int	card_min	An integer that specifies the minimum number of unique values. values: [1, 99999]	11
bool	drop_cols	drop_cols, If True, drop last level of each feature values: [False, True]	False
select	flag	flag, If all, add highcat-cols to metadata values: ['None', 'all']	all
int	max_features	If the total number of categories created across all features exceeds this value, the top max_features most frequent categories will persist. All others will be either thrown out or grouped. A value of None disables the limit. values: [1, 999999]	None
int	min_support	The minimum number of records for a category to be represented in one hot encoding. If a category has fewer counts	5

		it will be grouped with other small cardinality values. values: [1, 99999]	

Elasticnet Classifier model based on block coordinate descent

Type	Name	Description	Best Searched
select	beta_transform	beta_transform is a parameter used for blenders. If beta_transform is set to 'blender', coefficients are non-negative and are all in [0, 1]. Very large weight of the penalty term will get you the average blender. values: ['id', 'blender']	id
multi	enet_alpha	The ElasticNet mixing parameter, with 0 <= alpha <= 1. For alpha = 0 the penalty is an L2 penalty. For alpha = 1 it is an L1 penalty. For 0 < alpha < 1, the penalty is a combination of L1 and L2. 'auto' grid of 11 numbers spaced evenly from 0.0 to 1.0 values: {'floatgrid': [0, 1], 'select': ['auto']}	0.0
multi	enet_lambda	The weight for the penalty term 'auto' searches a grid of 50 numbers spaced evenly on a log10 scale from 3.16e-07 to 3.16e-01 values: {'floatgrid':[1e-10,1e10],'select':['auto']}	auto
bool	fit_alpha_scaler	If it is set to True, the weight of the penalty term is scaled. A weight of 1 would lead to an intercept only model and a weight of 0 won't apply any penalty. With this parameter set to True, a penalty > 1 would not make sense any more. values: [False, True]	True
bool	fit_intercept	whether to calculate the intercept for this model. If set to false, no intercept will be used in calculations (e.g. data is expected to be already centered). values: [False, True]	True
select	loss	The loss function to be used. values: ['log']	log
int	max_iter	The maximum number of iterations values: [1, 1e6]	100
int	random_state	The seed of the pseudo random number generator to use. values: [0, int(1e9)]	1234
float	sigma	Constant used in the line search sufficient decrease condition. values': [0, 1e-6]	1e-06
float	tol	The tolerance for the optimization: if the updates are smaller than tol, the optimization code checks the dual gap for optimality and continues until it is smaller than tol. values: [1e-10, 1e10]	0.0001
bool	warm_start	When set to True, reuse the solution of the previous call to fit as initialization, otherwise, just erase the previous solution. values: [False, True]	False

Prediction Model Parameters Eureqa Generalized Additive Model Classifier

Type	Name	Description	Best Searched
int or select	EUREQA_building_block__absolute_value	'Absolute Value' building block. Allows Eureqa to use the "abs()" operator in model expressions, and sets its complexity penalty. Usage: abs(x), which returns the positive	None

		value of x, without regard for its sign. values: [0, 100] or "Disabled".	
int or select	EUREQA_building_block__addition	'Addition' building block. Allows Eureqa to use the "+" operator in model expressions, and sets its complexity penalty. Usage: x + y or add(x, y), which returns the sum of x and y. values: [0, 100] or "Disabled".	None
int or select	EUREQA_building_block__arccosine	'Arccosine' building block. Allows Eureqa to use the "acos()" operator in model expressions, and sets its complexity penalty. Usage: acos(x). (The standard trigonometric arccosine function.) values: [0, 100] or "Disabled".	None
int or select	EUREQA_building_block__arcsine	'Arcsine' building block. Allows Eureqa to use the "asin()" operator in model expressions, and sets its complexity penalty. Usage: asin(x). (The standard trigonometric arcsine function.) values: [0, 100] or "Disabled".	None
int or select	EUREQA_building_block__arctangent	'Arctangent' building block. Allows Eureqa to use the "atan()" operator in model expressions, and sets its complexity penalty. Usage: atan(x). (The standard trigonometric arctangent function.) values: [0, 100] or "Disabled".	None
int or select	EUREQA_building_block__ceiling	'Ceiling' building block. Allows Eureqa to use the "ceil()" operator in model expressions, and sets its complexity penalty. Usage: ceil(x), which returns the smallest integer not less than x. values: [0, 100] or "Disabled".	None
int or select	EUREQA_building_block__complementary_error_function	'Complementary Error Function' building block. Allows Eureqa to use the "erfc()" operator in model expressions, and sets its complexity penalty. Usage: erfc(x). 1.0 - erf(x) where erf(x) is the integral of the normal distribution and returns a value between 2 and 0. values: [0, 100] or "Disabled".	None
int or select	EUREQA_building_block__constant	'Constant' building block. Allows Eureqa to use constants in model expressions, and sets their complexity penalty. Usage: c, where c is a real valued constant. values: [0, 100] or "Disabled"	None
int or select	EUREQA_building_block__cosine	'Cosine' building block. Allows Eureqa to use the "cos()" operator in model expressions, and sets its complexity penalty. Usage: cos(x), where the angle (x) is in radians. (The standard trigonometric cosine function.) values: [0, 100] or "Disabled".	None
int or select	EUREQA_building_block__division	'Division' building block. Allows Eureqa to use the "/" operator in model expressions, and sets its complexity penalty. Usage: x / y or div(x, y), which returns the quotient of x and y (where y must be non-zero). values: [0, 100] or "Disabled".	None
int or select	EUREQA_building_block__equal-to	'Equal-To' building block. Allows Eureqa to use the "=" operator in model expressions, and sets its complexity penalty. Usage: equal(x, y) or x = y, which returns 1 if x is numerically equal to y, 0 otherwise. values: [0, 100] or "Disabled".	None

int or select	EUREQA_building_block__error_function	'Error Function' building block. Allows Eureqa to use the "erf()" operator in model expressions, and sets its complexity penalty. Usage: erf(x). Integral of the normal distribution; returns a value between -1 and +1. values: [0, 100] or "Disabled".	None
int or select	EUREQA_building_block__exponential	'Exponential' building block. Allows Eureqa to use the "exp()" operator in model expressions, and sets its complexity penalty. Usage: exp(x), which returns e^x. values: [0, 100] or "Disabled".	None
int or select	EUREQA_building_block__factorial	'Factorial' building block. Allows Eureqa to use the "!" operator in model expressions, and sets its complexity penalty. Usage: factorial(x) or x!, which returns the product of all positive integers from 1 to x. values: [0, 100] or "Disabled".	None
int or select	EUREQA_building_block__floor	'Floor' building block. Allows Eureqa to use the "floor()" operator in model expressions, and sets its complexity penalty. Usage: floor(x), which returns the largest integer not greater than x. values: [0, 100] or "Disabled".	None
int or select	EUREQA_building_block__gaussian_function	'Gaussian Function' building block. Allows Eureqa to use the "gauss()" operator in model expressions, and sets its complexity penalty. Usage: gauss(x), which returns exp(-x^2). This is a bell-shaped squashing function. values: [0, 100] or "Disabled".	None
int or select	EUREQA_building_block__greater-than	'Greater-Than' building block. Allows Eureqa to use the ">" operator in model expressions, and sets its complexity penalty. Usage: greater(x, y) or x > y, which returns 1 if x > y, 0 otherwise. values: [0, 100] or "Disabled".	None
int or select	EUREQA_building_block__greater-than-or-equal	'Greater-Than-Or-Equal' building block. Allows Eureqa to use the ">=" operator in model expressions, and sets its complexity penalty. Usage: greater_or_equal(x, y) or x >= y, which returns 1 if x >= y, 0 otherwise. values: [0, 100] or "Disabled".	None
int or select	EUREQA_building_block__hyperbolic_cosine	'Hyperbolic Cosine' building block. Allows Eureqa to use the "cosh()" operator in model expressions, and sets its complexity penalty. Usage: cosh(x). (The standard trigonometric hyperbolic cosine function.) values: [0, 100] or "Disabled".	None
int or select	EUREQA_building_block__hyperbolic_sine	'Hyperbolic Sine' building block. Allows Eureqa to use the "sinh()" operator in model expressions, and sets its complexity penalty. Usage: sinh(x). (The standard trigonometric hyperbolic sine function.) values: [0, 100] or "Disabled".	None
int or select	EUREQA_building_block__hyperbolic_tangent	'Hyperbolic Tangent' building block. Allows Eureqa to use the "tanh()" operator in model expressions, and sets its complexity penalty. Usage: tanh(x). (The hyperbolic tangent of x.) Hyperbolic tangent is a common squashing function that returns a value between -1 and +1. values: [0, 100] or "Disabled".	None
int or select	EUREQA_building_block__if-then-else	'If-Then-Else' building block. Allows Eureqa to use the "if()" operator in model expressions, and sets its	None

		complexity penalty. Usage: if(x, y, z), which returns y if x is greater than 0, z otherwise; if x is nan, the function returns z. values: [0, 100] or "Disabled".	
int or select	EUREQA_building_block__input_variable	'Input Variable' building block. Allows Eureqa to use variables in model expressions, and sets their complexity penalty. Usage: x, where x is a variable in your prepared dataset. values: [0, 100] or "Disabled"	None
int or select	EUREQA_building_block__integer_constant	'Integer Constant' building block. Allows Eureqa to use integer constants in model expressions, and sets their complexity penalty. Usage: c, where c is an integer constant. values: [0, 100] or "Disabled"	None
int or select	EUREQA_building_block__inverse_hyperbolic_cosine	'Inverse Hyperbolic Cosine' building block. Allows Eureqa to use the "acosh()" operator in model expressions, and sets its complexity penalty. Usage: acosh(x). (The standard inverse hyperbolic cosine function.) values: [0, 100] or "Disabled".	None
int or select	EUREQA_building_block__inverse_hyperbolic_sine	'Inverse Hyperbolic Sine' building block. Allows Eureqa to use the "asinh()" operator in model expressions, and sets its complexity penalty. Usage: asinh(x). (The standard inverse hyperbolic sine function.) values: [0, 100] or "Disabled".	None
int or select	EUREQA_building_block__inverse_hyperbolic_tangent	'Inverse Hyperbolic Tangent' building block. Allows Eureqa to use the "atanh()" operator in model expressions, and sets its complexity penalty. Usage: atanh(x). (The standard inverse hyperbolic tangent function.) values: [0, 100] or "Disabled".	None
int or select	EUREQA_building_block__less-than	'Less-Than' building block. Allows Eureqa to use the "<" operator in model expressions, and sets its complexity penalty. Usage: less(x, y) or x < y, which returns 1 if x < y, 0 otherwise. values: [0, 100] or "Disabled".	None
int or select	EUREQA_building_block__less-than-or-equal	'Less-Than-Or-Equal' building block. Allows Eureqa to use the "<=" operator in model expressions, and sets its complexity penalty. Usage: less_or_equal(x, y) or x <= y, which returns 1 if x <= y, 0 otherwise. values: [0, 100] or "Disabled".	None
int or select	EUREQA_building_block__logical_and	'Logical And' building block. Allows Eureqa to use the "and" operator in model expressions, and sets its complexity penalty. Usage: and(x, y), which returns 1 if both x and y are greater than 0, 0 otherwise. values: [0, 100] or "Disabled".	None
int or select	EUREQA_building_block__logical_not	'Logical Not' building block. Allows Eureqa to use the "not" operator in model expressions, and sets its complexity penalty. Usage: not(x), which returns 0 if x is greater than 0, 1 otherwise. values: [0, 100] or "Disabled".	None
int or select	EUREQA_building_block__logical_or	'Logical Or' building block. Allows Eureqa to use the "or" operator in model expressions, and sets its complexity penalty. Usage: or(x, y), which returns 1 if either x or y are	None

		greater than 0, 0 otherwise. values: [0, 100] or "Disabled".	
int or select	EUREQA_building_block__logical_xor	‘Logical Xor’ building block. Allows Eureqa to use the “xor” operator in model expressions, and sets its complexity penalty. Usage: xor(x, y), which returns 1 if (x <= 0 and y > 0) or (x > 0 and y <= 0), 0 otherwise. values: [0, 100] or "Disabled".	None
int or select	EUREQA_building_block__logistic_function	‘Logistic Function’ building block. Allows Eureqa to use the “logistic()” operator in model expressions, and sets its complexity penalty. Usage: logistic(x), which returns 1/(1 + exp(-x)). This is a common sigmoid (s-shaped) squashing function that returns a value between 0 and 1. values: [0, 100] or "Disabled".	None
int or select	EUREQA_building_block__maximum	‘Maximum’ building block. Allows Eureqa to use the “max()” operator in model expressions, and sets its complexity penalty. Usage: max(x, y), which returns the maximum (signed) result of x and y. values: [0, 100] or "Disabled".	None
int or select	EUREQA_building_block__minimum	‘Minimum’ building block. Allows Eureqa to use the “min()” operator in model expressions, and sets its complexity penalty. Usage: min(x, y), which returns the minimum (signed) result of x and y. values: [0, 100] or "Disabled".	None
int or select	EUREQA_building_block__modulo	‘Modulo’ building block. Allows Eureqa to use the “mod()” operator in model expressions, and sets its complexity penalty. Usage: mod(x, y), which returns the remainder of x / y. values: [0, 100] or "Disabled".	None
int or select	EUREQA_building_block__multiplication	‘Multiplication’ building block. Allows Eureqa to use the “*” operator in model expressions, and sets its complexity penalty. Usage: x * y or mul(x, y), which returns the product of x and y. values: [0, 100] or "Disabled".	None
int or select	EUREQA_building_block__natural_logarithm	‘Natural Logarithm’ building block. Allows Eureqa to use the “log()” operator in model expressions, and sets its complexity penalty. Usage: log(x), which returns the natural logarithm (base e) of x. values: [0, 100] or "Disabled".	None
int or select	EUREQA_building_block__negation	‘Negation’ building block. Allows Eureqa to use the “-” unary operator in model expressions, and sets its complexity penalty. Usage: -x, which returns the inverse of x. values: [0, 100] or "Disabled".	None
int or select	EUREQA_building_block__power	‘Power’ building block. Allows Eureqa to use the “^” operator in model expressions, and sets its complexity penalty. values: [0, 100] or "Disabled".	None
int or select	EUREQA_building_block__round	‘Round’ building block. Allows Eureqa to use the “round()” operator in model expressions, and sets its complexity penalty. Usage: round(x), which returns an integer of x rounded to the nearest integer. values: [0, 100] or "Disabled".	None
int or select	EUREQA_building_block__sign_function	‘Sign Function’ building block. Allows Eureqa to use the “sign()” operator in model expressions, and sets its complexity penalty. Usage: sgn(x),	None

		which returns -1 if x is negative, +1 if x is positive, and 0 if x is zero. values: [0, 100] or "Disabled".	
int or select	EUREQA_building_block__sine	'Sine' building block. Allows Eureqa to use the "sin()" operator in model expressions, and sets its complexity penalty. Usage: sin(x), where the angle (x) is in radians. (The standard trigonometric sine function.) values: [0, 100] or "Disabled".	None
int or select	EUREQA_building_block__square_root	'Square Root' building block. Allows Eureqa to use the "sqrt()" operator in model expressions, and sets its complexity penalty. Usage: sqrt(x), which returns the square root of x (where x must be positive). values: [0, 100] or "Disabled".	None
int or select	EUREQA_building_block__step_function	'Step Function' building block. Allows Eureqa to use the "step()" operator in model expressions, and sets its complexity penalty. Usage: step(x), which returns 1 if x is positive, 0 otherwise. values: [0, 100] or "Disabled".	None
int or select	EUREQA_building_block__subtraction	'Subtraction' building block. Allows Eureqa to use the "-" binary operator in model expressions, and sets its complexity penalty. Usage: x - y or sub(x, y), which returns the difference of x and y. values: [0, 100] or "Disabled".	None
int or select	EUREQA_building_block__tangent	'Tangent' building block. Allows Eureqa to use the "tan()" operator in model expressions, and sets its complexity penalty. Usage: tan(x), where the angle (x) is in radians. (The standard trigonometric tangent function.) values: [0, 100] or "Disabled".	None
int or select	EUREQA_building_block__two-argument_arctangent	'Two-Argument Arctangent' building block. Allows Eureqa to use the "atan2()" operator in model expressions, and sets its complexity penalty. Usage: atan2(y, x). (The standard trigonometric two-argument arctangent function.) values: [0, 100] or "Disabled".	None
int	EUREQA_max_generations	The maximum number of evolutionary generations to run. Eureqa will run until either of max_generations or timeout_sec is reached. values: [0, 1e16]	None
int	EUREQA_num_threads	The number of threads Eureqa will run with. Ideally equal to the number of cores available	None
string	EUREQA_prior_solutions	Prior Eureqa Solutions. This field contains multiple Eureqa Expressions, one per line. Each Expression should be a valid Eureqa Solution, such as a Solution returned by a previous run of Eureqa. (You may need to edit the Solution such that the target variable is entered as "Target", not the original column name of the target.) Each expression is fed into Eureqa's initial evolutionary population. Eureqa makes no guarantees about keeping the form or content of these expressions in the final Pareto front of expressions that it generates, but if the expressions are good models or if they contain sub-expressions that are predictive features, Eureqa will generally take advantage of that information to converge on good	None

		solutions more quickly. values: multiple lines, each line is a valid Eureqa Expression as a string	
int	EUREQA_random_seed	Constant to seed Eureqa's pseudo-random number generator. Different values will cause Eureqa to generate different models on the same data and other input parameters. values: [0, 1e16]	None
select	EUREQA_split_mode	Whether to perform in-order (2) or random (1) splitting within the training set, for evolutionary re-training and re-validaton. values: [1, 2] values: [0, 1e5]	None
select	EUREQA_sync_migrations	Should Eureqa's migrations be synchronized? If they are synchronized, Eureqa's fit() function will be deterministic (repeated runs on the same data and parameters should produce the same models). Note that synchronization slows modeling down and models will take more time to generate. values: [False, True]	None
string	EUREQA_target_expression_string	Eureqa Target Expression. Constrains the form of the models that Eureqa will consider. This field is typically of the form "Target = <some Eureqa Expression>". See the description of "Eureqa Expressions". The Expression must contain an equality operator, ie., it must be a full equation with a left-hand and a right-hand side. Note that Target Expressions will usually use function operators in their expressions, as well as regular expression operators. Function operators are templates / pattern matchers. For example, "Target = f(x, y z)" will match any expression that uses the variables x, y, and/or z (but will fail to match any function that uses other variables); "Target = f1(x, y) + f2(z)" will match "x*y + z" but it will not match "x*z + y"; "Target = f(sin(x))" will match "2*sin(x)" but it will not match "sin(2*x)". This is a hard constraint. If you want to seed Eureqa with information from existing models, see prior_solutions. If this field is left blank, Eureqa will automatically generate an appropriate target expression as part of the model fitting process. This expression will be logged to the model log (visible in the UI). values: valid Eureqa Expression, as a string. Must contain an equality operator.	None
float	EUREQA_timeout_sec	The duration of time to run the Eureqa search algorithm for Eureqa will run until either of max_generations or timeout_sec is reached. values: [0, 1e16]	None
float	EUREQA_training_fraction	What fraction of the DataRobot training data to use for Eureqa evolutionary training? This field is ignored if training_split_expression is set. Note that training_fraction + validation_fraction does not have to equal 1.0. If it is less than 1.0, some rows in the data are ignored. If it is greater than 1.0, Eureqa's training and validation sets overlap. (This is not recommended, but may be required in some modeling scenarios involving small datasets. Note that DataRobot will still typically have a separate validation and holdout set.) values: [0.0, 1.0]	None

string	EUREQA_training_split_expr	Eureqa Training Split Expression. Can be any valid Eureqa Expression, including a simple variable name. The expression should evaluate to either 0.0 or 1.0. If it evaluates to 1.0 on a specific row, that row is used for training data. If this field is not set, training_fraction is used instead. Note that setting training_split_expr does not affect validation rows. If you want all non-training rows to be in the validation set, make sure to set validation_split_expr accordingly. values: valid Eureqa Expression, as a string	None
float	EUREQA_validation_fraction	What fraction of the DataRobot training data to use for Eureqa evolutionary validation? This field is ignored if validation_split_expression is set. Note that training_fraction + validation_fraction does not have to equal 1.0. If it is less than 1.0, some rows in the data are ignored. If it is greater than 1.0, Eureqa's training and validation sets overlap. (This is not recommended, but may be required in some modeling scenarios involving small datasets. Note that DataRobot will still typically have a separate validation and holdout set.) values: [0.0, 1.0]	None
string	EUREQA_validation_split_expr	Eureqa Validation Split Expression. Can be any valid Eureqa Expression, including a simple variable name. The expression should evaluate to either 0.0 or 1.0. If it evaluates to 1.0 on a specific row, that row is used for validation data. If this field is not set, validation_fraction is used instead. Note that setting validation_split_expr does not impact training rows. If you want all non-validation rows to be in the training set, make sure to set training_split_expr accordingly. values: valid Eureqa Expression, as a string	None
string	EUREQA_weight_expr	Eureqa Weight Expression. Weights each row when evaluating the error of that row. The exact meaning of the weight depends on the specific value of error_metric. Typically the per-row error is multiplied by the weight before being combined with other per-row weights into the aggregate weight. If this field is left as the empty string, Eureqa falls back to DataRobot's default behavior. values: valid Eureqa Expression, as a string	None
select	XGB_base_margin_initialize	If True, the intercept is initialized to the log odds of the target. values: [False, True]	True
int	XGB_class_count	For multiclass only. The number of target classes. values: [0, MAX_TARGET_CLASS_COUNT]	None
floatgrid	XGB_colsample_bylevel	Subsample the features before each split in a tree. values: [0.1,1]	1.0
floatgrid	XGB_colsample_bytree	Subsample ratio of columns when constructing each tree. By default, the value of colsample_bytree for XGBoost classes is 1.0. However, based on the training data, DataRobot may choose a different initial value for this parameter. values: [0,1]	0.3
int	XGB_interval	Sets the interval for early stopping values: [2, 500]	None

floatgrid	XGB_learning_rate	Shrinks the contribution of each tree by learning_rate. There is a trade-off between learning_rate (lr) and n_estimators(n). values: [5e-4,1]	0.05
select	XGB_loss	loss, loss function to be optimized. 'deviance' refers to deviance (= logistic regression) for classification with probabilistic outputs. values: ['deviance', 'softprob']	None
int	XGB_max_bin	This is only used if 'hist' is specified as tree_method. Maximum number of discrete bins to bucket continuous features. Increasing this number improves the optimality of splits at the cost of higher computation time. values: [16, 2048]	256
floatgrid	XGB_max_delta_step	Maximum delta step we allow each tree's weight estimation to be. If the value is set to 0, it means there is no constraint. If it is set to a positive value, it can help making the update step more conservative. Usually this parameter is not needed, but it might help in logistic regression when class is extremely imbalanced. Set it to value of 1-10 might help control the update values: [0,100]	0.0
intgrid	XGB_max_depth	maximum depth of the individual regression estimators. The maximum depth limits the number of nodes in the tree. Tune this parameter for best performance; the best value depends on the interaction of the input variables. Deeper the tree the more variable interactions the model can capture. For frozen models on larger sample sizes than parent model we increase the value of max_depth to retain similar accuracy. values: [1, 16]	3
floatgrid	XGB_min_child_weight	Minimum sum of instance weight(hessian) needed in a child. If the tree partition step results in a leaf node with the sum of instance weight less than min_child_weight, then the building process will give up further partitioning. In linear regression mode, this simply corresponds to minimum number of instances needed to be in each node. The larger, the more conservative the algorithm will be. values: [0.01,float(1e5)]	5.0
floatgrid	XGB_min_split_loss	Minimum loss reduction required to make a further partition on a leaf node of the tree. the larger, the more conservative the algorithm will be. values: [0,1e5]	0.01
float	XGB_missing_value	The float value that should be treated as a missing value. When mono_up or mono_down are set, missing value will be set to -9999.0. values: [float(-1e5),float(1e5)]	None
string	XGB_mono_down	The id of the featurelist that defines the set of features with a monotonically decreasing relationship to the target.	None
string	XGB_mono_up	The id of the featurelist that defines the set of features with a monotonically increasing relationship to the target.	None
int	XGB_n_estimators	The number of boosting stages to perform. Gradient boosting is fairly robust to over-fitting so a large	190

		number usually results in better performance. values: [1,20000]	
intgrid	XGB_num_parallel_tree	Number of parallel trees created in each boosting stage. When this value is greater than 1, the model becomes a gradient-boosted random forest with (num_parallel_tree * n_estimators) trees. values: [1,16]	1
intgrid	XGB_random_state	The seed used in the random number generator 'values': [0, int(1e9)]	1234
multi	XGB_reg_alpha	L1 regularization term on weights, increase this value will make model more conservative. values: {'floatgrid': [0, 1e6], 'select': ['auto']}	0.0
multi	XGB_reg_lambda	L2 regularization term on weights, increase this value will make model more conservative. values: {'floatgrid': [0, 1e6], 'select': ['auto']}	1.0
float	XGB_scale_pos_weight	Scaling factor for examples in the positive class. values: [0,float(1e9)]	1.0
int	XGB_smooth_interval	Sets the minimum interval for early stopping values: [2, 1000]	None
floatgrid	XGB_subsample	subsample ratio of the training instance. Setting it to 0.5 means that XGBoost randomly collected half of the data instances to grow trees and this will prevent overfitting.	1.0
select	XGB_tree_method	The tree construction algorithm to be used. 'auto': Use heuristic to choose faster one. For small to medium dataset(<4M rows), exact greedy will be used. For very large-dataset(>=4M rows), approximate algorithm will be chosen. 'exact':Exact greedy algorithm. 'approx':Approximate greedy algorithm using sketching and histogram. 'hist': Fast histogram optimized approximate greedy algorithm. It uses some performance improvements such as bins caching. values: ['auto', 'exact', 'approx', 'hist']	auto
int	feature_interaction_max_features	Specifies the max number of one vs all interactions to include in the pairwise calculations. For example, the default value of 50 will yield (50 ^ 2 - 50) / 2 pairwise interactions if feature_interaction_threshold is zero values: [0, int(1e5)]	50
int	feature_interaction_sampling	Specifies the extent (in number of rows) of downsampling used in the interaction calculations values': [1000, int(1e6)]	2500
float	feature_interaction_threshold	Specifies the minimum value a one vs all interaction strength must have to be include a given feature in the pairwise calculations. Zero includes all and one excludes all. values: [0.0, 1.0]	0.1
int	feature_selection_max_features	The maximum number of features to include. ``values: {'int': [1, int(1e10)], 'select': ['no_limit']}`	no_limit
select	feature_selection_method	Method used to select features values: ['per_variable', 'cumulative', 'no_selection']	no_selection
int	feature_selection_min_features	The minimum number of features to include. values: [1, int(1e5)]	1
float	feature_selection_threshold	The threshold used to select features. For cumulative method, threshold is	0.001

		typically close to 1 in order to retain more signal (ex:0.98). For per_variable method, threshold would be close to 0 in order to discard only features with low signal. values: [0.0001, 1]	
select	highdim_modeling	Whether to include high cardinality and text features. values: [False, True]	True
int	subsample	Number of rows to sample for fitting the Eureqa model. values: [1000, 1e7]	10000

Target 2: AVG Blender (RandomForest Classifier (Gini), eXtreme Gradient Boosted Trees Classifier (learning rate=0.01), Eureqa Classifier (Default Search 3000 Generations))

RandomForest Classifier (Gini)

Ordinal scale converter of categorical features

Type	Name	Description	Best Searched
select	add_cols_metadata	If specified, add -cols to metadata. values: [False, True]	False
select	add_maps_metadata	If specified, add -maps to metadata. values: [False, True]	False
multi	card_max	Maximum number of categorical feature levels allowed. If None, a feature with any number of levels is allowed. values: {'int': [1, 9999999], 'select': None}	None
select	method	Method used in the encoding. None: uses random_scale. random: random ordering of levels, lex: lexicographical ordering by category level names, freq: frequency ordering from least frequent to most frequent, resp: response ordering. values: ['None', 'random', 'lex', 'freq', 'resp']	random
int	min_support	Minimum number of levels required for a category to be represented on the ordinal scale. If a category level count is below the minimum, it will be grouped with other small cardinality levels or encoded as a missing value, depending on the value of other_category. values: [1, 99999]	5
int	offset	Shift the ordinal scale of ordinal encoder values: [0, 99999]	0
bool	other_category	If True, small cardinality values are mapped to a dedicated value (-1), otherwise they are encoded as missing values (-2). values: [False, True]	True
bool	random_scale	Applies if method is None. If random_scale is True, random ordering is used for the ordinal scale. If it is False, lexicographical ordering is used. values: [False, True]	True
int	seed	The RNG seed. values: [0, 99999]	1234

Median Value-Based Numeric Imputation (V2 with quick median algorithm)

Type	Name	Description	Best Searched
bool	scale_small	True if small values (range of the numeric variable is <= 1) are to be scaled. values: [False, True]	False
int	threshold	Minimum number of required finite elements in a column to impute the data onto NaNs and INFs. values: [1, 99999]	10

Prediction Model Parameters Random Forest Classifier

Type	Name	Description	Best Searched
bool	balanced_bootstrap	Whether the sampling probability per class should be inverse to the class frequency values: [False,True]	False
bool	bootstrap	Whether bootstrap samples are used when building trees. values: [False,True]	False
select	class_weight	Weights associated with classes in the form {class_label: weight}. If not given, all classes are supposed to have weight one. For multi-output problems, a list of dicts can be provided in the same order as the columns of y. The "auto" mode uses the values of y to automatically adjust weights inversely proportional to class frequencies in the input data. The "subsample" mode is the same as "auto" except that weights are computed based on the bootstrap sample for every tree grown. For multi-output, the weights of each column of y will be multiplied. Note that these weights will be multiplied with sample_weight (passed through the fit method) if sample_weight is specified. values: [None,'balanced_subsample']	None
select	criterion	The function to measure the quality of a split. Supported criteria are "gini" for the Gini impurity and "entropy" for the information gain. Note: this parameter is tree-specific. values: ['gini','entropy']	gini
select	estimator	The estimator to use values: ['RandomForestClassifier','ExtraTreesClassifier']	RandomForestClassifier
select	max_depth	The maximum depth of the tree. If None, then nodes are expanded until all leaves are pure or until all leaves contain less than min_samples_split samples. Ignored if max_leaf_nodes is not None. Note: this parameter is tree-specific. values: {'intgrid':[1,100],'select':['None']}	None
multi	max_features	The number of features to consider when looking for the best split: - If int, then consider max_features features at each split. - If float, then max_features is a percentage and int(max_features * n_features) features are considered at each split. - If "auto", then max_features=sqrt(n_features). - If "sqrt", then max_features=sqrt(n_features). - If "log2", then max_features=log2(n_features). - If None, then max_features=n_features. Note: the search for a split does not stop until at least one valid partition of the node samples is found, even if it requires to effectively inspect more than max_features features. Note: this parameter is tree-specific. values: {'select':['auto','sqrt','log2','n/3'], 'intgrid':[1,int(1e8)], 'floatgrid':[0.,1.]}	0.4
select	max_leaf_nodes	Grow trees with max_leaf_nodes in best-first fashion. Best nodes are defined as relative reduction in impurity. If None then unlimited number of leaf nodes. values: {'intgrid':[2,100000000],'select':['None']}	50

intgrid	min_samples_leaf	The minimum number of samples in newly created leaves. A split is discarded if after the split, one of the leaves would contain less than min_samples_leaf samples. Note: this parameter is tree-specific. values: [1,1000]	10
intgrid	min_samples_split	The minimum number of samples required to split an internal node. Note: this parameter is tree-specific. values: [1,1000]	2
int	n_estimators	The number of trees in the forest. values:[1,2000]	500
int	random_state	Is the seed used by the random number generator. values: [0,int(1e9)]	1234
bool	replace	If the sampling should be done with or without replacement. values: [False,True]	True
float	subsample	The fraction of samples to be used for building trees. values: [0.0,1.0]	1.0

eXtreme Gradient Boosting Classifier (learning rate=0.01)

One-Hot Encoding

Type	Name	Description	Best Searched
int	card_max	An integer that specifies the maximum number of unique values. values: [1, 99999]	10000
int	card_min	An integer that specifies the minimum number of unique values. values: [1, 99999]	11
bool	drop_cols	drop_cols, If True, drop last level of each feature values: [False, True]	False
select	flag	flag, If all, add highcat-cols to metadata values: ['None', 'all']	all
int	max_features	If the total number of categories created across all features exceeds this value, the top max_features most frequent categories will persist. All others will be either thrown out or grouped. A value of None disables the limit. values: [1, 999999]	None
int	min_support	The minimum number of records for a category to be represented in one hot encoding. If a category has fewer counts it will be grouped with other small cardinality values. values: [1, 99999]	0

Median Value-Based Numeric Imputation (V2 with quick median algorithm)

Type	Name	Description	Best Searched
bool	scale_small	True if small values (range of the numeric variable is <= 1) are to be scaled. values: [False, True]	True
int	threshold	Minimum number of required finite elements in a column to impute the data onto NaNs and INFs. values: [1, 99999]	10

Feature selection for Ratios/Differences

Type	Name	Description	Best Searched

int	FeatureSelection: max_features	The maximum number of features to include. ``values: {'int': [1, int(1e10)], 'select': ['no_limit']}`	50
select	FeatureSelection: method	Method used to select features values: ['per_variable', 'cumulative', 'no_selection']	per_variable
int	FeatureSelection: min_features	The minimum number of features to include. values: [1, int(1e5)]	1
float	FeatureSelection: threshold	The threshold used to select features. For cumulative method, threshold is typically close to 1 in order to retain more signal (ex:0.98). For per_variable method, threshold would be close to 0 in order to discard only features with low signal. values: [0.0001, 1]	0.001

Geedy Search for differences

Type	Name	Description	Best Searched
float	maximum scale ratio	Defines the highest scale ratio between 2 raw features mean to test the difference as a new feature values: [1e-06,10]	3.0
int	min samples split	Defines the minimum number of samples required to split an internal node in the tree used to test difference features. values: [1, 10000]	10
float	minimum correlation	Defines the minimum correlation between 2 raw features to test the difference as a new feature values: [0, 0.99]	0.3
float	minimum improvement	Defines the minimum improvment vs raw features only values: [0, 0.99]	0.01
int	seed	Defines the random seed for the cross validation partition values: [0, int(1e20)]	1234
float	test size	test_size of holdout set to measure accuracy performance values: [0.01, 0.99]	0.2

Greedy Search for ratios

Type	Name	Description	Best Searched
select	distribution	The distribution to use values: ['Gaussian', 'Poisson', 'Bernoulli', 'Gamma', 'Tweedie']	Bernoulli
float	minimum correlation	Defines the minimum correlation between 2 raw features to test the ratio as a new feature values: [0, 0.99]	0.3
float	minimum improvement	Defines the minimum improvment vs raws features only values: [0, 0.99]	0.01
int	seed	Defines the random seed for the cross validation partition values: [0, int(1e20)]	1234
select	test size	test_size of holdout set to measure accuracy performance values: [0.01, 0.99]	0.2

Prediction Model Parameters Extreme Gradient Boosting Classifier with Grid Search support

Type	Name	Description	Best Searched
select	base_margin_initialize	If True, the intercept is initialized to the log odds of the target. values: [False, True]	False
int	class_count	Number of target classes (multiclass only). values: [0, MAX_TARGET_CLASS_COUNT]	None
floatgrid	colsample_bylevel	Subsample of the features before each split in a tree. values: [0.1,1]	1.0
floatgrid	colsample_bytree	Subsample ratio of columns when constructing each tree. By default, the value of colsample_bytree for XGBoost classes is 1.0. However, based on the training data, DataRobot may choose a different initial value for this parameter. values: [0,1]	0.2
floatgrid	learning_rate	Shrinks the contribution of each tree by learning_rate. There is a trade-off between learning_rate (lr) and n_estimators(n). values: [5e-4,1]	0.01
select	loss	Loss function to be used during optimization. 'deviance' refers to deviance (= logistic regression) for classification with probabilistic outputs. values: ['deviance', 'softprob']	deviance
int	max_bin	Used when tree_method is set to 'hist'. Maximum number of discrete bins to bucket continuous features. Increasing this number improves the optimality of splits at the cost of higher computation time. values: [16, 2048]	256
floatgrid	max_delta_step	Maximum delta step allowed for each tree's weight estimation. If the value is set to 0, there is no constraint. Setting to a positive value makes the update step more conservative. Usually this parameter is not needed, but it might help in logistic regression when class is extremely imbalanced. Setting it to a value of 1-10 might help control the delta step update. values: [0,100]	0.0
intgrid	max_depth	Maximum depth of the individual regression estimators. The maximum depth limits the number of nodes in the tree. Tune this parameter for optimal performance; the best value depends on the interaction of the input variables. The deeper the tree, the more variable interactions the model can capture. For frozen models that have larger sample sizes than the parent model, the max_depth value is increased to retain similar accuracy. values: [1, 16]	8
floatgrid	min_child_weight	Minimum sum of instance weight (hessian) needed in a child. If the tree partition step results in a leaf node with the sum of instance weight less than min_child_weight, the building process will give up further partitioning. In linear regression mode, this simply corresponds to the minimum number of instances needed to be in each node. The larger the value, the more conservative the algorithm will be. values: [0.01,float(1e5)]	1.0
floatgrid	min_split_loss	Minimum loss reduction required to make a further partition on a leaf node of the tree. The larger the value, the more conservative the algorithm will be. values: [0,1e5]	0.01

float	missing_value	Float value that should be treated as a missing value. When mono_up or mono_down is set, missing value will be set to -9999.0. values: [float(-1e5),float(1e5)]	-9999.0
string	mono_down	ID of the featurelist that defines the set of features with a monotonically decreasing relationship to the target.	None
string	mono_up	ID of the featurelist that defines the set of features with a monotonically increasing relationship to the target.	None
int	n_estimators	Number of boosting stages to perform. Gradient boosting is fairly robust to overfitting, so a larger number usually results in better performance. values: [1,20000]	480
intgrid	num_parallel_tree	Number of parallel trees created in each boosting stage. When this value is greater than 1, the model becomes a gradient-boosted random forest with (num_parallel_tree * n_estimators) trees. values: [1,16]	1
intgrid	random_state	Seed used in the random number generator 'values': [0, int(1e9)]	1234
multi	reg_alpha	L1 regularization term on weights; increasing this value will make the model more conservative. values: {'floatgrid': [0, 1e6], 'select': ['auto']}	0.0
multi	reg_lambda	L2 regularization term on weights. Increasing this value will make the model more conservative. values: {'floatgrid': [0, 1e6], 'select': ['auto']}	1.0
float	scale_pos_weight	Scaling factor for examples in the positive class. values: [0,float(1e9)]	1.0
floatgrid	subsample	Subsample ratio of the training instance. Setting it to 0.5 means that XGBoost randomly collected half of the data instances to grow trees, which will prevent overfitting.	1.0
select	tree_method	Tree construction algorithm to use. 'auto': Heuristic to choose the faster algortihm. For small to medium datasets (<4M rows), exact greedy will be used. For large datasets (>=4M rows), approximate algorithm will be used. 'exact':Exact greedy algorithm. 'approx':Approximate greedy algorithm using sketching and histogram. 'hist':Fast histogram-optimized approximate greedy algorithm. It uses some performance improvements (e.g., bin caching). values: ['auto', 'exact', 'approx', 'hist']	auto

Eureqa Classifier (Default Search 3000 Generations)

One-Hot Encoding Task

Type	Name	Description	Best Searched
int	card_max	An integer that specifies the maximum number of unique values. values: [1, 99999]	50000

int	card_min	An integer that specifies the minimum number of unique values. values: [1, 99999]	1
bool	drop_cols	drop_cols, If True, drop last level of each feature values: [False, True]	False
select	flag	flag, If all, add highcat-cols to metadata values: ['None', 'all']	None
int	max_features	If the total number of categories created across all features exceeds this value, the top max_features most frequent categories will persist. All others will be either thrown out or grouped. A value of None disables the limit. values: [1, 999999]	20000
int	min_support	The minimum number of records for a category to be represented in one hot encoding. If a category has fewer counts it will be grouped with other small cardinality values. values: [1, 99999]	10

Median Value-Based Numeric Imputation (V2 with quick median algorithm)

Type	Name	Description	Best Searched
bool	scale_small	True if small values (range of the numeric variable is <= 1) are to be scaled. values: [False, True]	False
int	threshold	Minimum number of required finite elements in a column to impute the data onto NaNs and INFs. values: [1, 99999]	10

Feature Selection For Dimensionality Reduction

Type	Name	Description	Best Searched
int	FeatureSelection: max_features	The maximum number of features to include. ``values: {'int': [1, int(1e10)], 'select': ['no_limit']}`	10
select	FeatureSelection: method	Method used to select features values: ['per_variable', 'cumulative', 'no_selection']	per_variable
int	FeatureSelection: min_features	The minimum number of features to include. values: [1, int(1e5)]	1
float	FeatureSelection: threshold	The threshold used to select features. For cumulative method, threshold is typically close to 1 in order to retain more signal (ex:0.98). For per_variable method, threshold would be close to 0 in order to discard only features with low signal. values: [0.0001, 1]	0.001

Prediction Model Parameters: Eureqa Classifier

Type	Name	Description	Best Searched
int or select	building_block__absolute_value	'Absolute Value' building block. Allows Eureqa to use the abs() operator in model expressions, and sets its complexity penalty. Usage: abs(x). Returns the positive value of x, without regard for its sign. values: [0, 100] or "Disabled".	Disabled

int or select	building_block__addition	‘Addition’ building block. Allows Eureqa to use the + operator in model expressions, and sets its complexity penalty. Usage: x + y or add(x, y), which returns the sum of x and y. values: [0, 100] or "Disabled".	0
int or select	building_block__arccosine	‘Arccosine’ building block. Allows Eureqa to use the acos() operator in model expressions, and sets its complexity penalty. Usage: acos(x). Returns the standard trigonometric arccosine of x. values: [0, 100] or "Disabled".	Disabled
int or select	building_block__arcsine	‘Arcsine’ building block. Allows Eureqa to use the asin() operator in model expressions, and sets its complexity penalty. Usage: asin(x). Returns the standard trigonometric arcsine of x. values: [0, 100] or "Disabled".	Disabled
int or select	building_block__arctangent	‘Arctangent’ building block. Allows Eureqa to use the atan() operator in model expressions, and sets its complexity penalty. Usage: atan(x). Returns the standard trigonometric arctangent of x. values: [0, 100] or "Disabled".	Disabled
int or select	building_block__ceiling	‘Ceiling’ building block. Allows Eureqa to use the ceil() operator in model expressions, and sets its complexity penalty. Usage: ceil(x), which returns the smallest integer not less than x. values: [0, 100] or "Disabled".	Disabled
int or select	building_block__complementary_error_function	‘Complementary Error Function’ building block. Allows Eureqa to use the erfc() operator in model expressions, and sets its complexity penalty. Usage: erfc(x). This is defined as 1.0 - erf(x), where erf(x) is the integral of the normal distribution, and returns a value between 2 and 0. values: [0, 100] or "Disabled".	Disabled
int or select	building_block__constant	‘Constant’ building block. Allows Eureqa to use constants in model expressions, and sets their complexity penalty. Usage: c, where c is a real valued constant. values: [0, 100] or "Disabled"	0
int or select	building_block__cosine	‘Cosine’ building block. Allows Eureqa to use the cos() operator in model expressions, and sets its complexity penalty. Usage: cos(x), where the angle x is in radians. Returns the standard trigonometric cosine of x. values: [0, 100] or "Disabled".	Disabled
int or select	building_block__division	‘Division’ building block. Allows Eureqa to use the / operator in model expressions, and sets its complexity penalty. Usage: x / y or div(x, y), which returns the quotient of x and y (where y must be non-zero). values: [0, 100] or "Disabled".	2
int or select	building_block__equal-to	‘Equal-To’ building block. Allows Eureqa to use the = operator in model expressions, and sets its complexity penalty. Usage: equal(x, y) or x = y, which returns 1 if x is numerically equal to y, 0 otherwise. values: [0, 100] or "Disabled".	Disabled
int or select	building_block__error_function	‘Error Function’ building block. Allows Eureqa to use the erf() operator in model expressions, and sets its complexity penalty. It is defined as the integral of the normal distribution. Usage: erf(x), which returns a value	Disabled

		between -1 and +1. values: [0, 100] or "Disabled".	
int or select	building_block__exponential	'Exponential' building block. Allows Eureqa to use the exp() operator in model expressions, and sets its complexity penalty. Usage: exp(x), which returns e raised to the power x. values: [0, 100] or "Disabled".	Disabled
int or select	building_block__factorial	'Factorial' building block. Allows Eureqa to use the ! operator in model expressions, and sets its complexity penalty. Usage: factorial(x) or x!, which returns the product of all positive integers from 1 to x. values: [0, 100] or "Disabled".	Disabled
int or select	building_block__floor	'Floor' building block. Allows Eureqa to use the floor() operator in model expressions, and sets its complexity penalty. Usage: floor(x), which returns the largest integer not greater than x. values: [0, 100] or "Disabled".	Disabled
int or select	building_block__gaussian_function	'Gaussian Function' building block. Allows Eureqa to use the gauss()" operator in model expressions, and sets its complexity penalty. Usage: gauss(x), which returns exp(-x^2). This is a bell-shaped squashing function. values: [0, 100] or "Disabled".	Disabled
int or select	building_block__greater-than	'Greater-Than' building block. Allows Eureqa to use the > operator in model expressions, and sets its complexity penalty. Usage: greater(x, y) or x > y, which returns 1 if x > y, 0 otherwise. values: [0, 100] or "Disabled".	Disabled
int or select	building_block__greater-than-or-equal	'Greater-Than-Or-Equal' building block. Allows Eureqa to use the >= operator in model expressions, and sets its complexity penalty. Usage: greater_or_equal(x, y) or x >= y, which returns 1 if x >= y, 0 otherwise. values: [0, 100] or "Disabled".	Disabled
int or select	building_block__hyperbolic_cosine	'Hyperbolic Cosine' building block. Allows Eureqa to use the cosh() operator in model expressions, and sets its complexity penalty. Usage: cosh(x). Returns the standard trigonometric hyperbolic cosine of x. values: [0, 100] or "Disabled".	Disabled
int or select	building_block__hyperbolic_sine	'Hyperbolic Sine' building block. Allows Eureqa to use the sinh() operator in model expressions, and sets its complexity penalty. Usage: sinh(x). Returns the standard trigonometric hyperbolic sine of x. values: [0, 100] or "Disabled".	Disabled
int or select	building_block__hyperbolic_tangent	'Hyperbolic Tangent' building block. Allows Eureqa to use the tanh() operator in model expressions, and sets its complexity penalty. Usage: tanh(x). Returns the hyperbolic tangent of x. Hyperbolic tangent is a common squashing function that returns a value between -1 and +1. values: [0, 100] or "Disabled".	Disabled
int or select	building_block__if-then-else	'If-Then-Else' building block. Allows Eureqa to use the if() operator in model expressions, and sets its complexity penalty. Usage: if(x, y, z), which returns y if x is greater than 0, z otherwise; if x is null, the function returns z. values: [0, 100] or "Disabled".	1
int or select	building_block__input_variable	'Input Variable' building block. Allows Eureqa to use variables in model	1

		expressions, and sets their complexity penalty. Usage: x, where x is a variable in your prepared dataset. values: [0, 100] or "Disabled"	
int or select	building_block__integer_constant	‘Integer Constant’ building block. Allows Eureqa to use integer constants in model expressions, and sets their complexity penalty. Usage: c, where c is an integer constant. values: [0, 100] or "Disabled"	Disabled
int or select	building_block__inverse_hyperbolic_cosine	‘Inverse Hyperbolic Cosine’ building block. Allows Eureqa to use the acosh() operator in model expressions, and sets its complexity penalty. Usage: acosh(x). Returns the standard inverse hyperbolic cosine of x. values: [0, 100] or "Disabled".	Disabled
int or select	building_block__inverse_hyperbolic_sine	‘Inverse Hyperbolic Sine’ building block. Allows Eureqa to use the asinh() operator in model expressions, and sets its complexity penalty. Usage: asinh(x). Returns the standard inverse hyperbolic sine of x. values: [0, 100] or "Disabled".	Disabled
int or select	building_block__inverse_hyperbolic_tangent	‘Inverse Hyperbolic Tangent’ building block. Allows Eureqa to use the atanh() operator in model expressions, and sets its complexity penalty. Usage: atanh(x). Returns the standard inverse hyperbolic tangent of x. values: [0, 100] or "Disabled".	Disabled
int or select	building_block__less-than	‘Less-Than’ building block. Allows Eureqa to use the < operator in model expressions, and sets its complexity penalty. Usage: less(x, y) or x < y, which returns 1 if x < y, 0 otherwise. values: [0, 100] or "Disabled".	1
int or select	building_block__less-than-or-equal	‘Less-Than-Or-Equal’ building block. Allows Eureqa to use the <= operator in model expressions, and sets its complexity penalty. Usage: less_or_equal(x, y) or x <= y, which returns 1 if x <= y, 0 otherwise. values: [0, 100] or "Disabled".	Disabled
int or select	building_block__logical_and	‘Logical And’ building block. Allows Eureqa to use the and operator in model expressions, and sets its complexity penalty. Usage: and(x, y), which returns 1 if both x and y are greater than 0, 0 otherwise. values: [0, 100] or "Disabled".	Disabled
int or select	building_block__logical_not	‘Logical Not’ building block. Allows Eureqa to use the not operator in model expressions, and sets its complexity penalty. Usage: not(x), which returns 0 if x is greater than 0, 1 otherwise. values: [0, 100] or "Disabled".	Disabled
int or select	building_block__logical_or	‘Logical Or’ building block. Allows Eureqa to use the or operator in model expressions, and sets its complexity penalty. Usage: or(x, y), which returns 1 if either x or y are greater than 0, 0 otherwise. values: [0, 100] or "Disabled".	Disabled
int or select	building_block__logical_xor	‘Logical Xor’ building block. Allows Eureqa to use the xor operator in model expressions, and sets its complexity penalty. Usage: xor(x, y), which returns 1 if (x <= 0 and y > 0) or (x > 0 and y <= 0), 0 otherwise. values: [0, 100] or "Disabled".	Disabled
int or select	building_block__logistic_function	‘Logistic Function’ building block. Allows Eureqa to use the logistic() operator in model expressions, and sets	4

		its complexity penalty. Usage: logistic(x), which returns 1/(1 + exp(-x)). This is a common sigmoid (s-shaped) squashing function that returns a value between 0 and 1. values: [0, 100] or "Disabled".	
int or select	building_block__maximum	'Maximum' building block. Allows Eureqa to use the max() operator in model expressions, and sets its complexity penalty. Usage: max(x, y), which returns the maximum (signed) result of x and y. values: [0, 100] or "Disabled".	1
int or select	building_block__minimum	'Minimum' building block. Allows Eureqa to use the min() operator in model expressions, and sets its complexity penalty. Usage: min(x, y), which returns the minimum (signed) result of x and y. values: [0, 100] or "Disabled".	1
int or select	building_block__modulo	'Modulo' building block. Allows Eureqa to use the mod() operator in model expressions, and sets its complexity penalty. Usage: mod(x, y), which returns the remainder of x / y. values: [0, 100] or "Disabled".	Disabled
int or select	building_block__multiplication	'Multiplication' building block. Allows Eureqa to use the * operator in model expressions, and sets its complexity penalty. Usage: x * y or mul(x, y), which returns the product of x and y. values: [0, 100] or "Disabled".	0
int or select	building_block__natural_logarithm	'Natural Logarithm' building block. Allows Eureqa to use the log() operator in model expressions, and sets its complexity penalty. Usage: log(x), which returns the natural logarithm (base e) of x. values: [0, 100] or "Disabled".	2
int or select	building_block__negation	'Negation' building block. Allows Eureqa to use the - unary operator in model expressions, and sets its complexity penalty. Usage: -x, which returns the inverse of x. values: [0, 100] or "Disabled".	Disabled
int or select	building_block__power	'Power' building block. Allows Eureqa to use the ^ operator in model expressions, and sets its complexity penalty. Usage: x^y, which returns x raised to the power y. values: [0, 100] or "Disabled".	Disabled
int or select	building_block__round	'Round' building block. Allows Eureqa to use the round() operator in model expressions, and sets its complexity penalty. Usage: round(x), which returns x rounded to the nearest integer. values: [0, 100] or "Disabled".	Disabled
int or select	building_block__sign_function	'Sign Function' building block. Allows Eureqa to use the sign() operator in model expressions, and sets its complexity penalty. Usage: sgn(x), which returns -1 if x is negative, +1 if x is positive, and 0 if x is zero. values: [0, 100] or "Disabled".	Disabled
int or select	building_block__sine	'Sine' building block. Allows Eureqa to use the sin() operator in model expressions, and sets its complexity penalty. Usage: sin(x), where the angle x is in radians. Returns the standard trigonometric sine of x. values: [0, 100] or "Disabled".	Disabled
int or select	building_block__square_root	'Square Root' building block. Allows Eureqa to use the sqrt() operator in model expressions, and sets its	1

		complexity penalty. Usage: sqrt(x), which returns the square root of x (where x must be positive). values: [0, 100] or "Disabled".	
int or select	building_block__step_function	‘Step Function’ building block. Allows Eureqa to use the step() operator in model expressions, and sets its complexity penalty. Usage: step(x), which returns 1 if x is positive, 0 otherwise. values: [0, 100] or "Disabled".	2
int or select	building_block__subtraction	‘Subtraction’ building block. Allows Eureqa to use the - binary operator in model expressions, and sets its complexity penalty. Usage: x - y or sub(x, y), which returns the difference of x and y. values: [0, 100] or "Disabled".	0
int or select	building_block__tangent	‘Tangent’ building block. Allows Eureqa to use the tan() operator in model expressions, and sets its complexity penalty. Usage: tan(x), where the angle x is in radians. Returns the standard trigonometric tangent of x. values: [0, 100] or "Disabled".	Disabled
int or select	building_block__two-argument_arctangent	‘Two-Argument Arctangent’ building block. Allows Eureqa to use the atan2() operator in model expressions, and sets its complexity penalty. Usage: atan2(y, x). Returns the standard trigonometric two-argument arctangent of x and y. values: [0, 100] or "Disabled".	Disabled
select	error_metric	Which Error Metric Eureqa should use internally, to evaluate which models to keep on its internal Pareto Front. This may be different from the metric that DataRobot uses for evaluating error on the validation set. values: [Mean_Absolute_Error, Mean_Absolute_Percentage_Error, Mean_Squared_Error, Root_Mean_Squared_Error, R^2_Goodness_of_Fit, Correlation_Coefficient, Maximum_Absolute_Error, Mean_Logarithm_Squared_Error, Median_Absolute_Error, Interquartile_Mean_Absolute_Error, AIC_Squared_Error, AIC_Absolute_Error, Rank_Correlation, Hinge_Loss_Error, Slope_Absolute_Error]	Log_Loss_Error
int	max_generations	Maximum number of evolutionary generations to run. Eureqa will run until either of max_generations or timeout_sec is reached. values: [0, 1e16]	3000
int	num_threads	Number of threads Eureqa will run with. Ideally equal to the number of cores available.	4
string	prior_solutions	Prior Eureqa Solutions. This field contains multiple Eureqa Expressions, one per line. Each Expression should be a valid Eureqa Solution, such as a Solution returned by a previous run of Eureqa. (You may need to edit the Solution such that the target variable is entered as “Target”, not the original column name of the target.) Each expression is fed into Eureqa’s initial evolutionary population. Eureqa makes no guarantees about keeping the form or content of these expressions in the final Pareto front of expressions that it generates, but if the expressions are good models or if they contain sub-expressions that are predictive features, Eureqa will generally take advantage of that information to converge on good solutions more quickly. values: multiple	

		lines, each line is a valid Eureqa Expression as a string	
int	random_seed	Constant to seed Eureqa's pseudo-random number generator. Different values will cause Eureqa to generate different models on the same data and other input parameters. values: [0, 1e16]	31337
select	split_mode	Whether to perform in-order (2) or random (1) splitting within the training set, for evolutionary re-training and re-validation. values: [1, 2]	1
select	sync_migrations	Should Eureqa's migrations be synchronized? If they are synchronized, Eureqa's fit() function will be deterministic (repeated runs on the same data and parameters should produce the same models). Note that synchronization slows modeling down and models will take more time to generate. values: [False, True]	True
string	target_expression_string	Eureqa Target Expression. Constrains the form of the models that Eureqa will consider. This field is typically of the form Target = <some Eureqa Expression>. See the description of "Eureqa Expressions". The Expression must contain an equality operator, ie., it must be a full equation with a left-hand and a right-hand side. Note that Target Expressions will usually use function operators in their expressions, as well as regular expression operators. Function operators are templates / pattern matchers. For example, Target = f(x, y z) will match any expression that uses the variables x, y, and/or z (but will fail to match any function that uses other variables); Target = f1(x, y) + f2(z) will match x*y + z but it will not match x*z + y; Target = f(sin(x)) will match 2*sin(x) but it will not match sin(2*x). This is a hard constraint. If you want to seed Eureqa with information from existing models, see prior_solutions. If this field is left blank, Eureqa will automatically generate an appropriate target expression as part of the model fitting process. This expression will be logged to the model log (visible in the UI). Any column that has non ISO-8859 (Latin-1) characters in its column name must be enclosed in parentheses. The same is true for any one-hot-encoded categorical column of any encoding that contains key-value pair in their names. For example, the expression Target = ローン額*10 - 100*department-chemistry would be written as Target = (ローン額)*10 - 100*(department-chemistry). values: valid Eureqa Expression, as a string. Must contain an equality operator.	
float	timeout_sec	Maximum length of time to run the Eureqa search algorithm. Eureqa will run until either of max_generations or timeout_sec is reached. values: [0, 1e16]	1000000.0
float	training_fraction	What fraction of the DataRobot training data to use for Eureqa evolutionary training? This field is ignored if training_split_expression is set. Note that training_fraction + validation_fraction does not have to equal 1.0. If it is less than 1.0, some rows in the data are ignored. If it is greater than 1.0, Eureqa's training and validation sets overlap. (This is not recommended, but may be required in some modeling scenarios involving small datasets. Note that DataRobot will	0.5

		still typically have a separate validation and holdout set.) values: [0.0, 1.0]	
string	training_split_expr	Eureqa Training Split Expression. Can be any valid Eureqa Expression, including a simple variable name. The expression should evaluate to either 0.0 or 1.0. If it evaluates to 1.0 on a specific row, that row is used for training data. If this field is not set, training_fraction is used instead. Note that setting training_split_expr does not affect validation rows. If you want all non-training rows to be in the validation set, make sure to set validation_split_expr accordingly. values: valid Eureqa Expression, as a string	
float	validation_fraction	What fraction of the DataRobot training data to use for Eureqa evolutionary validation? This field is ignored if validation_split_expression is set. Note that training_fraction + validation_fraction does not have to equal 1.0. If it is less than 1.0, some rows in the data are ignored. If it is greater than 1.0, Eureqa's training and validation sets overlap. (This is not recommended, but may be required in some modeling scenarios involving small datasets. Note that DataRobot will still typically have a separate validation and holdout set.) values: [0.0, 1.0]	0.5
string	validation_split_expr	Eureqa Validation Split Expression. Can be any valid Eureqa Expression, including a simple variable name. The expression should evaluate to either 0.0 or 1.0. If it evaluates to 1.0 on a specific row, that row is used for validation data. If this field is not set, validation_fraction is used instead. Note that setting validation_split_expr does not impact training rows. If you want all non-validation rows to be in the training set, make sure to set training_split_expr accordingly. values: valid Eureqa Expression, as a string	
string	weight_expr	Eureqa Weight Expression. Weights each row when evaluating the error of that row. The exact meaning of the weight depends on the specific value of error_metric. Typically the per-row error is multiplied by the weight before being combined with other per-row weights into the aggregate weight. If this field is left as the empty string, Eureqa falls back to DataRobot's default behavior (the project weights are used). Columns that are used in the project as offset, exposure, or weight cannot be used in a weights expression. To use project weights, the duplicate weights column must be available in the modeling columns. Given a weight column weight, for example, you can create a column weight_modeling and then use that column in the weigh expression (for example 0.3 * weight_modeling * weight_modeling). values: valid Eureqa Expression, as a string	

Target 3: AVG Blender (Eureqa Generalized Additive Model Classifier (40 Generations), eXtreme Gradient Boosted Trees Classifier with Early Stopping, Dropout Additive Regression Trees Classifier (15 leaves))

Eureqa Generalized Additive Model Classifier (40 Generations)

Median Value-Based Numeric Imputation (V2 with quick median algorithm)

Type	Name	Description	Best Searched
bool	scale_small	True if small values (range of the numeric variable is <= 1) are to be scaled. values: [False, True]	False
int	threshold	Minimum number of required finite elements in a column to impute the data onto NaNs and INFs. values: [1, 99999]	10

One-Hot Encoding Task

Type	Name	Description	Best Searched
int	card_max	An integer that specifies the maximum number of unique values. values: [1, 99999]	10000
int	card_min	An integer that specifies the minimum number of unique values. values: [1, 99999]	11
bool	drop_cols	drop_cols, If True, drop last level of each feature values: [False, True]	False
select	flag	flag, If all, add highcat-cols to metadata values: ['None', 'all']	all
int	max_features	If the total number of categories created across all features exceeds this value, the top max_features most frequent categories will persist. All others will be either thrown out or grouped. A value of None disables the limit. values: [1, 999999]	None
int	min_support	The minimum number of records for a category to be represented in one hot encoding. If a category has fewer counts it will be grouped with other small cardinality values. values: [1, 99999]	5

Elasticnet Classifier model based on block coordinate descent

Type	Name	Description	Best Searched
select	beta_transform	beta_transform is a parameter used for blenders. If beta_transform is set to 'blender', coefficients are non-negative and are all in [0, 1]. Very large weight of the penalty term will get you the average blender. values: ['id', 'blender']	id
multi	enet_alpha	The ElasticNet mixing parameter, with 0 <= alpha <= 1. For alpha = 0 the penalty is an L2 penalty. For alpha = 1 it is an L1 penalty. For 0 < alpha < 1, the penalty is a combination of L1 and L2. 'auto' grid of 11 numbers spaced evenly from 0.0 to 1.0 values: {'floatgrid': [0, 1], 'select': ['auto']}	0.0
multi	enet_lambda	The weight for the penalty term 'auto' searches a grid of 50 numbers spaced evenly on a log10 scale from 3.16e-07 to	auto

		3.16e-01 values: {'floatgrid':[1e-10,1e10],'select':['auto']}	
bool	fit_alpha_scaler	If it is set to True, the weight of the penalty term is scaled. A weight of 1 would lead to an intercept only model and a weight of 0 won't apply any penalty. With this parameter set to True, a penalty > 1 would not make sense any more. values: [False, True]	True
bool	fit_intercept	whether to calculate the intercept for this model. If set to false, no intercept will be used in calculations (e.g. data is expected to be already centered). values: [False, True]	True
select	loss	The loss function to be used. values: ['log']	log
int	max_iter	The maximum number of iterations values: [1, 1e6]	100
int	random_state	The seed of the pseudo random number generator to use. values: [0, int(1e9)]	1234
float	sigma	Constant used in the line search sufficient decrease condition. values': [0, 1e-6]	1e-06
float	tol	The tolerance for the optimization: if the updates are smaller than tol, the optimization code checks the dual gap for optimality and continues until it is smaller than tol. values: [1e-10, 1e10]	0.0001
bool	warm_start	When set to True, reuse the solution of the previous call to fit as initialization, otherwise, just erase the previous solution. values: [False, True]	False

Prediction Model Parameters Generalized Additive Model Classifier

Type	Name	Description	Best Searched
int or select	EUREQA_building_block__absolute_value	'Absolute Value' building block. Allows Eureqa to use the "abs()" operator in model expressions, and sets its complexity penalty. Usage: abs(x), which returns the positive value of x, without regard for its sign. values: [0, 100] or "Disabled".	None
int or select	EUREQA_building_block__addition	'Addition' building block. Allows Eureqa to use the "+" operator in model expressions, and sets its complexity penalty. Usage: x + y or add(x, y), which returns the sum of x and y. values: [0, 100] or "Disabled".	None
int or select	EUREQA_building_block__arccosine	'Arccosine' building block. Allows Eureqa to use the "acos()" operator in model expressions, and sets its complexity penalty. Usage: acos(x). (The standard trigonometric arccosine function.) values: [0, 100] or "Disabled".	None
int or select	EUREQA_building_block__arcsine	'Arcsine' building block. Allows Eureqa to use the "asin()" operator in model expressions, and sets its complexity penalty. Usage: asin(x). (The standard trigonometric arcsine function.) values: [0, 100] or "Disabled".	None
int or select	EUREQA_building_block__arctangent	'Arctangent' building block. Allows Eureqa to use the "atan()" operator in model expressions, and sets its complexity penalty. Usage: atan(x). (The standard trigonometric	None

		arctangent function.) values: [0, 100] or "Disabled".	
int or select	EUREQA_building_block__ceiling	‘Ceiling’ building block. Allows Eureqa to use the “ceil()” operator in model expressions, and sets its complexity penalty. Usage: ceil(x), which returns the smallest integer not less than x. values: [0, 100] or "Disabled".	None
int or select	EUREQA_building_block__complementary_error_function	‘Complementary Error Function’ building block. Allows Eureqa to use the “erfc()” operator in model expressions, and sets its complexity penalty. Usage: erfc(x). 1.0 - erf(x) where erf(x) is the integral of the normal distribution and returns a value between 2 and 0. values: [0, 100] or "Disabled".	None
int or select	EUREQA_building_block__constant	‘Constant’ building block. Allows Eureqa to use constants in model expressions, and sets their complexity penalty. Usage: c, where c is a real valued constant. values: [0, 100] or "Disabled"	None
int or select	EUREQA_building_block__cosine	‘Cosine’ building block. Allows Eureqa to use the “cos()” operator in model expressions, and sets its complexity penalty. Usage: cos(x), where the angle (x) is in radians. (The standard trigonometric cosine function.) values: [0, 100] or "Disabled".	None
int or select	EUREQA_building_block__division	‘Division’ building block. Allows Eureqa to use the “/” operator in model expressions, and sets its complexity penalty. Usage: x / y or div(x, y), which returns the quotient of x and y (where y must be non-zero). values: [0, 100] or "Disabled".	None
int or select	EUREQA_building_block__equal-to	‘Equal-To’ building block. Allows Eureqa to use the “=” operator in model expressions, and sets its complexity penalty. Usage: equal(x, y) or x = y, which returns 1 if x is numerically equal to y, 0 otherwise. values: [0, 100] or "Disabled".	None
int or select	EUREQA_building_block__error_function	‘Error Function’ building block. Allows Eureqa to use the “erf()” operator in model expressions, and sets its complexity penalty. Usage: erf(x). Integral of the normal distribution; returns a value between -1 and +1. values: [0, 100] or "Disabled".	None
int or select	EUREQA_building_block__exponential	‘Exponential’ building block. Allows Eureqa to use the “exp()” operator in model expressions, and sets its complexity penalty. Usage: exp(x), which returns e^x. values: [0, 100] or "Disabled".	None
int or select	EUREQA_building_block__factorial	‘Factorial’ building block. Allows Eureqa to use the “!” operator in model expressions, and sets its complexity penalty. Usage: factorial(x) or x!, which returns the product of all positive integers from 1 to x. values: [0, 100] or "Disabled".	None
int or select	EUREQA_building_block__floor	‘Floor’ building block. Allows Eureqa to use the “floor()” operator in model expressions, and sets its complexity penalty. Usage: floor(x), which returns the largest integer not greater than x. values: [0, 100] or "Disabled".	None

int or select	EUREQA_building_block__gaussian_function	‘Gaussian Function’ building block. Allows Eureqa to use the “gauss()” operator in model expressions, and sets its complexity penalty. Usage: gauss(x), which returns exp(-x^2). This is a bell-shaped squashing function. values: [0, 100] or "Disabled".	None
int or select	EUREQA_building_block__greater-than	‘Greater-Than’ building block. Allows Eureqa to use the “>” operator in model expressions, and sets its complexity penalty. Usage: greater(x, y) or x > y, which returns 1 if x > y, 0 otherwise. values: [0, 100] or "Disabled".	None
int or select	EUREQA_building_block__greater-than-or-equal	‘Greater-Than-Or-Equal’ building block. Allows Eureqa to use the “>=” operator in model expressions, and sets its complexity penalty. Usage: greater_or_equal(x, y) or x >= y, which returns 1 if x >= y, 0 otherwise. values: [0, 100] or "Disabled".	None
int or select	EUREQA_building_block__hyperbolic_cosine	‘Hyperbolic Cosine’ building block. Allows Eureqa to use the “cosh()” operator in model expressions, and sets its complexity penalty. Usage: cosh(x). (The standard trigonometric hyperbolic cosine function.) values: [0, 100] or "Disabled".	None
int or select	EUREQA_building_block__hyperbolic_sine	‘Hyperbolic Sine’ building block. Allows Eureqa to use the “sinh()” operator in model expressions, and sets its complexity penalty. Usage: sinh(x). (The standard trigonometric hyperbolic sine function.) values: [0, 100] or "Disabled".	None
int or select	EUREQA_building_block__hyperbolic_tangent	‘Hyperbolic Tangent’ building block. Allows Eureqa to use the “tanh()” operator in model expressions, and sets its complexity penalty. Usage: tanh(x). (The hyperbolic tangent of x.) Hyperbolic tangent is a common squashing function that returns a value between -1 and +1. values: [0, 100] or "Disabled".	None
int or select	EUREQA_building_block__if-then-else	‘If-Then-Else’ building block. Allows Eureqa to use the “if()” operator in model expressions, and sets its complexity penalty. Usage: if(x, y, z), which returns y if x is greater than 0, z otherwise; if x is nan, the function returns z. values: [0, 100] or "Disabled".	None
int or select	EUREQA_building_block__input_variable	‘Input Variable’ building block. Allows Eureqa to use variables in model expressions, and sets their complexity penalty. Usage: x, where x is a variable in your prepared dataset. values: [0, 100] or "Disabled"	None
int or select	EUREQA_building_block__integer_constant	‘Integer Constant’ building block. Allows Eureqa to use integer constants in model expressions, and sets their complexity penalty. Usage: c, where c is an integer constant. values: [0, 100] or "Disabled"	None
int or select	EUREQA_building_block__inverse_hyperbolic_cosine	‘Inverse Hyperbolic Cosine’ building block. Allows Eureqa to use the “acosh()” operator in model expressions, and sets its complexity penalty. Usage: acosh(x). (The standard inverse hyperbolic cosine function.) values: [0, 100] or "Disabled".	None

int or select	EUREQA_building_block__inverse_hyperbolic_sine	'Inverse Hyperbolic Sine' building block. Allows Eureqa to use the "asinh()" operator in model expressions, and sets its complexity penalty. Usage: asinh(x). (The standard inverse hyperbolic sine function.) values: [0, 100] or "Disabled".	None
int or select	EUREQA_building_block__inverse_hyperbolic_tangent	'Inverse Hyperbolic Tangent' building block. Allows Eureqa to use the "atanh()" operator in model expressions, and sets its complexity penalty. Usage: atanh(x). (The standard inverse hyperbolic tangent function.) values: [0, 100] or "Disabled".	None
int or select	EUREQA_building_block__less-than	'Less-Than' building block. Allows Eureqa to use the "<" operator in model expressions, and sets its complexity penalty. Usage: less(x, y) or x < y, which returns 1 if x < y, 0 otherwise. values: [0, 100] or "Disabled".	None
int or select	EUREQA_building_block__less-than-or-equal	'Less-Than-Or-Equal' building block. Allows Eureqa to use the "<=" operator in model expressions, and sets its complexity penalty. Usage: less_or_equal(x, y) or x <= y, which returns 1 if x <= y, 0 otherwise. values: [0, 100] or "Disabled".	None
int or select	EUREQA_building_block__logical_and	'Logical And' building block. Allows Eureqa to use the "and" operator in model expressions, and sets its complexity penalty. Usage: and(x, y), which returns 1 if both x and y are greater than 0, 0 otherwise. values: [0, 100] or "Disabled".	None
int or select	EUREQA_building_block__logical_not	'Logical Not' building block. Allows Eureqa to use the "not" operator in model expressions, and sets its complexity penalty. Usage: not(x), which returns 0 if x is greater than 0, 1 otherwise. values: [0, 100] or "Disabled".	None
int or select	EUREQA_building_block__logical_or	'Logical Or' building block. Allows Eureqa to use the "or" operator in model expressions, and sets its complexity penalty. Usage: or(x, y), which returns 1 if either x or y are greater than 0, 0 otherwise. values: [0, 100] or "Disabled".	None
int or select	EUREQA_building_block__logical_xor	'Logical Xor' building block. Allows Eureqa to use the "xor" operator in model expressions, and sets its complexity penalty. Usage: xor(x, y), which returns 1 if (x <= 0 and y > 0) or (x > 0 and y <= 0), 0 otherwise. values: [0, 100] or "Disabled".	None
int or select	EUREQA_building_block__logistic_function	'Logistic Function' building block. Allows Eureqa to use the "logistic()" operator in model expressions, and sets its complexity penalty. Usage: logistic(x), which returns 1/(1 + exp(-x)). This is a common sigmoid (s-shaped) squashing function that returns a value between 0 and 1. values: [0, 100] or "Disabled".	None
int or select	EUREQA_building_block__maximum	'Maximum' building block. Allows Eureqa to use the "max()" operator in model expressions, and sets its complexity penalty. Usage: max(x, y), which returns the maximum (signed) result of x and y. values: [0, 100] or "Disabled".	None

int or select	EUREQA_building_block__minimum	'Minimum' building block. Allows Eureqa to use the "min()" operator in model expressions, and sets its complexity penalty. Usage: min(x, y), which returns the minimum (signed) result of x and y. values: [0, 100] or "Disabled".	None
int or select	EUREQA_building_block__modulo	'Modulo' building block. Allows Eureqa to use the "mod()" operator in model expressions, and sets its complexity penalty. Usage: mod(x, y), which returns the remainder of x / y. values: [0, 100] or "Disabled".	None
int or select	EUREQA_building_block__multiplication	'Multiplication' building block. Allows Eureqa to use the "*" operator in model expressions, and sets its complexity penalty. Usage: x * y or mul(x, y), which returns the product of x and y. values: [0, 100] or "Disabled".	None
int or select	EUREQA_building_block__natural_logarithm	'Natural Logarithm' building block. Allows Eureqa to use the "log()" operator in model expressions, and sets its complexity penalty. Usage: log(x), which returns the natural logarithm (base e) of x. values: [0, 100] or "Disabled".	None
int or select	EUREQA_building_block__negation	'Negation' building block. Allows Eureqa to use the "-" unary operator in model expressions, and sets its complexity penalty. Usage: -x, which returns the inverse of x. values: [0, 100] or "Disabled".	None
int or select	EUREQA_building_block__power	'Power' building block. Allows Eureqa to use the "^" operator in model expressions, and sets its complexity penalty. values: [0, 100] or "Disabled".	None
int or select	EUREQA_building_block__round	'Round' building block. Allows Eureqa to use the "round()" operator in model expressions, and sets its complexity penalty. Usage: round(x), which returns an integer of x rounded to the nearest integer. values: [0, 100] or "Disabled".	None
int or select	EUREQA_building_block__sign_function	'Sign Function' building block. Allows Eureqa to use the "sign()" operator in model expressions, and sets its complexity penalty. Usage: sgn(x), which returns -1 if x is negative, +1 if x is positive, and 0 if x is zero. values: [0, 100] or "Disabled".	None
int or select	EUREQA_building_block__sine	'Sine' building block. Allows Eureqa to use the "sin()" operator in model expressions, and sets its complexity penalty. Usage: sin(x), where the angle (x) is in radians. (The standard trigonometric sine function.) values: [0, 100] or "Disabled".	None
int or select	EUREQA_building_block__square_root	'Square Root' building block. Allows Eureqa to use the "sqrt()" operator in model expressions, and sets its complexity penalty. Usage: sqrt(x), which returns the square root of x (where x must be positive). values: [0, 100] or "Disabled".	None
int or select	EUREQA_building_block__step_function	'Step Function' building block. Allows Eureqa to use the "step()" operator in model expressions, and sets its complexity penalty. Usage: step(x), which returns 1 if x is positive, 0 otherwise. values: [0, 100] or "Disabled".	None

int or select	EUREQA_building_block__subtraction	'Subtraction' building block. Allows Eureqa to use the "-" binary operator in model expressions, and sets its complexity penalty. Usage: x - y or sub(x, y), which returns the difference of x and y. values: [0, 100] or "Disabled".	None
int or select	EUREQA_building_block__tangent	'Tangent' building block. Allows Eureqa to use the "tan()" operator in model expressions, and sets its complexity penalty. Usage: tan(x), where the angle (x) is in radians. (The standard trigonometric tangent function.) values: [0, 100] or "Disabled".	None
int or select	EUREQA_building_block__two-argument_arctangent	'Two-Argument Arctangent' building block. Allows Eureqa to use the "atan2()" operator in model expressions, and sets its complexity penalty. Usage: atan2(y, x). (The standard trigonometric two-argument arctangent function.) values: [0, 100] or "Disabled".	None
int	EUREQA_max_generations	The maximum number of evolutionary generations to run. Eureqa will run until either of max_generations or timeout_sec is reached. values: [0, 1e16]	None
int	EUREQA_num_threads	The number of threads Eureqa will run with. Ideally equal to the number of cores available	None
string	EUREQA_prior_solutions	Prior Eureqa Solutions. This field contains multiple Eureqa Expressions, one per line. Each Expression should be a valid Eureqa Solution, such as a Solution returned by a previous run of Eureqa. (You may need to edit the Solution such that the target variable is entered as "Target", not the original column name of the target.) Each expression is fed into Eureqa's initial evolutionary population. Eureqa makes no guarantees about keeping the form or content of these expressions in the final Pareto front of expressions that it generates, but if the expressions are good models or if they contain sub-expressions that are predictive features, Eureqa will generally take advantage of that information to converge on good solutions more quickly. values: multiple lines, each line is a valid Eureqa Expression as a string	None
int	EUREQA_random_seed	Constant to seed Eureqa's pseudo-random number generator. Different values will cause Eureqa to generate different models on the same data and other input parameters. values: [0, 1e16]	None
select	EUREQA_split_mode	Whether to perform in-order (2) or random (1) splitting within the training set, for evolutionary re-training and re-validaton. values: [1, 2] values: [0, 1e5]	None
select	EUREQA_sync_migrations	Should Eureqa's migrations be synchronized? If they are synchronized, Eureqa's fit() function will be deterministic (repeated runs on the same data and parameters should produce the same models). Note that synchronization slows modeling down and models will take more time to generate. values: [False, True]	None

string	EUREQA_target_expression_string	Eureqa Target Expression. Constrains the form of the models that Eureqa will consider. This field is typically of the form "Target = <some Eureqa Expression>". See the description of "Eureqa Expressions". The Expression must contain an equality operator, ie., it must be a full equation with a left-hand and a right-hand side. Note that Target Expressions will usually use function operators in their expressions, as well as regular expression operators. Function operators are templates / pattern matchers. For example, "Target = f(x, y z)" will match any expression that uses the variables x, y, and/or z (but will fail to match any function that uses other variables); "Target = f1(x, y) + f2(z)" will match "x*y + z" but it will not match "x*z + y"; "Target = f(sin(x))" will match "2*sin(x)" but it will not match "sin(2*x)". This is a hard constraint. If you want to seed Eureqa with information from existing models, see prior_solutions. If this field is left blank, Eureqa will automatically generate an appropriate target expression as part of the model fitting process. This expression will be logged to the model log (visible in the UI). values: valid Eureqa Expression, as a string. Must contain an equality operator.	None
float	EUREQA_timeout_sec	The duration of time to run the Eureqa search algorithm for Eureqa will run until either of max_generations or timeout_sec is reached. values: [0, 1e16]	None
float	EUREQA_training_fraction	What fraction of the DataRobot training data to use for Eureqa evolutionary training? This field is ignored if training_split_expression is set. Note that training_fraction + validation_fraction does not have to equal 1.0. If it is less than 1.0, some rows in the data are ignored. If it is greater than 1.0, Eureqa's training and validation sets overlap. (This is not recommended, but may be required in some modeling scenarios involving small datasets. Note that DataRobot will still typically have a separate validation and holdout set.) values: [0.0, 1.0]	None
string	EUREQA_training_split_expr	Eureqa Training Split Expression. Can be any valid Eureqa Expression, including a simple variable name. The expression should evaluate to either 0.0 or 1.0. If it evaluates to 1.0 on a specific row, that row is used for training data. If this field is not set, training_fraction is used instead. Note that setting training_split_expr does not affect validation rows. If you want all non-training rows to be in the validation set, make sure to set validation_split_expr accordingly. values: valid Eureqa Expression, as a string	None
float	EUREQA_validation_fraction	What fraction of the DataRobot training data to use for Eureqa evolutionary validation? This field is ignored if validation_split_expression is set. Note that training_fraction + validation_fraction does not have to equal 1.0. If it is less than 1.0, some rows in the data are ignored. If it is greater than 1.0, Eureqa's training and validation sets overlap. (This is not recommended, but may be required in some modeling scenarios	None

		involving small datasets. Note that DataRobot will still typically have a separate validation and holdout set.) values: [0.0, 1.0]	
string	EUREQA_validation_split_expr	Eureqa Validation Split Expression. Can be any valid Eureqa Expression, including a simple variable name. The expression should evaluate to either 0.0 or 1.0. If it evaluates to 1.0 on a specific row, that row is used for validation data. If this field is not set, validation_fraction is used instead. Note that setting validation_split_expr does not impact training rows. If you want all non-validation rows to be in the training set, make sure to set training_split_expr accordingly. values: valid Eureqa Expression, as a string	None
string	EUREQA_weight_expr	Eureqa Weight Expression. Weights each row when evaluating the error of that row. The exact meaning of the weight depends on the specific value of error_metric. Typically the per-row error is multiplied by the weight before being combined with other per-row weights into the aggregate weight. If this field is left as the empty string, Eureqa falls back to DataRobot's default behavior. values: valid Eureqa Expression, as a string	None
select	XGB_base_margin_initialize	If True, the intercept is initialized to the log odds of the target. values: [False, True]	True
int	XGB_class_count	For multiclass only. The number of target classes. values: [0, MAX_TARGET_CLASS_COUNT]	None
floatgrid	XGB_colsample_bylevel	Subsample the features before each split in a tree. values: [0.1,1]	1.0
floatgrid	XGB_colsample_bytree	Subsample ratio of columns when constructing each tree. By default, the value of colsample_bytree for XGBoost classes is 1.0. However, based on the training data, DataRobot may choose a different initial value for this parameter. values: [0,1]	1.0
int	XGB_interval	Sets the interval for early stopping values: [2, 500]	None
floatgrid	XGB_learning_rate	Shrinks the contribution of each tree by learning_rate. There is a trade-off between learning_rate (lr) and n_estimators(n). values: [5e-4,1]	0.05
select	XGB_loss	loss, loss function to be optimized. 'deviance' refers to deviance (= logistic regression) for classification with probabilistic outputs. values: ['deviance', 'softprob']	None
int	XGB_max_bin	This is only used if 'hist' is specified as tree_method. Maximum number of discrete bins to bucket continuous features. Increasing this number improves the optimality of splits at the cost of higher computation time. values: [16, 2048]	256
floatgrid	XGB_max_delta_step	Maximum delta step we allow each tree's weight estimation to be. If the value is set to 0, it means there is no constraint. If it is set to a positive value, it can help making the update step more conservative. Usually this parameter is not needed, but it might help in logistic regression when class is extremely imbalanced. Set it to	0.0

		value of 1-10 might help control the update values: [0,100]	
intgrid	XGB_max_depth	maximum depth of the individual regression estimators. The maximum depth limits the number of nodes in the tree. Tune this parameter for best performance; the best value depends on the interaction of the input variables. Deeper the tree the more variable interactions the model can capture. For frozen models on larger sample sizes than parent model we increase the value of max_depth to retain similar accuracy. values: [1, 16]	3
floatgrid	XGB_min_child_weight	Minimum sum of instance weight(hessian) needed in a child. If the tree partition step results in a leaf node with the sum of instance weight less than min_child_weight, then the building process will give up further partitioning. In linear regression mode, this simply corresponds to minimum number of instances needed to be in each node. The larger, the more conservative the algorithm will be. values: [0.01,float(1e5)]	5.0
floatgrid	XGB_min_split_loss	Minimum loss reduction required to make a further partition on a leaf node of the tree. the larger, the more conservative the algorithm will be. values: [0,1e5]	0.01
float	XGB_missing_value	The float value that should be treated as a missing value. When mono_up or mono_down are set, missing value will be set to -9999.0. values: [float(-1e5),float(1e5)]	None
string	XGB_mono_down	The id of the featurelist that defines the set of features with a monotonically decreasing relationship to the target.	None
string	XGB_mono_up	The id of the featurelist that defines the set of features with a monotonically increasing relationship to the target.	None
int	XGB_n_estimators	The number of boosting stages to perform. Gradient boosting is fairly robust to over-fitting so a large number usually results in better performance. values: [1,20000]	330
intgrid	XGB_num_parallel_tree	Number of parallel trees created in each boosting stage. When this value is greater than 1, the model becomes a gradient-boosted random forest with (num_parallel_tree * n_estimators) trees. values: [1,16]	1
intgrid	XGB_random_state	The seed used in the random number generator 'values': [0, int(1e9)]	1234
multi	XGB_reg_alpha	L1 regularization term on weights, increase this value will make model more conservative. values: {'floatgrid': [0, 1e6], 'select': ['auto']}	0.0
multi	XGB_reg_lambda	L2 regularization term on weights, increase this value will make model more conservative. values: {'floatgrid': [0, 1e6], 'select': ['auto']}	1.0
float	XGB_scale_pos_weight	Scaling factor for examples in the positive class. values: [0,float(1e9)]	1.0
int	XGB_smooth_interval	Sets the minimum interval for early stopping values: [2, 1000]	None

floatgrid	XGB_subsample	subsample ratio of the training instance. Setting it to 0.5 means that XGBoost randomly collected half of the data instances to grow trees and this will prevent overfitting.	1.0
select	XGB_tree_method	The tree construction algorithm to be used. 'auto': Use heuristic to choose faster one. For small to medium dataset(<4M rows), exact greedy will be used. For very large-dataset(>=4M rows), approximate algorithm will be chosen. 'exact':Exact greedy algorithm. 'approx':Approximate greedy algorithm using sketching and histogram. 'hist': Fast histogram optimized approximate greedy algorithm. It uses some performance improvements such as bins caching. values: ['auto', 'exact', 'approx', 'hist']	auto
int	feature_interaction_max_features	Specifies the max number of one vs all interactions to include in the pairwise calculations. For example, the default value of 50 will yield (50 ^ 2 - 50) / 2 pairwise interactions if feature_interaction_threshold is zero values: [0, int(1e5)]	50
int	feature_interaction_sampling	Specifies the extent (in number of rows) of downsampling used in the interaction calculations values': [1000, int(1e6)]	2500
float	feature_interaction_threshold	Specifies the minimum value a one vs all interaction strength must have to be include a given feature in the pairwise calculations. Zero includes all and one excludes all. values: [0.0, 1.0]	0.1
int	feature_selection_max_features	The maximum number of features to include. ``values: {'int': [1, int(1e10)], 'select': ['no_limit']}`	no_limit
select	feature_selection_method	Method used to select features values: ['per_variable', 'cumulative', 'no_selection']	no_selection
int	feature_selection_min_features	The minimum number of features to include. values: [1, int(1e5)]	1
float	feature_selection_threshold	The threshold used to select features. For cumulative method, threshold is typically close to 1 in order to retain more signal (ex:0.98). For per_variable method, threshold would be close to 0 in order to discard only features with low signal. values: [0.0001, 1]	0.001
select	highdim_modeling	Whether to include high cardinality and text features. values: [False, True]	True
int	subsample	Number of rows to sample for fitting the Eureqa model. values: [1000, 1e7]	10000

extreme Gradient Boosting Classifier with Early Stopping

Ordinal scale converter of categorical features

Type	Name	Description	Best Searched
select	add_cols_metadata	If specified, add -cols to metadata. values: [False, True]	False
select	add_maps_metadata	If specified, add -maps to metadata. values: [False, True]	False

multi	card_max	Maximum number of categorical feature levels allowed. If None, a feature with any number of levels is allowed. values: {'int': [1, 9999999], 'select': None}	None
select	method	Method used in the encoding. None: uses random_scale. random: random ordering of levels, lex: lexicographical ordering by category level names, freq: frequency ordering from least frequent to most frequent, resp: response ordering. values: ['None', 'random', 'lex', 'freq', 'resp']	freq
int	min_support	Minimum number of levels required for a category to be represented on the ordinal scale. If a category level count is below the minimum, it will be grouped with other small cardinality levels or encoded as a missing value, depending on the value of other_category. values: [1, 99999]	5
int	offset	Shift the ordinal scale of ordinal encoder values: [0, 99999]	0
bool	other_category	If True, small cardinality values are mapped to a dedicated value (-1), otherwise they are encoded as missing values (-2). values: [False, True]	True
bool	random_scale	Applies if method is None. If random_scale is True, random ordering is used for the ordinal scale. If it is False, lexicographical ordering is used. values: [False, True]	True
int	seed	The RNG seed. values: [0, 99999]	1234

Median Value-Based Numeric Imputation (V2 with quick median algorithm)

Type	Name	Description	Best Searched
bool	scale_small	True if small values (range of the numeric variable is <= 1) are to be scaled. values: [False, True]	True
int	threshold	Minimum number of required finite elements in a column to impute the data onto NaNs and INFs. values: [1, 99999]	10

Prediction Model Parameters Extreme Gradient Boosting Classifier with Grid Search and Early Stopping support

Type	Name	Description	Best Searched
select	base_margin_initialize	If True, the intercept is initialized to the log odds of the target. values: [False, True]	False
int	class_count	Number of target classes (multiclass only). values: [0, MAX_TARGET_CLASS_COUNT]	None
floatgrid	colsample_bylevel	Subsample of the features before each split in a tree. values: [0.1,1]	1.0
floatgrid	colsample_bytree	Subsample ratio of columns when constructing each tree. By default, the value of colsample_bytree for XGBoost classes is 1.0. However, based on the training data, DataRobot may choose a different initial value for this parameter. values: [0,1]	1.0
int	interval	Interval for early stopping. Once the model has hit “smooth_interval”	10

		iterations, the early stopping logic checks for errors increasing at a rate of “interval” iterations in a row. For example, if smooth_interval=200 and interval=10, XGBoost will run at least 200 iterations, and will early stop if the moving average of loss for the last 200 iterations increases for 10 iterations in a row. Higher values will make XGBoost run longer. values: [2, 500]	
floatgrid	learning_rate	Shrinks the contribution of each tree by learning_rate. There is a trade-off between learning_rate (lr) and n_estimators(n). values: [5e-4,1]	0.02
select	loss	Loss function to be used during optimization. ‘deviance’ refers to deviance (= logistic regression) for classification with probabilistic outputs. values: ['deviance', 'softprob']	deviance
int	max_bin	Used when tree_method is set to ‘hist’. Maximum number of discrete bins to bucket continuous features. Increasing this number improves the optimality of splits at the cost of higher computation time. values: [16, 2048]	256
floatgrid	max_delta_step	Maximum delta step allowed for each tree’s weight estimation. If the value is set to 0, there is no constraint. Setting to a positive value makes the update step more conservative. Usually this parameter is not needed, but it might help in logistic regression when class is extremely imbalanced. Setting it to a value of 1-10 might help control the delta step update. values: [0,100]	0.0
intgrid	max_depth	Maximum depth of the individual regression estimators. The maximum depth limits the number of nodes in the tree. Tune this parameter for optimal performance; the best value depends on the interaction of the input variables. The deeper the tree, the more variable interactions the model can capture. For frozen models that have larger sample sizes than the parent model, the max_depth value is increased to retain similar accuracy. values: [1, 16]	1
floatgrid	min_child_weight	Minimum sum of instance weight (hessian) needed in a child. If the tree partition step results in a leaf node with the sum of instance weight less than min_child_weight, the building process will give up further partitioning. In linear regression mode, this simply corresponds to the minimum number of instances needed to be in each node. The larger the value, the more conservative the algorithm will be. values: [0.01,float(1e5)]	1.0
floatgrid	min_split_loss	Minimum loss reduction required to make a further partition on a leaf node of the tree. The larger the value, the more conservative the algorithm will be. values: [0,1e5]	0.01
float	missing_value	Float value that should be treated as a missing value. When mono_up or mono_down is set, missing value will be set to -9999.0. values: [float(-1e5),float(1e5)]	-9999.0
string	mono_down	ID of the featurelist that defines the set of features with a monotonically decreasing relationship to the target.	no

string	mono_up	ID of the featurelist that defines the set of features with a monotonically increasing relationship to the target.	no
int	n_estimators	Number of boosting stages to perform. Gradient boosting is fairly robust to overfitting, so a larger number usually results in better performance. values: [1,20000]	210
intgrid	num_parallel_tree	Number of parallel trees created in each boosting stage. When this value is greater than 1, the model becomes a gradient-boosted random forest with (num_parallel_tree * n_estimators) trees. values: [1,16]	1
intgrid	random_state	Seed used in the random number generator 'values': [0, int(1e9)]	1234
multi	reg_alpha	L1 regularization term on weights; increasing this value will make the model more conservative. values: {'floatgrid': [0, 1e6], 'select': ['auto']}	0.0
multi	reg_lambda	L2 regularization term on weights. Increasing this value will make the model more conservative. values: {'floatgrid': [0, 1e6], 'select': ['auto']}	1.0
float	scale_pos_weight	Scaling factor for examples in the positive class. values: [0,float(1e9)]	1.0
int	smooth_interval	Moving average interval for early stopping. Loss over the last n intervals is averaged for determining early stopping. For example, if smooth_interval=200, XGBoost will not stop early until it hits at least 200 iterations. After 200 iterations, it will use a moving average of loss over the last 200 iterations to determine early stopping. This helps remove noise in the loss function. Higher values will make XGBoost run longer. values: [2, 1000]	200
floatgrid	subsample	Subsample ratio of the training instance. Setting it to 0.5 means that XGBoost randomly collected half of the data instances to grow trees, which will prevent overfitting.	0.8
select	tree_method	Tree construction algorithm to use. 'auto': Heuristic to choose the faster algortihm. For small to medium datasets (<4M rows), exact greedy will be used. For large datasets (>=4M rows), approximate algorithm will be used. 'exact':Exact greedy algorithm. 'approx':Approximate greedy algorithm using sketching and histogram. 'hist':Fast histogram-optimized approximate greedy algorithm. It uses some performance improvements (e.g., bin caching). values: ['auto', 'exact', 'approx', 'hist']	auto

Dropout Additive Regression Trees Classifier (15 leaves)

Ordinal scale converter of categorical features

Type	Name	Description	Best Searched
select	add_cols_metadata	If specified, add -cols to metadata. values: [False, True]	False
select	add_maps_metadata	If specified, add -maps to metadata. values: [False, True]	False

multi	card_max	Maximum number of categorical feature levels allowed. If None, a feature with any number of levels is allowed. values: {'int': [1, 9999999], 'select': None}	None
select	method	Method used in the encoding. None: uses random_scale. random: random ordering of levels, lex: lexicographical ordering by category level names, freq: frequency ordering from least frequent to most frequent, resp: response ordering. values: ['None', 'random', 'lex', 'freq', 'resp']	freq
int	min_support	Minimum number of levels required for a category to be represented on the ordinal scale. If a category level count is below the minimum, it will be grouped with other small cardinality levels or encoded as a missing value, depending on the value of other_category. values: [1, 99999]	5
int	offset	Shift the ordinal scale of ordinal encoder values: [0, 99999]	0
bool	other_category	If True, small cardinality values are mapped to a dedicated value (-1), otherwise they are encoded as missing values (-2). values: [False, True]	True
bool	random_scale	Applies if method is None. If random_scale is True, random ordering is used for the ordinal scale. If it is False, lexicographical ordering is used. values: [False, True]	True
int	seed	The RNG seed. values: [0, 99999]	1234

Median Value-Based Numeric Imputation (V2 with quick median algorithm)

Type	Name	Description	Best Searched
bool	scale_small	True if small values (range of the numeric variable is <= 1) are to be scaled. values: [False, True]	True
int	threshold	Minimum number of required finite elements in a column to impute the data onto NaNs and INFs. values: [1, 99999]	10

Prediction Model Parameters LightGBM Dropout Additive Regression Trees Classifier

Type	Name	Description	Best Searched
floatgrid	colsample_bytree	Subsample ratio of columns when constructing each tree. By default, the value of colsample_bytree for LightGBM classes is 1.0. However, based on the training data, DataRobot may choose a different initial value for this parameter. values: [0, 1]	0.3
floatgrid	drop_rate	Dropout rate. values: [0, 1]	0.1
intgrid	drop_seed	Used to random seed to choose dropping models. values: [0, 1e2]	4
select	is_unbalance	Set to true if training data are unbalanced. Used in binary classification. values: [True, False]	False
floatgrid	learning_rate	Shrink the contribution of each tree by learning_rate. There is a trade-off between learning_rate (lr) and	0.5

		n_estimators(n). In dart, it also affects normalization values: [1e-7, 1e2]	
intgrid	max_bin	Max number of bin that feature values will bucket in. Small bin may reduce training accuracy but may increase general power (deal with overfit). LightGBM will auto compress memory according max_bin. For example, LightGBM will use uint8_t for feature value if max_bin=255. values: [3, 1e4]	255
intgrid	max_depth	Maximum depth of the individual regression estimators. The maximum depth limits the number of nodes in the tree. Tune this parameter for best performance; the best value depends on the interaction of the input variables. Deeper the tree the more variable interactions the model can capture. Tree still grow by leaf-wise. <0 means no limit values: ['none', [1, 1e4]]	none
intgrid	max_drop	Max number of dropped trees on one iteration. <=0 means no limit. values: ['auto', [1, 1e3]]	50
int	min_child_samples	Minimum number of data need in a child(leaf). values: [0, 1e3]	10
intgrid	min_child_weight	Minimum sum of instance weight(hessian) needed in a child(leaf). values: [0, 1e2]	5
floatgrid	min_split_gain	Minimum loss reduction required to make a further partition on a leaf node of the tree. values: [0, 100]	0.0
intgrid	n_estimators	Number of boosting stages to perform. Gradient boosting is fairly robust to overfitting so a large number usually results in better performance. values: [1, 1e6]	10
intgrid	num_leaves	Number of leaves in one tree. values: [2, 1e4]	15
floatgrid	reg_alpha	L1 regularization term on weights. values: [0, 1e6]	0.0
floatgrid	reg_lambda	L2 regularization term on weights. values: [0, 1e6]	0.0
floatgrid	sigmoid	Parameter for sigmoid function. Used in binary classification and LambdaRank. values: [1e-06, 1e03]	1.0
floatgrid	skip_drop	Probability of skipping drop. values: [0, 1]	0.5
floatgrid	subsample	Subsample ratio of the training instance. values: [0.01, 1]	1.0
int	subsample_for_bin	Number of samples for constructing bins. values: [1, 1e6]	50000
intgrid	subsample_freq	Frequency of subsample 'none' means it is not enabled. values: ['none', [1, 1e3]]	1
select	uniform_drop	True if want to use uniform drop. values: [True, False]	True
select	xgboost_dart_mode	True if want to use xgboost dart mode. values: [True, False]	True
