# Supplementary material for: Using Automated Machine Learning to Predict Necessary Upcoming Therapy Changes in Patients With Psoriasis Vulgaris and Psoriatic Arthritis and Uncover New Influences on Disease Progression: Retrospective Study
Source: JMIR Form Res. 2024 Jun 27;8:e55855. doi: 10.2196/55855 (PMC11240079; doi:10.2196/55855)
Supplement: Multimedia Appendix 10 [file formative_v8i1e55855_app10.pdf]

Multimedia Appendix 10

Blueprints of the selected models used during AutoML analysis of targets 1 to 3

(a)

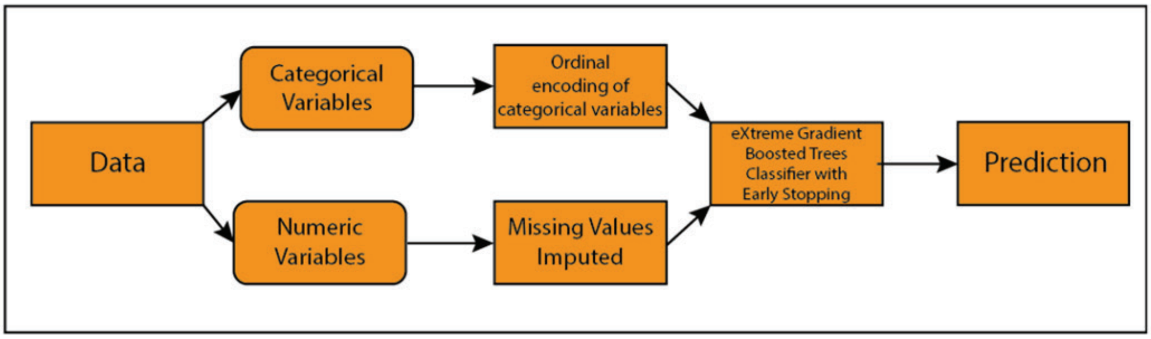

(b)

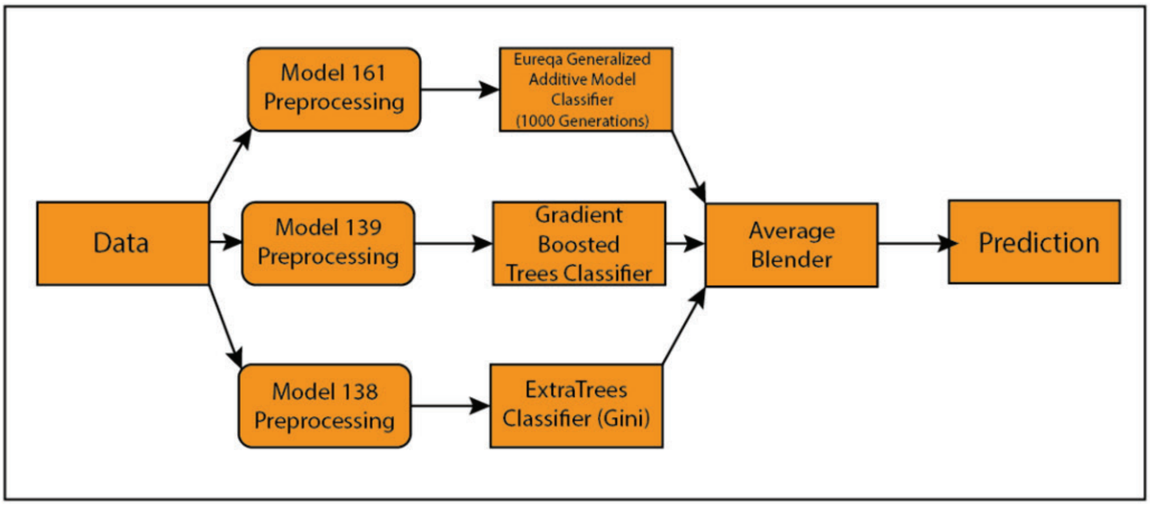

(c)

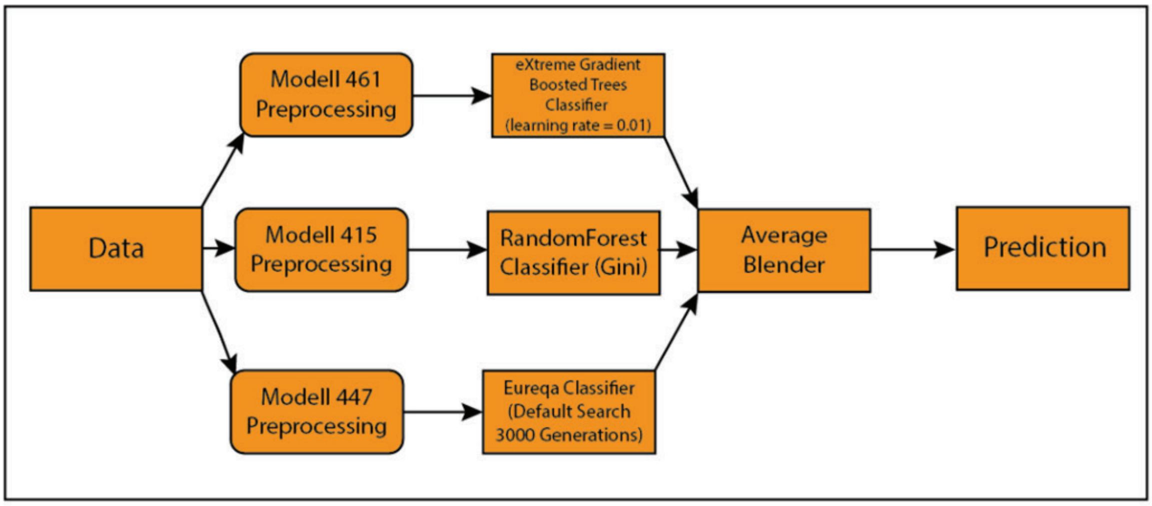

(d)

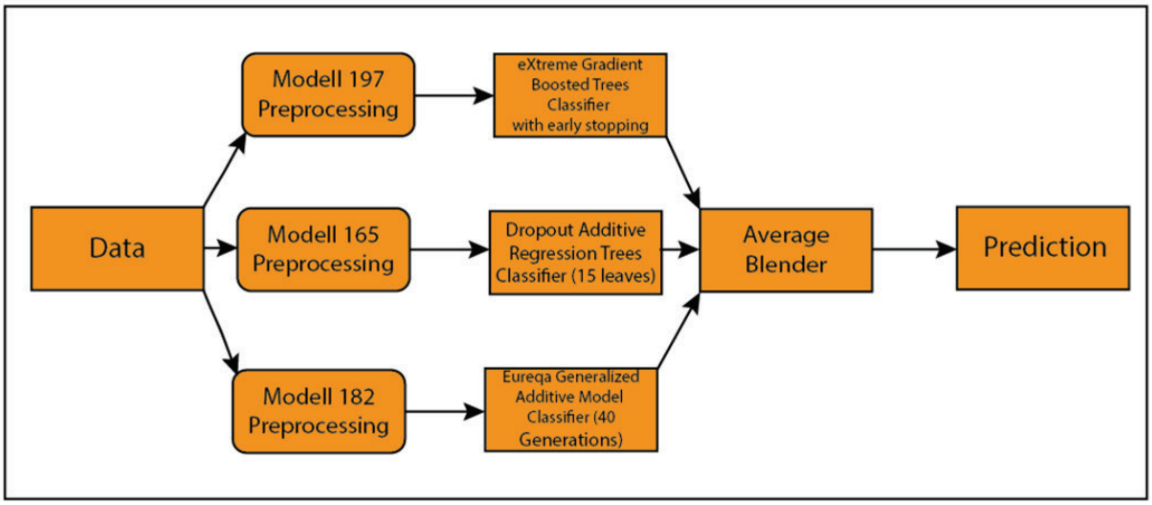

The individual steps of model building are shown schematically. (a) Target 1.1: "Therapy change at 24 weeks follow-up" analysis using baseline, and 24-week follow-up features. (b) Target 1.2: "Therapy change prediction (only onset features)" analysis using baseline characteristics only for a prediction model. (c) Target 2: "PASI score change after 24 weeks" analysis using baseline, and 24-week follow-up features. (d) Target 3: "BASDAI classification at onset" analysis using baseline features.
