# Supplementary material for: Using Automated Machine Learning to Predict Necessary Upcoming Therapy Changes in Patients With Psoriasis Vulgaris and Psoriatic Arthritis and Uncover New Influences on Disease Progression: Retrospective Study
Source: JMIR Form Res. 2024 Jun 27;8:e55855. doi: 10.2196/55855 (PMC11240079; doi:10.2196/55855)
Supplement: Multimedia Appendix 11 [file formative_v8i1e55855_app11.pdf]

Multimedia Appendix 11

Assessment of model performance and learning dynamics for each selected model

Mean LogLoss across all cross-validation folds

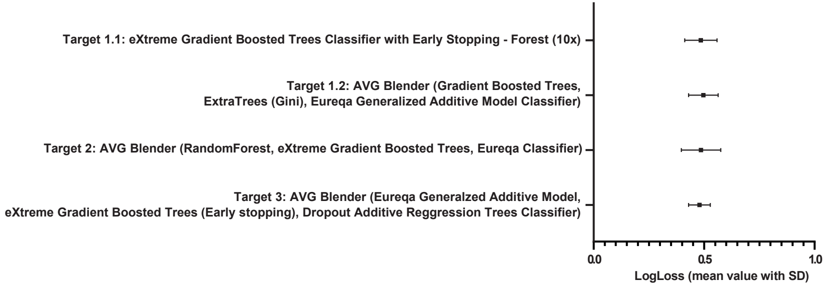

Learning Curves (mean metric score / 95% Confidence Intervall)

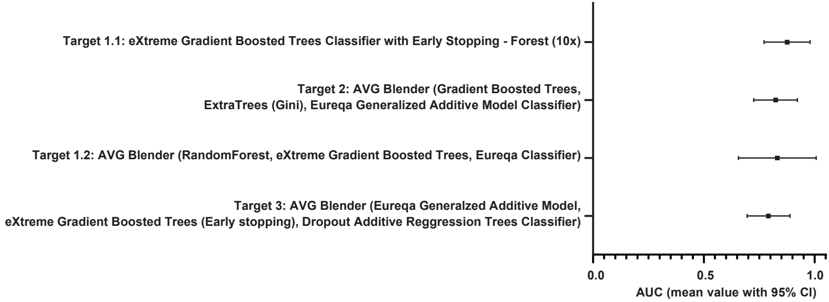

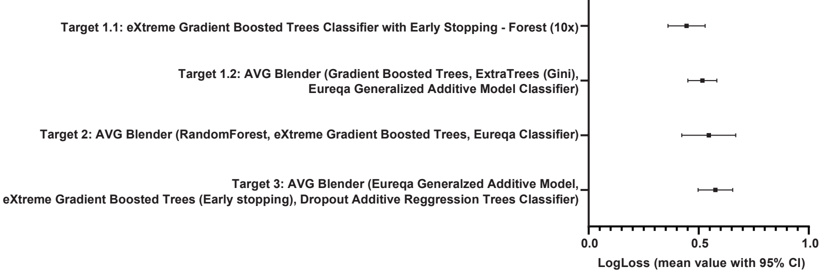

Mean LogLoss values with standard deviations on all cross-validation folds for the selected models. Learning curves (LogLoss and AUC on the holdout) are reported as an mean values with 95% confidence intervals indicating the predictive range. The selected models were trained with sample sizes of 16, 32, 64 and 80%.
