# Supplementary material for: Using Automated Machine Learning to Predict Necessary Upcoming Therapy Changes in Patients With Psoriasis Vulgaris and Psoriatic Arthritis and Uncover New Influences on Disease Progression: Retrospective Study
Source: JMIR Form Res. 2024 Jun 27;8:e55855. doi: 10.2196/55855 (PMC11240079; doi:10.2196/55855)
Supplement: Multimedia Appendix 12 [file formative_v8i1e55855_app12.pdf]

Multimedia Appendix 12

Comparative analysis of the performance metrics of the 'eXtreme Gradient Boosted Trees Classifier' model selected for Target 1.1 using 10x different seeds for data partitioning

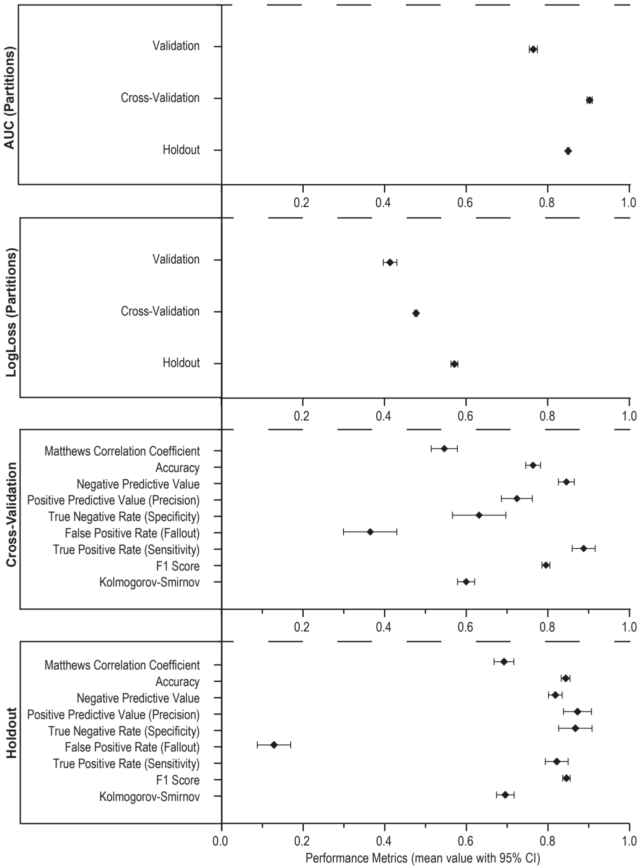

Using a methodology that includes 10 different seed values for data partitioning, the model's performance was reevaluated across validation, cross-validation and holdout partitions. Seeds are random number generators used to shuffle the data prior to partitioning into training, validation and holdout sets. Using different randomly selected seeds, the selected model was run 10 times on differently shuffled partition sets, allowing the variability and stability of model performances across these different data splits to be assessed. LogLoss and AUC are presented for all partitions, while Matthews correlation coefficient (MCC), accuracy, negative predictive value (NPV), positive predictive value (precision), true negative rate (specificity), false positive rate (fallout), true positive rate (sensitivity), F1 score and Kolmogorov-Smirnov (KS) index are presented for the cross-validation and holdout partitions, with accompanying mean values and 95% confidence intervals.
