# Supplementary material for: Using Automated Machine Learning to Predict Necessary Upcoming Therapy Changes in Patients With Psoriasis Vulgaris and Psoriatic Arthritis and Uncover New Influences on Disease Progression: Retrospective Study
Source: JMIR Form Res. 2024 Jun 27;8:e55855. doi: 10.2196/55855 (PMC11240079; doi:10.2196/55855)

Multimedia Appendix 17

Holdout partition ROC curves

(S1) Target 1.1: Therapy change at 24 weeks follow-up

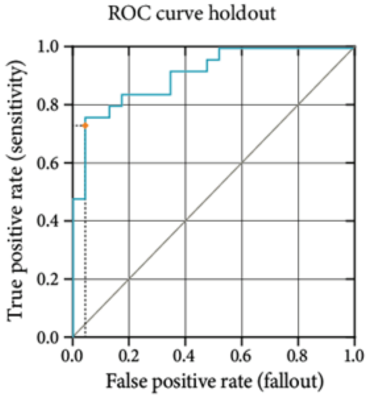

(S2) Target 2: PASI change after 24 weeks

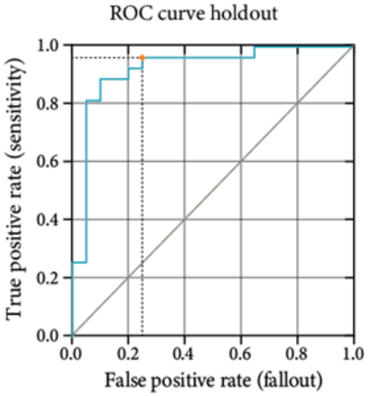

(S3) Target 3: BASDAI classification at onset

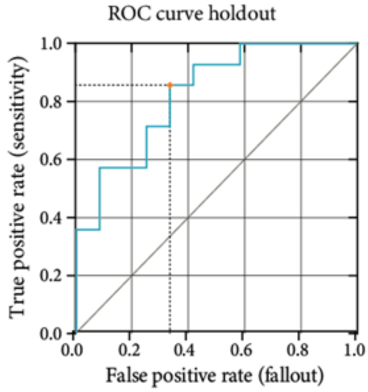

Supplement: Multimedia Appendix 17 [file formative_v8i1e55855_app17.pdf]
