# Supplementary material for: Using Automated Machine Learning to Predict Necessary Upcoming Therapy Changes in Patients With Psoriasis Vulgaris and Psoriatic Arthritis and Uncover New Influences on Disease Progression: Retrospective Study
Source: JMIR Form Res. 2024 Jun 27;8:e55855. doi: 10.2196/55855 (PMC11240079; doi:10.2196/55855)
Supplement: Multimedia Appendix 18 [file formative_v8i1e55855_app18.pdf]

Multimedia Appendix 18

Therapy change prediction scenario for two hypothetical psoriasis patients

**Individual patient related features**	**Patient 1**	**Patient 2**
Gender	w	w
Age	47	47
Body height	175	175
Bodyweight	89	89
BMI	29	29
Smoking	1	1
Alcohol	0 days/week	0 days/week
Occupation	mostly sedentary	mostly sedentary
Sports	1	1
Physical activity at onset	only sports	only sports
**Treatment Features**		
Systemic treatment at onset	Adalimumab	Adalimumab & systemic steroids
Systemic target at onset	TNF-α	TNF-α & others
Therapy with IL-17 Inhibitors at onset	0	0
Therapy with IL-12/23 Inhibitors at onset	0	0
Therapy with IL-23 Inhibitors at onset	0	0
Therapy with TNF-α inhibitors at onset	1	1
Therapy with csDMARDs at onset	0	0
Therapy with others then b-/csDMARDs	0	1
Topical therapy at onset	1	1
**Disease related Features**		
Pain (NRS) at onset	4	4
Pruritus (NRS) at onset	3	3
Disease activity (NRS) at onset	4	4
PASI score at onset	10	10
CASPAR score at onset	3	3
CASPAR classification at onset	1	1
BASDAI score at onset	4	4
BASDAI classification at onset	1	1
HADS-A classification at onset	borderline	borderline
HADS-D classification at onset	suspect	suspect
DLQI classification at onset	medium influence	medium influence
Prediction result	0.5097	0.6758
**Prediction result interpretation**	**negative**	**positive**

Overview of features of hypothetical patients used to predict an expected change in therapy. Gray shaded areas contain simplified binary information (0 = no; 1 = yes) derived from the other numeric or categorical features. The 2 patients differ only in their systemic therapy. While patient 1 receives only adalimumab, in patient 2 this is combined with systemic steroids. The differences are highlighted in red. The predicted results are related to the selected threshold of 0.5463.
