# Supplementary material for: Using Automated Machine Learning to Predict Necessary Upcoming Therapy Changes in Patients With Psoriasis Vulgaris and Psoriatic Arthritis and Uncover New Influences on Disease Progression: Retrospective Study
Source: JMIR Form Res. 2024 Jun 27;8:e55855. doi: 10.2196/55855 (PMC11240079; doi:10.2196/55855)
Supplement: Multimedia Appendix 19 [file formative_v8i1e55855_app19.pdf]

# Multimedia Appendix 19

List of feature effects of the selected average blender model for Target 2 "PASI change after 24 weeks"

| Feature name | Impact |
|---|---|
| PASI score at onset | 1,000 |
| Pain change over 24 weeks | 0,303 |
| Therapy change differential | 0,135 |
| Pain (NRS) at onset | 0,114 |
| Body weight at onset | 0,099 |
| Body height at onset | 0,098 |
| Pruritus (NRS) at onset | 0,090 |
| HADS-D score at onset | 0,087 |
| BMI at onset | 0,080 |
| HADS-A score at onset | 0,076 |
| DLQI score at onset | 0,068 |
| Age | 0,053 |
| Systemic target at onset | 0,051 |
| Occupation | 0,048 |
| Therapy with TNF-α inhibitors at onset | 0,036 |
| DLQI classification at onset | 0,033 |
| Alcohol | 0,032 |
| Pain change over 24 weeks | 0,031 |
| Disease activity (NRS) at onset | 0,031 |
| BASDAI score at onset | 0,026 |
| DLQI classification change over 24 weeks | 0,023 |
| Gender | 0,021 |
| Systemic treatment at onset | 0,021 |
| Physical activity at onset | 0,018 |
| Topical therapy duration over 24 weeks | 0,017 |
| Smoking | 0,013 |
| HADS-A classification at onset | 0,012 |

| | |
|---|---|
| CASPAR score at onset | 0,012 |
| Topical therapy at onset | 0,010 |
| Diagnosed other disease at onset | 0,009 |
| Therapy with others then b-/csDMARDs | 0,009 |
| App used | 0,009 |
| HADS-A classification change over 24 weeks | 0,008 |
| Therapy change binary | 0,007 |
| CASPAR classification at onset | 0,007 |
| Sports | 0,006 |
| HADS-D classification change over 24 weeks | 0,006 |
| Therapy with csDMARDs at onset | 0,005 |
| Therapy with IL-12 /23 inhibitor at onset | 0,005 |
| HADS-D classification at onset | 0,004 |
| BASDAI classification at onset | 0,003 |
| Diagnosed arterial hypertension at onset | 0,003 |
| Diagnosed depression at onset | 0,002 |
| Obesity at onset | 0,002 |
| No diagnosed pre-existing illness at onset | 0,002 |
| Diagnosed metabolic disease at onset | 0,002 |
| Therapy with IL-17 Inhibitors at onset | 0,002 |
| Therapy with IL-23 inhibitors at onset | 0,000 |
| Patient_ID | 0,000 |
| Diagnosed coronary heart disease at onset | 0,000 |
